# Salt tolerance in rice: seedling and reproductive stage QTL mapping come of age

**DOI:** 10.1007/s00122-021-03890-3

**Published:** 2021-07-21

**Authors:** Rakesh Kumar Singh, Suneetha Kota, Timothy J. Flowers

**Affiliations:** 1 Present Address: Crop Diversification and Genetics, International Center for Biosaline Agriculture (ICBA), Dubai, UAE; 2 Rice Breeding Platform, International Rice Research Institute (IRRI), Los Banos, Philippines; 3 Present Address: Genetics and Plant Breeding Department, Indian Institute of Rice Research (IIRR), Hyderabad, India; 4 School of Life Sciences, University of Sussex, Brighton, BN1 9QG UK

## Abstract

**Key message:**

Reproductive stage salinity tolerance is most critical for rice as it determines the yield under stress. Few studies have been undertaken for this trait as phenotyping was cumbersome, but new methodology outlined in this review seeks to redress this deficiency. Sixty-three meta-QTLs, the most important genomic regions to target for enhancing salinity tolerance, are reported.

**Abstract:**

Although rice has been categorized as a salt-sensitive crop, it is not equally affected throughout its growth, being most sensitive at the seedling and reproductive stages. However, a very poor correlation exists between sensitivity at these two stages, which suggests that the effects of salt are determined by different mechanisms and sets of genes (QTLs) in seedlings and during flowering. Although tolerance at the reproductive stage is arguably the more important, as it translates directly into grain yield, more than 90% of publications on the effects of salinity on rice are limited to the seedling stage. Only a few studies have been conducted on tolerance at the reproductive stage, as phenotyping is cumbersome. In this review, we list the varieties of rice released for salinity tolerance traits, those being commercially cultivated in salt-affected soils and summarize phenotyping methodologies. Since further increases in tolerance are needed to maintain future productivity, we highlight work on phenotyping for salinity tolerance at the reproductive stage. We have constructed an exhaustive list of the 935 reported QTLs for salinity tolerance in rice at the seedling and reproductive stages. We illustrate the chromosome locations of 63 meta-QTLs (with 95% confidence interval) that indicate the most important genomic regions for salt tolerance in rice. Further study of these QTLs should enhance our understanding of salt tolerance in rice and, if targeted, will have the highest probability of success for marker-assisted selections.

**Supplementary Information:**

The online version contains supplementary material available at 10.1007/s00122-021-03890-3.

## Introduction

Biotic and abiotic stresses adversely affect crop growth and productivity. In crops, these abiotic stresses are generated by environmental factors such as drought, salinity and alkalinity, nutrient toxicity or deficiency, flooding and poor drainage, high or low soil pH, high and low temperatures and heavy metals; all are complex and often interacting phenomena and limit crop production worldwide (Shahbaz and Ashraf 2013; Almeida et al. 2016). Of these abiotic stresses, drought and salinity have a major impact on the productivity of a number of crops, including rice. For rice, salinity is next only to drought in limiting its productivity. Indeed, frequent occurrences of the combination of drought, due to declining water resources, and salinity, often due to poor irrigation management (Raes et al. 1995; Glick et al. 2007), have created a situation where rice ecosystems are now highly vulnerable to climate change. In addition, intrusion of sea water in coastal areas is converting arable lands to saline soils, while climatic conditions such as air humidity also affect the severity of salinity (Asch et al. 1995, 1997a, b). In this review, we summarize recent advances in understanding salinity tolerance in rice with particular emphasis on stage-specific tolerance. This review emphasises recent developments in available phenotyping methods for salt stress screening at different crop growth stages. Emphasis has been placed on a phenotyping protocol for reproductive stage salinity tolerance, as this has been most problematic for researchers. We have also reviewed QTL mapping studies and hotspots for effective introgressions of candidate genes, together with the application of marker-assisted selection (MAS) for developing commercial rice varieties suitable for salt-affected areas across the world. Potential candidate genes associated with salinity tolerance in the identified meta-QTL regions are also discussed.

Salinity, as far as soils are concerned, refers to the presence of soluble salts above an arbitrary limit, commonly defined by the electrical conductivity (EC) of a saturated soil paste. Agronomically, soil salinity is defined as the presence of sufficient soluble salts in the soil to reduce normal crop growth (Bockheim and Gennadiyev 2000), but this concentration varies from one crop to another and for different varieties within a species. The excess salts are commonly in the form of chlorides and sulfates of sodium and magnesium. Generally, problem soils due to salts are referred to as saline, sodic and saline-sodic based on their EC, exchangeable sodium percentage (ESP) and pH. Soils are termed ‘saline’ if the EC is more than 4 dS m^−1^ (see Ghassemi et al. 1995). If sodium (Na^+^) predominates with bicarbonate and carbonate anions, the soils are termed ‘sodic’ and are characterized by very poor soil structure that dramatically reduces water infiltration and drainage. Saline soils will have an EC > 4 dS m^−1^ and ESP < 15 with pH < 8.8; sodic soils have an EC < 4 dS m^−1^ and ESP > 15 percent with pH 8.5 to 10.7 while ‘saline-sodic’ soils will have characteristics of saline and sodic soils: EC > 4 dS m^−1^ and ESP > 15 percent with variable pH (USSL Staff 1954; Eynard et al. 2005).

Soil salinity is known to influence about 20% of the earth’s land and is relatively more widespread in arid and semi-arid climates compared to humid regions. The association with aridity leads to a link with irrigation: salinization affects about 50% of irrigated land worldwide, which includes about 30% of the rice areas (Wang et al. 2012). Globally one-fifth of the world’s arable land and one-third of irrigated agricultural area is salt-affected and has been estimated to be increasing at a very rapid pace (Machado and Serralheiro 2017; Collins 2014). About 30% of the world’s rice growing land is affected by soil salinity (Ahmad and Prasad, 2011; Wang et al. 2012; Hopmans et al. 2021).

### Effects of salinity on various growth stages of rice

Following investigation of a few genotypes, rice was categorized as a ‘sensitive’ crop with a threshold salinity of 3 dS m^−1^ (Maas and Hoffman 1977) among the four categories of ‘tolerant,’ ‘moderately tolerant,’ ‘moderately sensitive’ and ‘sensitive’ to salinity. However, we now know that rice possesses a large variability for salt tolerance (Singh and Flowers 2010; Munns et al. 2006; Sabouri and Biabani 2009; Negrao et al. 2011; De Leon et al. 2015), variability that can be accessed in collections of rice germplasm that exist throughout world (e.g., the International Rice Research Institute (IRRI) alone has more than 129,000 accessions stored in its Germplasm Resource Centre). So we now classify rice as moderately tolerant to salinity since numerous rice genotypes fall into this category (see Table 1). Similarly, sodicity stress can be classified as low, moderate and high (Table 2) with rice again showing a considerable variation between cultivars. Under sodic conditions at pH 9.8, grain yield reduction of 25%, 37%, and 68% has been reported for tolerant, semi-tolerant and sensitive rice cultivars, respectively (Rao et al. 2008).

**Table 1:** List of lines of rice tolerant to salinity that have been used in research studies and developmental breeding programs

Salt-tolerant line	References
Pokkali, Nona Bokra, Bicol	Xie et al. (2000)
TCCP 266-2-49-B-B-3, IR51500-AC11-1, IR51500-AC17, IR51485-AC6534-4, IR72132-AC6-1, IR69997-AC1, IR69997-AC2, IR69997-AC3, R69997-AC4,	Senadhira et al. (2002)
Cuom	Hien et al. (2003)
IR 65195, PSBRC 50, Nona Bokra, At 401, BW 451, At354, Pokkali, Jhona 349, IR 4630, BW 351, IR 51500, Kombila, BW 302, Kharamana, IR 1721, Matarawee, Moddikarruppan, Pokkalian, Baticoloa, OB 678, SR 26B, Lankasamurdi, Uvarkarruppan	Safeena et al. (2003)
Cheriviruppu (IRGC 19928), Kalimekri 77-5 (IRTP 14213), TKM6 (IRTP 11703), Bhura Rata (IRGC 28590), Mushkan 41 (IRGC 6828), Kalarata 1-24 (IRGC 26913), Bhirpala (IRGC 37015), IR4630-22-2-5-1-3 (IRGC72958), Kajalsail, IR69502-6-SRN-3-UBN-1-B, IR65483-118-25-31-7-1-5, IR65483-141-2-4-4-2-5, IR77298-14-1-2, IR63262-AC201-1-7-2, IR73689-76-2	Adorada et al. (2005)
Pokkali, Dang Dawk Kok, Luang Ta Moh, Supanburi 2	Theerakulpisut et al. (2005)
SAL 187 (IR65209-3b-6-3-1), SAL 271 (IR65858-4B-11-1-2), SAL 345 (IR69588-4R-P-11-3), SAL 518 (IR72046-B-R-7-3-1-2), SAL 534 (IR71832-3R-2-2-1), SAL 543 (IR71899-2-1-1), SAL 546 (IR71991-3R-2-6-1), SAL 547( IR71995-3R-1-2-2), SAL 669 (IR74099-3R-3-3), SAL 699(IR74105-3R-2-1), SAL 729 (IR70023-4B-R-12-3-1), IRGC 19928(Chervirrupu), IRGC 26913(Kalarata 1-24), IRGC 108921(Pokkali), FL 478 (IR66946-3R-178-1-1), SAL 411 (IR72046-B-R-4-3-2-1-2B-1), ShahPasand (Iranian Variety)	Mohammadi-Nejad et al. (2008)
Mantaro rice, Guandong 51, Binre, Zhuziqing, Lansheng, IR 46, IR 4422-28-5, Pokkali, Kalarata 1-24 (IRGC 26913), Bhura Rata (IRGC 28590), BR 1, BR 203-26-2, Sail, Jingnuo 8, Linyi tangdao, Bairizao, Xiaojingdao, Cun-an lengshuibai, 80-85, Zhuxi 26, Sunuo 1, Zhengxian 139, Nanjing 570, Haoanxie, Zhuguang 23, Zhuguang 29, Taihuzao, Aijiaolaolaiqing, Jiucaiqing, Maxiangu, Maodao, Hongmangxiangjingnuo, Erzaobaigu, Hongkenuo, Meimanggui, Longjianghong, Dahonggu, Huangjingnuo, Dayanggu, Yingyang 1, Xigu, Wanmanzao, Shengshuilian, Xianzhan, Damangdao, Laohuangdao, Gaoliangdao, Liaoyan 2, Changbai 7	Hu et al. (2012)
Ketumbar, Khao Seetha, Soc Nau, Damodar (CSR 1), Dasal (CSR 2), Getu (CSR 3), Pokkali, Vytilla 1, Vytilla 2, Vytilla 3, Vytilla 4, Vytilla 5	Amaranatha et al. (2014)
SADRI (IRGC acc. 32329), FL478 (IR66946-3R-178-1-1), CSR28, IR4630-22-2-5-1-3, R70023-4B-R-12-3-1, SADRI (IRGC acc. 32329)	Mohammadi et al. (2014)
Cheriviruppu	Hossain et al. (2015)
Tarome-Molaei, Nona Bokra, Moroberekan	Khan et al. (2016)

The results of these studies (i.e., developed varieties) are shown in Table 7

**Table 2:** Relative salt tolerance of different growth stages of rice

Kind of salt stress	Growth stage	Low	Moderate	High
Salinity—EC_e_ (dS m^−1^)	Seedling	< 6	6–10	> 10
	Reproductive	< 6	6–8	> 8
Sodicity (pH_1:2_)	Seedling	< 9.2	9.2–9.8	> 9.8
	Reproductive	< 9.2	9.2–9.6	> 9.6

The figures in the table show the electrical conductivity of a saturated soil paste and the pH of a 1:2 soil water paste that define different levels of tolerance (low, moderate and high) to salinity and sodicity

Here, EC_e_ is the electrical conductivity of a saturated soil paste; and pH_1:2_ is the pH of a stirred mixture of 1 part of soil and 2 parts of distilled water

Rice plants respond differently to salt stress at different growth stages (see Moradi and Ismail 2007; Singh and Flowers 2010). Over the entire growth period, rice is relatively tolerant at germination, but growth becomes very sensitive during the early seedling stage (1–3 weeks), and then more tolerant during active tillering. The most sensitive stage as far as overall grain yield is concerned is from panicle initiation to flowering and fertilization. The plants are relatively more tolerant at maturity (Khatun and Flowers 1995; Khan et al. 1997; Folkard and Wopereis 2001; Singh et al. 2004, 2008; Shereen et al. 2005; Agnihotri et al. 2006; Hakim et al. 2010; Ologundudu et al. 2014; Sajid et al. 2017). Hence, tolerance at the seedling and reproductive stages in rice are critical issues for breeding a salt-tolerant rice variety and for the management of rice productivity in the field (Zeng et al. 2001; Ahmadizadeh et al. 2016; Sajid et al. 2017).

### Effects of salinity at germination, seedling and vegetative stages

Although rice is very sensitive at its seedling and reproductive stages, it is relatively tolerant at other growth stages including one of the shortest germination. In some cases, germination, which last for 2–3 d, is reportedly not significantly affected up to 16 dS m^−1^ (Khan et al.^1997^). Ologundudu et al. (2014) reported that salinity did not affect germination (80% germinating) up to 10 dS m^−1^ in rice genotypes tolerant at the seedling stage, while in sensitive genotypes germination was only reduced to 50 percent. At 5 dS m^−1^, both seedling-stage tolerant and sensitive genotypes recorded up to 90 percent germination (Ologundudu et al. 2014). However, irrespective of genotypes, salt stress reduced the rate of germination (Khan et al. 1997; Folkard and Wopereis 2001; Hakim et al. 2010; Ologundudu et al. 2014).

The effects of salinity at the seedling and early vegetative stages are well documented (see Singh and Flowers 2010), with wide variability existing among germplasm lines (Negrao et al. 2013; Islam et al. 2012; Al-Amin et al. 2013; Babu et al. 2014). Most of the tolerant varieties were developed utilizing a limited number of resistant donors such as Nona Bokra or Pokkali or varieties derived from parents such as CSR 28 (Negrao et al. 2011): many of the identified sources of tolerance are landraces. Additionally, wild species can be explored for salinity tolerance mechanisms and new donors (Solis et al. 2020). Investigations of the variability among wild and cultivated rice species in response to salinity found that cultivars derived from crosses of *O. glaberrima* and *O. sativa* confer low Na^+^ and high K ^+^ concentrations in roots and shoots. Among the wild sources, *O. rufipogon* with its high compatibility with *O. sativa* is widely used for breeding salt-tolerant lines. Lines developed from *O. rufipogon* and *O. sativa* hybridization showed nine quantitative trait loci (QTL) and candidate genes (e.g., HKT1;5, HAK6) controlling salt tolerance at the seedling stage (Quan et al. 2018). Among other wild species, *O. coarctata* was found to be the most tolerant wild relative, followed by *O. latifolia* and *O. alta* (Prusty et al. 2018); all can be targeted in genetic improvement programs to develop salt-tolerant cultivars (Solis et al. 2020). New donors identified in various studies for seedling or egetative-stage tolerance listed in Table 1 will help to broaden the gene pool and hasten the pace of breeding of rice for salinity tolerance. This will also provide an opportunity to utilize the broad spectrum of available genetic resources in various region-specific breeding programs to develop tailor-made rice varieties designed for specific locations.

### Effects of salinity at the reproductive stage: effects on yield and yield components

At the field level, the effects of salt stress during the reproductive stage are more important than at the vegetative stage (Rao et al. 2008) with the most deleterious effect on yield being stress during panicle initiation (PI) before booting. A significant reduction in tiller number per plant is observed if plants are exposed to salt stress before PI (Zeng et al. 2001); salinity at PI reduced yield by 50% (Zeng et al. 2002) to 80% (Asch and Wopereis 2001). Asch and Wopereis (2001) reported a yield loss for sensitive genotypes of 1 t ha^−1^ per unit EC (dS m^−1^) with water EC levels > 2 dS m^−1^, while yield loss for tolerant cultivars was less than 0.6 t ha^−1^ per unit increase in EC (genotypes used had yields of approximately 8 t ha^−1^ when irrigated with fresh water). In other reports, a 12% yield reduction per dS m^−1^ has been observed at salinities above a threshold level of 3 dS m^−1^ (Zeng et al. 2002).

Reductions in grain yield (Asch and Wopereis 2001; Abdullah et al. 2001; Zeng et al. 2002; Kiani et al. 2006; Motamed et al. 2008; Rao et al. 2008; Clermont-Dauphin et al. 2010; Singh et al. 2010; Mojakkir et al. 2015; Raghavendra et al. 2018) are particularly influenced by the number of panicles (Asch and Wopereis 2001) and panicle length (Abdullah et al. 2001; Motamed et al. 2008; Mojakkir et al. 2015). Within-panicle characteristics affected by salinity include:

- Spikelet number per panicle regardless of season and development stage (Asch and Wopereis 2001),
- Number of primary (Abdullah et al. 2001; Motamed et al. 2008; Mojakkir et al. 2015) and secondary branches per panicle (Mojakkir et al. 2015),
- Number of grains per panicle (Abdullah et al. 2001; Motamed et al. 2008; Mojakkir et al. 2015),
- Number of filled grains per panicle (Abdullah et al. 2001; Motamed et al. 2008; Mojakkir et al. 2015),
- Grain weight per panicle (Abdullah et al. 2001; Motamed et al. 2008; Mojakkir et al. 2015),
- 1000 grain weight (Asch and Wopereis 2001; Abdullah et al. 2001; Motamed et al. 2008; Clermont-Dauphin et al. 2010; Mojakkir et al. 2015),
- Increased spikelet sterility (Asch and Wopereis 2001; Clermont-Dauphin et al. 2010) and
- Increased unfilled spikelets per panicle (Abdullah et al. 2001; Motamed et al. 2008; Mojakkir et al. 2015).

There is a strong relationship between salt tolerance at the reproductive stage and grain yield as maintaining a high number of fertile florets contributes to high seed set and thus grain yields as seen in tolerant genotypes. Contrastingly, higher spikelet sterility leads to poor seed set and lower grain yields in sensitive genotypes, due to significantly higher uptake of Na^+^ by anthers of sensitive compared to tolerant genotypes. For example, in sensitive IR64, Na^+^ was 21 mmol/g dry weight (dwt) in the anthers, in comparison with the more tolerant Cheriviruppu where Na^+^ was just 0.35 mmol/g dwt (Sarhadi et al. 2012). The Na^+^/K^+^ ratio in the anthers of IR64 under salt stress was more than 1.7 times higher than in plants grown under normal conditions, but in the tolerant genotype, Cheriviruppu, no significant change was observed for the Na^+^/K^+^ ratio. Since there was no significant change in K^+^ concentration in the anthers of either IR64 or Cheriviruppu under stress, the increase in the Na^+^/K^+^ ratio could clearly be attributed to an increase in Na^+^ uptake in IR64 under stress (Sarhadi et al. 2012). The presence of Na^+^ reduces pollen fertility, an important parameter for salinity tolerance at the reproductive stage and a direct determinant of yield. Although pollen fertility has been commonly accepted as a reliable phenotyping method, it is quite cumbersome and time-consuming to determine (Sarhadi et al. 2012). In addition to pollen fertility, stigmatic receptivity, also cumbersome to assess, is related to percent seed set; this was reduced by 38%, 75% and 100% when female plants of IR36 were grown in 10, 25 and 50 mM Na^+^ concentrations, respectively (Khatun and Flowers 1995).

While tolerance at the vegetative stage increases biomass for later stages, there is a poor association between seedling and reproductive stage salinity tolerance and it has been reported that there are different QTLs/genes independently controlling the tolerance at the two different stages (Moradi and Ismail 2007; Singh and Flowers 2010; Mohammadi et al. 2014).

### Phenotyping for salt stress at different stages

Accuracy in phenotyping is very important, and at the reproductive stage, a precise treatment at a specific growth stage is the key for phenotypic repeatability. The results of screening depend on the ambient conditions, particularly temperature and relative humidity, which play a vital role under salinity. Under controlled conditions (29 °C / 21 °C D/N at 70% RH), 50–120 mM NaCl is adequate to discriminate tolerant and sensitive genotypes of rice at the seedling stage, and 30–100 mM for the reproductive stage. However, under high temperature and low RH (34 °C / 25 °C D/N at 50% RH), the rate of transpiration increases, thus carrying more salt into the plant tissues and ultimately leading to severe injury or death (Singh et al. 2005). Hence, knowledge of ambient conditions, an optimum level of stress to use and the correct stage of crop growth along with standard tolerant and sensitive checks are vital in phenotyping for salinity tolerance. Most of the required conditions can be achieved in a controlled environment, unlike field-based screening techniques. However, comparing the results from the controlled conditions with those from field conditions is important in the final selection of desirable plants with a high level of tolerance (Kranto et al. 2016).

### Seedling and early vegetative stage

Phenotyping protocols for screening at the seedling and early vegetative stage are very well standardized and repeatable. Screening is mostly based on morphological parameters and relatively easy to achieve. Hydroponics is the best culture method available and ensures a uniform stress with ample nutrients, so that genotypic differences can be attributed to inherent differences of tolerance. The Yoshida culture-based method proposed by Gregorio et al. (1997) has been extensively used as a rapid method for screening large number of genotypes/populations. To counter the adverse effects of Na^+^ on other nutrients in Yoshida culture solution (Yoshida et al. 1976), a modified Yoshida solution was devised (modified by making the minor nutrients in neutral rather than acid solution, thus avoiding high concentrations of Na^+^, K^+^ or NH_4_^+^ required to adjust the pH; see Flowers and Yeo 1981) and is considered as the most appropriate for rice growth (Singh et al. 2010). While there are many variants in the way to screen in hydroponics, at IRRI, the use of perforated Styrofoam sealed with net, worked well as the Styrofoam platforms float on culture solution. Four-day-old, germinated seeds are grown on floats for three more days on nutrient solution under stress (usually NaCl of 100–120 mM, i.e., 10–12 dS m^−1^) before scoring. To validate the screening, every tray must include a tolerant genotype (like IR63307-3R-178-1-1 also known as FL 478) and a sensitive check (e.g., IRRI 154 or IR29). The scoring for seedling injury (Standard Evaluation System or SES score; Table 3) is recorded after 2 weeks based on the damage to the entry (see IRRI 2013; Singh et al. 2010).

### Reproductive stage

There is a good correlation between reproductive stage salinity tolerance and grain yield, but not always with seedling stage tolerance (Singh et al. 2004; Moradi and Ismail 2007; Singh and Flowers 2010). The best example is genotype FL478 which is used as a highly tolerant check for the seedling stage salinity screening. In studies at IRRI, FL478 shows a very high degree of sterility under salinity stress during its reproductive stage (Ahmadizadeh et al. 2016). Contrastingly, Sadri, an Iranian genotype, is very sensitive to salinity at the seedling stage but moderately tolerant at the reproductive stage (Mohammadi et al. 2014).

#### A novel phenotyping methodology for reproductive stage salinity

The very poor correlation between tolerance at the seedling and reproductive stages in some genotypes suggests that tolerance at these two stages is controlled by a different set of genes (Moradi et al. 2003; Moradi and Ismail 2007; Singh et al. 2008; Singh and Flowers 2010; Mohammadi et al. 2014). Of late, the importance of addressing the reproductive stage tolerance has been realized as it ultimately determines grain yield (Hossain et al. 2015). However, progress in phenotyping has been slow due to time-consuming and laborious protocols for the reproductive stage screening as compared with the relatively easy phenotyping protocols for the seedling stage (Jena and Mackill 2008; Calapit-Palao 2010). Screening for reproductive-stage tolerance in micro-plots filled with soil irrigated with saline water or soil preparations in pots or in natural field conditions have been proposed (Mishra 1996; Singh and Mishra 2004; Singh et al. 2008). However, under field conditions, controlling spatial variability in the soil and the imposition of uniform stress to a population consisting of genotypes with different phenology has proved difficult (Hossain 2014; Ahmadizadeh et al. 2016). Since the development of a precise and accurate phenotyping approach for the reproductive stage is both critical and very challenging, a technique has been devised at IRRI to salinize an individual genotype at the appearance of the flag leaf, which is about 1 week before the most sensitive gametophytic stage and pollen formation. In this way, each genotype can grow normally without any stress until the start of the most sensitive reproductive stage, irrespective of their growth duration.

The method developed at IRRI is based on the fact that in rice plants older leaves act as sinks where Na^+^ is accumulated so there is a cascade of loading from lower to upper leaves (Yeo and Flowers 1982) and ultimately the flag leaf, whose contribution to grain development is greater than other leaves (see Box 1). The method developed at IRRI (see Box 1 and Box 2) addresses the two major challenges for reproductive stage screening: (1) imposition of salinity stress exactly at the reproductive stage without stressing the plants at the seedling or late vegetative stages, and (2) imposition of stress on different genotypes or mapping populations at the same stage of development—the appearance of the flag leaf (Calapit-Palao et al. 2013; Ahmadizadeh et al. 2016). In this method, salt translocation at the reproductive stage is accelerated by the pruning of old leaves so salt moves faster to the developing panicle than in control plants without pruning (Box 1; Table 3). The protocol, which is described in Box 2 and involves screening at 10–12 dS m^−1^, has enabled genotypes displaying clear differences in tolerance at the reproductive stage to be identified (Table 4) and the independence of salinity tolerance at the seedling and reproductive stages to be established. For example, FL478, which is used as tolerant check (score 1–3) for seedling-stage-salinity screening, is sensitive (score 7) at the reproductive stage. Contrastingly, Sadri, an Iranian rice variety, is highly sensitive at the seedling stage (7–9) but moderately tolerant (5) at the reproductive stage (Mohammadi et al. 2014; Singh et al. 2021).

**Table:** 

**Fig. 1:**
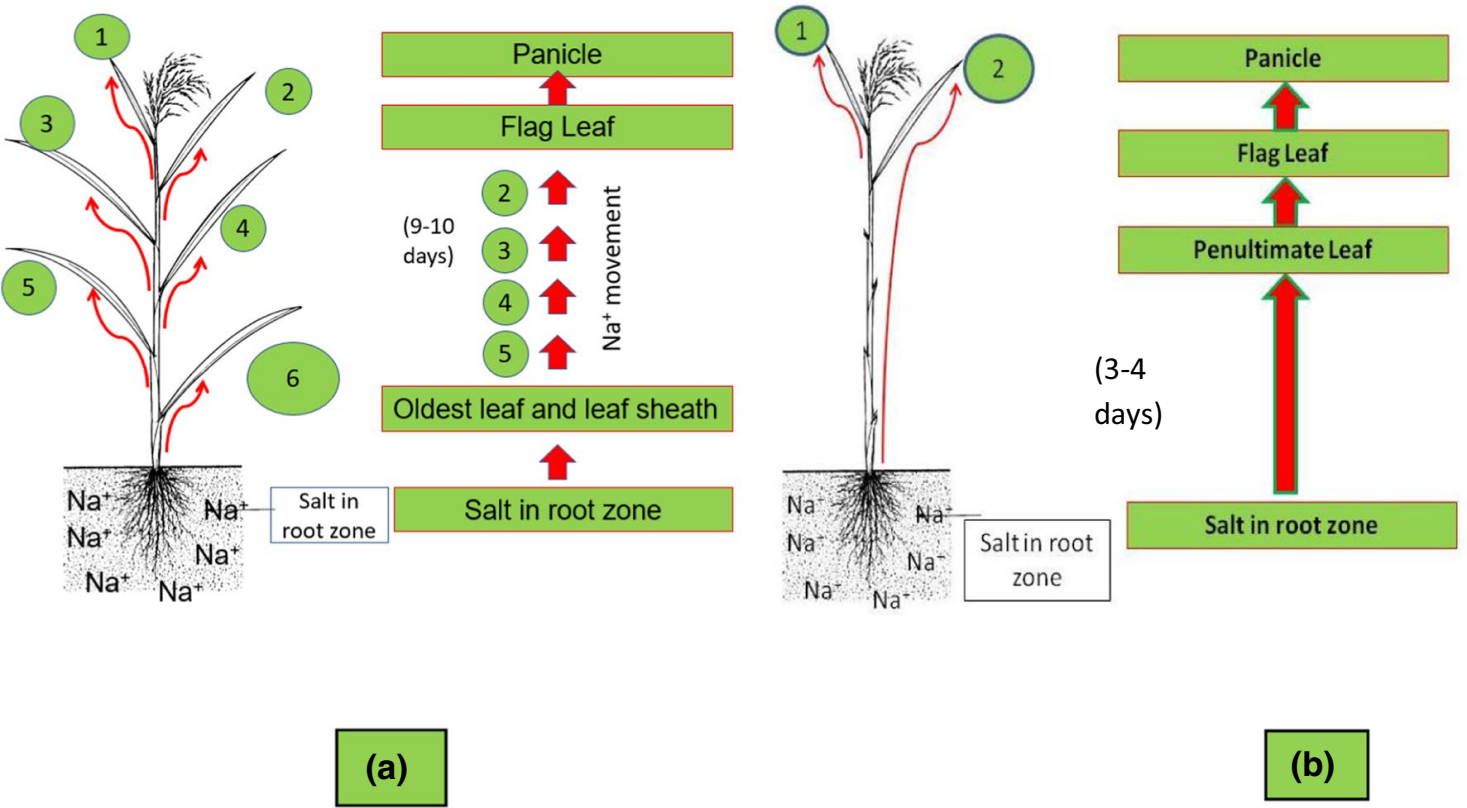
Mechanism of salt movement in rice and comparison of time taken by Na^+^ to reach the flag leaf after salinization with (**a**) no leaf pruning and (**b**) when only top two leaves are left. Red arrows indicate the movement of Na^+^ from the root zone towards the flag leaf

Box 1: Phenotyping protocol developed for screening for reproductive stage salinity tolerance
The phenotyping technique was developed based on two small sub-experiments
**Experiment 1**
In the first experiment, the yield data for control (no leaf blade pruning) were compared with (1) rice plants where only the flag leaf remained, (2) plants where the flag leaf and penultimate leaf were left and (3) plants where the flag leaf and two preceding leaves remained. Salt translocation at the reproductive stage is accelerated by the pruning of old leaves immediately after the first appearance of the flag leaf so that the sink for toxic ion compartmentation in treatment 2 is limited to two leaves (flag leaf and penultimate leaf) and leaf sheaths. Consequently, salt moves faster to the developing panicle than in control plants without pruning. There is a question of yield reduction due to leaf clipping, but there are many studies that indicate that the top three leaves are those that make the major contribution to grain yield, with the flag leaf alone contributing 45–60% to grain yield (Enyi 1962; Yoshida 1981; Abou-khalifa et al. 2008) (Fig. 1). The result of a study from Calapit-Palao et al. (2013) indicated that there was only any significant effect of leaf pruning on yield when the only remaining leaf was the flag leaf (Table 3). **Experiment 2** The second experiment was to compare the time taken by Na^+^ to reach the flag leaf when all leaves were intact with those where leaves had been pruned (only two leaves left). It was observed that Na^+^ reached the flag leaf/reproductive organs in about 9–10 days after treatment, if all the leaves were intact. However, if only the two top leaves were left and the rest clipped, Na^+^ took only 3–4 days to reach the flag leaf/reproductive organs (Fig. 1a and b). These two experiments fine-tuned the phenotyping protocol for salinity tolerance at the reproductive stage and ensured the stress was applied to all genotypes at a precise growth stage (Ahmadizadeh et al. 2016).

**Table 3:** Average number of filled grains/plant and 100-grain weight (g) of rice varieties IR64 and IR4630-22-2-5-1-3 grown with different pruning regimes under non-saline conditions, from Calapit-Palao et al. (2013)

Trait	Variety	Control	Regime 1	Regime 2	Regime 3	LSD (5%)
Filled grains	IR64	451a	208b	428a	388a	62.8
	IR4630-22-2-5-1-3	349a	247b	325a	346a	60.7
100-grain weight(g)	IR64	2.23a	2.08a	2.15a	2.18a	0.16
	IR4630-22-2-5-1-3	2.19a	1.91b	2.24a	2.35a	0.27

Lowercase letters indicate grouping (a,b) based on Duncan Multiple Range Test (DMRT) for significant difference from control at *P* = 0.05

Control—untrimmed plant/no leaf cut

Regime 1—only the flag leaf was left in the plant

Regime 2—two leaves left (penultimate and flag leaves)

Regime 3—top three leaves left

**Table 4:** Standard Evaluation System (SES) scores for phenotyping for salinity tolerance at the reproductive Stage/maturity

Score	Category	Symptoms	Examples (genotypes)
1	Highly Tolerant	Normal growth, spikelet sterility at ≤ 5%	Cheriviruppu, CSR28, Hasawi, Pokkali (IRGC Acc. No. 28689)
3	Tolerant	Growth slightly stunted, spikelet sterility at > 5 to 20%	IR4630-22-2-5-1-3, BRRI dhan 47
5	Moderately Tolerant	Growth moderately stunted, ¼ of all leaves brown, panicles partially exerted, spikelet sterility at 21 to 40%	Sadri, CSR43
7	Sensitive	Growth severely stunted with about ½ of all leaves become brown, panicles poorly exerted, high sterility at 41% to 70%	FL478, IR 64
9	Highly Sensitive	Growth severely stunted with almost all the leaves becoming brown and affected, panicles not exerted, delayed heading or papery florets/chaffy panicle with very high sterility at > 70%	IRRI 154, IR29, Swarna

**Table:** 

Box 2: Screening rice for salt tolerance at the reproductive stage
**Protocol**
The method involves sowing pre-germinated seeds in perforated plastic pots filled with fertilized soil (50 N, 25P and 25 K mg kg^−1^ soil), which are kept in concrete tanks filled with water. Two plants per plot are allowed to grow initially, thinned later to one plant per pot. A water level of 3 cm below the soil surface of the perforated pots is maintained in the tanks. All plants are grown under control conditions (using harvested rain water; EC < 0.2 dS/m) until the flag leaf appears when salt stress is applied—at the same growth stage for all genotypes. At the first appearance of the flag-leaf, individual pots are transferred to saline conditions with EC 10 dS m^−1^ (ca.100 mM NaCl) and are maintained under these saline conditions for 15–20 days, depending upon the ambient conditions (temperature and humidity as well as the degree of tolerance of the donor). Plants grown under similar conditions without salinization serve as controls. Leaf pruning, done at the first appearance of the flag leaf, is used to accelerate salt accumulation in the flag leaf by clipping old leaf blades. Consequently, only the flag leaf and penultimate leaf are available for salt accumulation and translocation to the reproductive organs. This accelerates, after 2 or 3 days, the effects of stress treatment and its effect on yield components as compared to that of control plants where all leaves are left untrimmed. Subsequently, all plants are transferred back to non-saline conditions. Yield and yield components including plant height, tiller number, panicle number, panicle length and panicle characteristics are estimated on a single plant basis. Samples for Na^+^ and K^+^ analysis can be taken from the flag leaf (small sample from base, middle and top of the leaf) as an indication of the degree of damage within the plant
The genotypes evaluated at the reproductive stage following the protocol can be scored based on the SES scoring system using a 1–9 scale where 1–3 are considered tolerant and 7–9 as sensitive—mainly based on flowering behavior and spikelet sterility (Table 4). This technique has greatly increased the efficiency of screening for the reproductive stage salinity tolerance and could be used as the basis of reproductive stage-specific screening for salinity tolerance in rice

### QTL mapping for seedling stage tolerance

Research on mapping quantitative trait loci, QTL, for salt tolerance in rice has advanced significantly in the last two decades. Several molecular markers in the form of isozymes and DNA markers (such as RFLP, RAPD, SSR, AFLP, VNTRs, CAPS, RAD-Seq) were designed and employed in QTL mapping studies. These methods have been used for the improvement of salt tolerance, utilizing wild rice genetic resources (Quan et al. 2018). Several studies have been undertaken to identify QTLs that quantify indices for plant survival and development under normal *vis-a-vis* stress conditions (Table 5).

**Table 5:** Reported QTL studies in rice for salinity tolerance (details in Online Resource 1)

Trait governing QTL	QTL study/ reference
Na^+^ accumulation in roots & shoots; K^+^ absorption, Na^+^ absorption, Na^+^/K^+^ ratio	Claes et al. (1990), Gregorio (1997), Lang et al. (2001b), Koyama et al. (2001), Bonilla et al. (2002), Niones (2004), Lin et al. (2004), Ren et al. (2005), Yao et al. (2005), Sabouri and Sabouri (2008), Zang et al. (2008), Thomson et al. (2010), Ahmadi and Fotokian (2011), Javed et al. (2011), Zheng et al. (2015), Qiu et al. (2015), Gimhani et al. (2016), De Leon et al. (2016), Dhanaya ka et al. (2018), Puram et al. (2017), Puram et al (2018)
Seedling survival, root dry weight, seedling dry matter, shoot dry weight, fresh weight shoot, fresh weight root, total biomass, seedling root length, shoot length	Gregorio (1997), Lang et al. (2001b), Koyama et al. (2001), Takehisa et al. (2004), Lin et al. (2004), Yao et al. (2005), Sabouri and Sabouri (2008), Zang et al. (2008), Sabouri et al. (2009), Thomson et al. (2010), Javed et al. (2011), Wang et al. (2012), Ghomi et al. (2013), Qiu et al. (2015), Gimhani et al. (2016), De Leon et al. (2016), Wang et al. (2017), Rahman et al. (2017), Bizimina et al. (2017), Puram et al. (2017), Dhanayaka et al. (2018),
Seedling stage salt injury, SES score, SSI for spikelet fertility at high salt concentration	Yao et al. (2005), Lee et al. (2006), Ammar et al. (2007), Zang et al. (2008), Sabouri et al. (2009), Thomson et al. (2010), Alam et al. (2011), Islam et al. (2011), Pandit et al. (2010), Tian et al. (2011), Javed et al. (2011), Ghomi et al. (2013), Zheng et al. (2015), De Leon et al. (2016), Wang et al.(2017), Rahman et al. (2017), Bizimana et al. (2017), Dahanayaka et al. (2017)
Leaf bronzing	Takehisa et al. (2006)
Plant stand, chlorophyll content, green leaf area	Sabouri and Sabouri (2008), Thomson et al. (2010), Ghomi et al. (2013), De Leon et al. (2016), Puram et al.(2017), Puram et al.(2018)
Relative germination energy, relative germination range, relative seedling height, relative root length, relative root number, relative vigor index, alkali damage rate in germination period, alkali damage rate at early seedling stage	Cheng et al. (2008), Tian et al. (2011), Puram et al. (2018)
Reduction of dry weight, reduction of fresh weight, reduction of leaf area, reduction of seedling height	Kim et al. (2009)
Na^+^ in leaves at reproductive stage, Cl^−^ in leaf at reproductive stage, Na^+^/K^+^ ratio in leaf at reproductive stage, K^+^ in leaves at reproductive stage	Ammar et al. (2009), Pandit et al. (2010)
K^+^ concentration in leaves at vegetative stage, Na^+^ concentration in straw at high salt stress, Na^+^/K^+^ ratio in straw at high-salinity stress, Cl^−^ ion concentration in leaves at vegetative stage, Na^+^ concentration in stem at vegetative stage	Ammar et al. (2009), Pandit et al. (2010), Fayed and Farid (2017)
Plant height, tiller number, panicle length, number of fertile spikelets, grain yield per plant, spikelet fertility, number of sterile spikelets, days to flowering, number of panicles, straw dry weight, number of fertile spikelets, total spikelets number, 1000-grain weight	Mohammadi et al. (2013), Zang et al. (2008), Gregorio (1997), Takehisa et al. (2004), Sabouri and Sabouri (2008), Thomson et al. (2010), Calapit-Palao et al. (2015), Hossain et al. (2015)
Germination rate, imbibition radicle length, coleoptile fresh weight, coleoptile dry weight, plumule fresh weight, radicle dry weight, germination percentage, radicle fresh weight, plumule dry weight, plumule length	Mardani et al. (2014), Wang et al. (2011)
Shoot dry weight at maturity, % potassium (K), pollen fertility, % Sodium (Na), Na^+^/K^+^ ratio, panicle length, root dry weight, single-grain weight, sodium concentration at reproductive stage, Na^+^/K^+^ ratio at reproductive stage	Calapit-Palao et al. (2015), Hossain et al. (2015)

Initial molecular studies were based on characterization and expression of salinity-induced tissue-specific proteins (e.g., Claes et al. 1990). Later, genetic studies based on populations derived from diverse parents differing for salt tolerance were utilized to locate the genomic regions associated with salt tolerance. With the development of an RFLP-based linkage map of rice (based on an F2 derived from *O. sativa* and *O. longistaminata*), the *salT* gene was linked to the RFLP marker RG 146B, localized on chromosome 1. This was the first gene reported to be associated with salinity tolerance (Causse et al. 1994). Later, genetic studies, mostly through biparental mapping populations, identified multiple genes/loci associated with salinity tolerance in rice along with their chromosomal locations and these findings helped in improvement of the trait (Gregorio and Senadhira 1993; Causse et al. 1994; Zhang et al. 1995; Ding et al. 1999; Quan et al. 2018). Numbers of QTLs were identified across the different chromosomes associated with seedling stage salinity tolerance (Gregorio et al. 1997; Ren et al. 2005; Thomson et al. 2010).

### *Saltol* QTL and other genomic regions in seedling stage salinity tolerance

*Saltol*, a major QTL governing salinity tolerance, was mapped in F8 RILs of a cross between IR29 (salt sensitive) and Pokkali (salt tolerant) at the International Rice Research Institute (Gregorio 1997). The genomic region where this QTL was located contains a major gene found to possess three common QTLs for maintaining low Na^+^ uptake, high K^+^ uptake and Na^+^ /K^+^ homeostasis in shoots with 64.3–80.2% of total phenotypic variation (PV) conferring seedling stage salinity tolerance. Later, the *Saltol* region was precisely localized (Bonilla et al. 2002). Niones (2004) fine-mapped the common QTL region of *Saltol* in BC3F4 near isogenic lines (NILs) of IR 29/Pokkali. In addition to this major QTL (*Saltol*), 7 QTLs including three for Na^+^ uptake, two for K^+^ uptake and two for Na^+^ /K^+^ ratio were detected on chromosomes 3, 4, 10 and 12. One of the lines (IR 66946-3R-178-1-1, also known as FL478) was identified from a RIL population of the cross IR29/Pokkali that exhibited salt tolerance higher than or comparable to the tolerant parent, Pokkali. Using the same IR29/Pokkali-derived RIL population, Thomson et al. (2010) made a comprehensive study of the *Saltol* QTL and other major QTLs (other than *Saltol*) for shoot Na^+^ /K^+^ ratio, root K^+^ concentration, root Na^+^ /K^+^ ratio, seedling height, leaf chlorophyll content, initial SES tolerance score, final SES tolerance score and seedling survival across chromosomes 2, 3, 4, 6, 9 and 12. They (Thomson et al. 2010) found multiple Pokkali alleles introgressed into different RILs at different chromosomal regions including alleles at the *Saltol* locus, which is similar to that of QTL *SKC 1* characterized from another highly salt-tolerant land race, Nona Bokra (Ren et al. 2005).

*Saltol* is a major QTL for salinity tolerance at the seedling stage; however, the contribution of *Saltol* to visual leaf injury at the seedling stage, as measured through the IRRI SES score (IRRI 2013), is not sufficient to provide a high degree of salt tolerance. Nine NILs, each with a single Pokkali introgression at the *Saltol* QTL, were evaluated in a saline field (stress conditions) in Iloilo, Philippines, and under controlled conditions (non-stress) at IRRI. The results showed that only two NILs exhibited a superior performance over the sensitive parent IR29 suggesting the need for combining seedling and reproductive stage tolerance while introgressing salinity tolerance into elite lines to address any yield penalty due to salt stress (Thomson et al. 2010).

Simultaneously, several research groups have explored different genetic resources to understand and dissect the genetic basis of salinity tolerance; this has led to identification of several QTLs spanning across the genome (Table 5; Online Resource 1). QTLs for percent seed germination, seedling root length, seedling dry matter and seedling vigor were reported (Prasad et al. 2000; Mardani et al. 2014). Several SSR and RFLP markers linked to QTL regions for shoot and root dry weight, Na^+^ and K^+^ absorption and Na^+^ /K^+^ ratio governing seedling salinity tolerance have also been reported (Lang et al. 2001a, b, 2008). Importantly, the net quantity of ions transported to shoots (Na^+^ uptake, K^+^ uptake and Na^+^ /K^+^ ratio) rather than their concentration is directly related to salinity tolerance with independent inheritance of Na^+^ and K^+^ uptake (with different pathways of apoplastic leakage and membrane transport respectively; Koyama et al. 2001). QTLs located away from *Saltol* or *Sal*T regions were detected (Koyama et al. 2001). Although several QTLs were mapped for various traits associated with seedling tolerance, very few of them are being utilized in breeding because of difficulties in transferability of QTLs for physiological traits to an unrelated genetic population (Flowers et al. 2000).

Root QTLs for the total quantity of Na^+^ in the root (*qRNTQ-1*) and root K^+^ concentration (*qRKC-4*) underpinning salt tolerance were first reported in the cross Nona Bokra/Koshihikari (Lin et al. 2004). QTLs for root and shoot were reported to be located on different linkage groups suggesting that genes controlling transport of Na^+^ and K^+^ between shoots and roots may be different or induced uncoordinatedly by salt stress (Lin et al. 2004). QTLs for salinity tolerance rating (STR), weight of shoot dry matter and Na^+^ /K^+^ ratio at the seedling stage were also reported by Yao et al. (2005). Ren et al. (2005)*,* fine-mapped qSKC-1, a major QTL localized within the *Saltol* locus, reported previously by Lin et al. (2004). The *SKC1* gene (*Os01g20160*) controlling K^+^ /Na^+^ homeostasis encodes an OsHKT-type Na^+^ selective transporter and is preferentially expressed in parenchyma cells surrounding the xylem vessels. Thus, *SKC1* affects K^+^ and Na^+^ translocation between roots and shoots and thereby regulates K^+^ /Na^+^ balance in the shoots. There are numerous studies on seedling stage salinity tolerance attributed to different underpinning traits spanning almost all the chromosomes of cultivated rice (Table 5). There are a few studies on salinity tolerance from wild rice accessions of *Oryza rufipogon*-derived introgression lines (ILs) associated with salt tolerance score (STS), relative root dry weight (RRW), relative shoot dry weight (RSW) and relative total dry weight (RTW), which identified a total of 15 QTLs for four traits (Tian et al. 2011). The detailed information on all the major reported QTLs for salinity tolerance is summarized in Table 5 and detailed in Online Resource 1.

### Beyond seedling stage salinity tolerance; QTLs for reproductive stage tolerance of rice

Very few studies have attempted to dissect the genetic basis of tolerance at different growth stages, especially at tillering and flowering. Identification of main-effect QTLs governing salt tolerance at different growth stages will enable an understanding of the genetic nature of salt tolerance and hasten breeding for salt tolerance by facilitating pyramiding of component QTLs using molecular technologies. Takehisa et al. (2004) evaluated backcross inbred lines (BILs) derived from backcrossing Nipponbare/Kasalath//Nipponbare in BC_1_F_9_ to BC_1_F_12_ generations for four cropping seasons in a paddy field flooded with saline water and a separate non-saline paddy and detected 17 QTLs. Two QTLs for leaf bronzing with epistatic effects were later detected (Takehisa et al. 2006). Ammar et al. (2009) reported 25 QTLs for 17 traits including seedling-salt-injury score, Na^+^, K^+^, Cl^–^ concentrations and Na^+^ /K^+^ ratio in leaf and stem at vegetative and reproductive stages using F2:F3 population derived from CSR27/MI48. Mohammadi et al. (2013) studied salinity tolerance at the reproductive stage and identified 35 QTLs for 11 traits, of which most were found to be novel for reproductive stage tolerance. However, the major issue in these studies was that the salinity treatment was given at the same time to all the genotypes irrespective of their growth stage at that time (reproductive stage or not).

The first report of QTL mapping for reproductive stage salinity tolerance in rice based on reproductive stage-specific phenotyping with selection pressure exerted exclusively at the time of flag leaf appearance (cf. Box 2) was carried out in a population derived from Cheriviruppu and Pusa Basmati 1 (Hossain et al. 2015). They (Hossain et al. 2015) identified 16 QTLs with LOD values ranging from 3.2 to 22.3 on chromosomes 1, 7 and 8 with the maximum number of QTL clusters for different component traits co-localized on the long arm of chromosomes 1 and 7. Pollen fertility, Na^+^ concentration and Na^+^/K^+^ ratio in the flag leaf were found as the most important mechanisms controlling salt tolerance at the reproductive stage in rice. Calapit-Palao et al. (2015) also carried out phenotyping specifically for the reproductive stage and identified QTLs for reproductive stage salinity tolerance using F_2_ population of the cross IR64/IR4630-22-2-5-1-3 for yield components, pollen fertility and physiological parameters under salt stress imposed at flag leaf emergence. Three significant (RM455, RM223, and RM271) marker loci on chromosomes 7, 8 and 10 were found to be significantly associated with Na^+^/K^+^ ratio. Two significant markers RM11 and RM455 for percent Na^+^ and K^+^ were co-localized on chromosome 7 and were responsible for 7.7% to 10.2% of the phenotypic variation. A few more studies conducted for reproductive stage salinity tolerance are reported in Table 5 (details in Online Resource 1), but their phenotyping was not carried out specifically at the reproductive growth stage (Box 2). These results have not been pursued for fine mapping and to develop closely linked markers, perhaps because of the low reliability of such studies.

### Meta-QTL analysis

Combining results from multiple studies allows greater statistical power for QTL detection and their potential use for genetics and breeding. We have carried out a meta-analysis of salinity tolerance QTLs to provide a reliable integration of information of multiple traits associated (MTA) and multiple QTLs located (MQL) in a single genomic region across various genetic backgrounds and various growth stages. The aim was to detect consistent QTLs that are promising for estimating the position of genes.

**Fig. 2:**
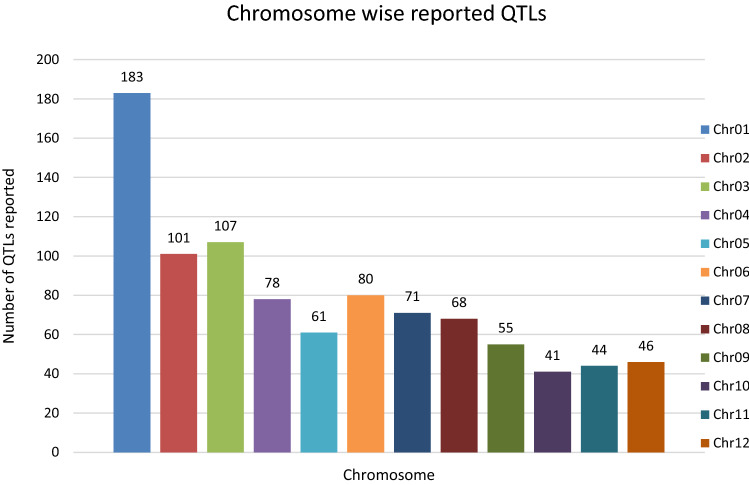
Chromosome locations of QTLs for salt tolerance in rice from mapping populations of different genetic backgrounds are distributed in all 12 chromosomes

In the past two decades, many QTLs have been reported for different growth stages of salinity tolerance (Table 5 and Online Resource 1), but very few have been cloned to date, just, *SKC1*, *qSE3* and OsHAK21 all related to K^+^ homeostasis (Ren et al. 2005; He et al. 2019). Among the reported 935 QTLs from 46 different QTL studies for salinity tolerance at both vegetative and reproductive stages in different genetic background of biparental mapping populations (detailed information including parents, type and size of mapping population and the reported QTLs are presented in Online Resource 1), only 567 QTLs (see below) were utilized in the meta-analysis (Fig. 2). The specific information on experimental conditions under which QTLs were detected, peak position and flanking markers of detected QTLs, logarithm of the odds (LOD) score, phenotypic variation explained by each QTL and the genetic map information of each study were collected from individual publications as well as from the Gramene database (http://gramene.org). Studies with missing information were excluded from the analysis. Chromosome-wise consensus genetic maps were developed for all chromosomes. The QTLs discovered were from 13 different genetic background mapping populations (BC_1_F_9_, BC_2_F_5_, BC_2_F_8_, BC_3_F_2_, BC_3_F_4_, BC_3_F_5_, BC_4_F_4_, BIL_s_, DH_s_, F_2_, F_2_:F_4_, IL_s_ and RIL_s_) with a majority of the QTLs identified from RILs. The distribution of projected QTLs in the meta-analysis showed that chromosome 1 has highest number of QTLs followed by chromosomes 3, 2, 6 and 4. Most of the QTL mapping studies were based on genetic linkage maps which do not provide the exact physical position of the reported QTLs.

**Fig. 3:**
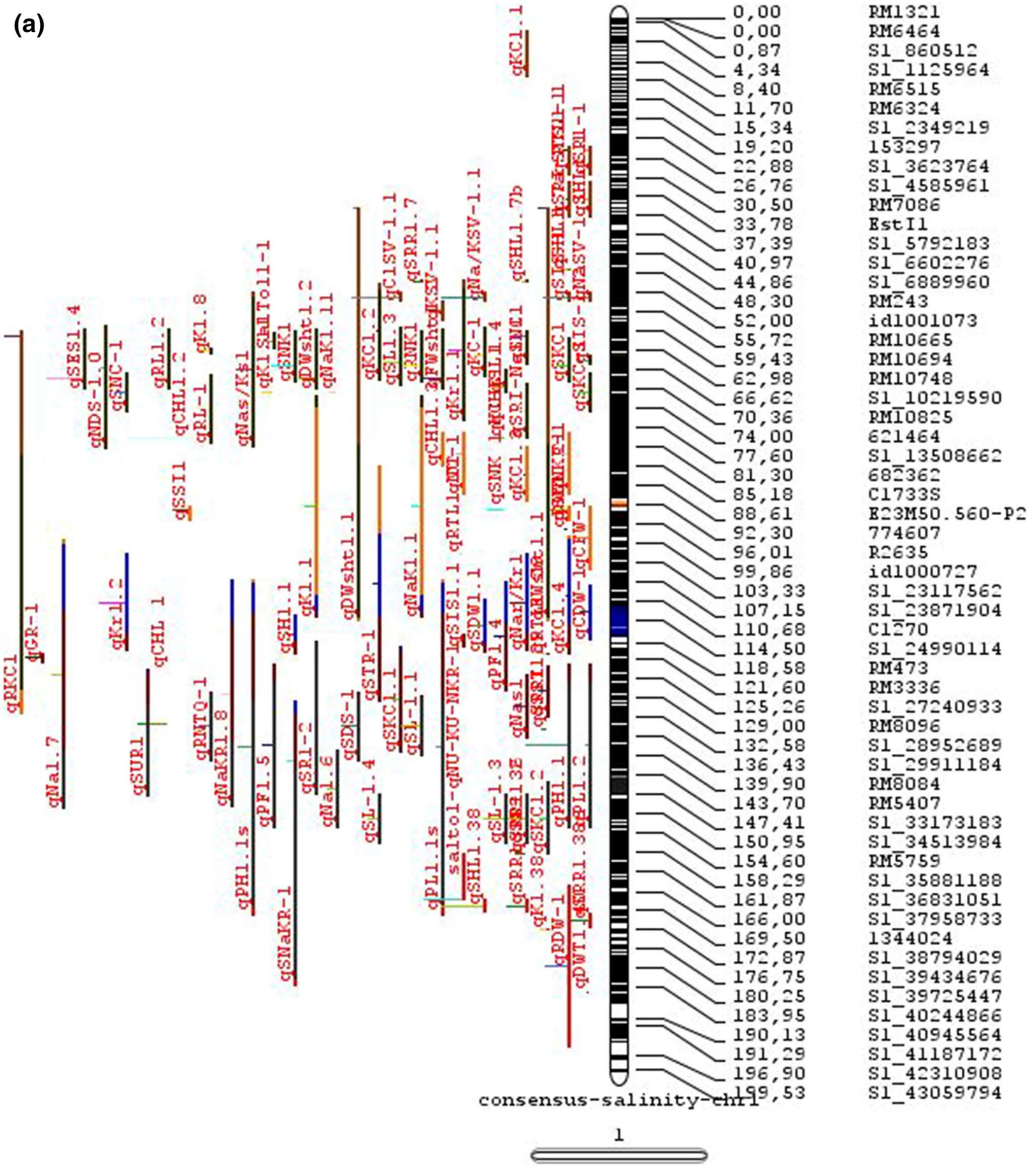

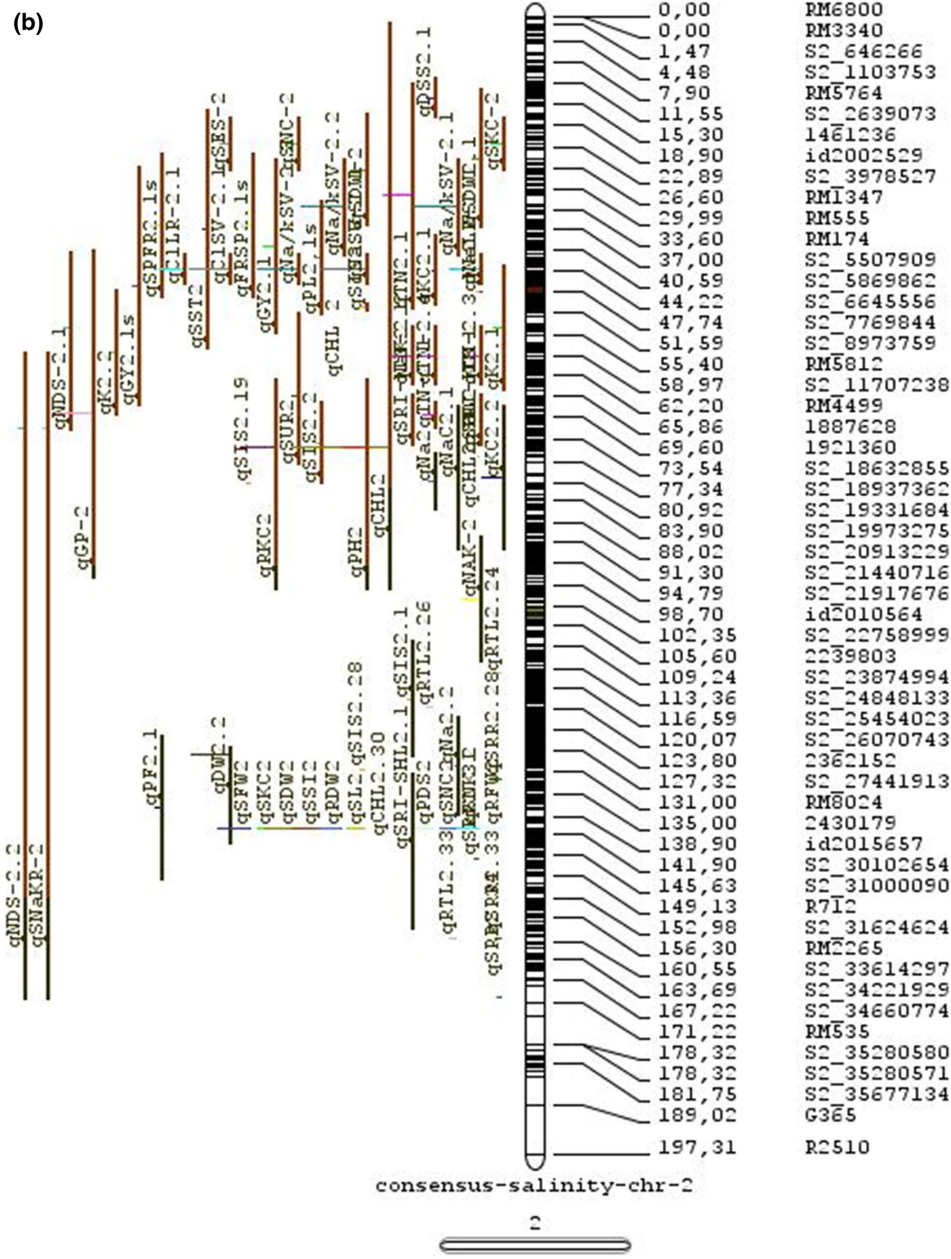

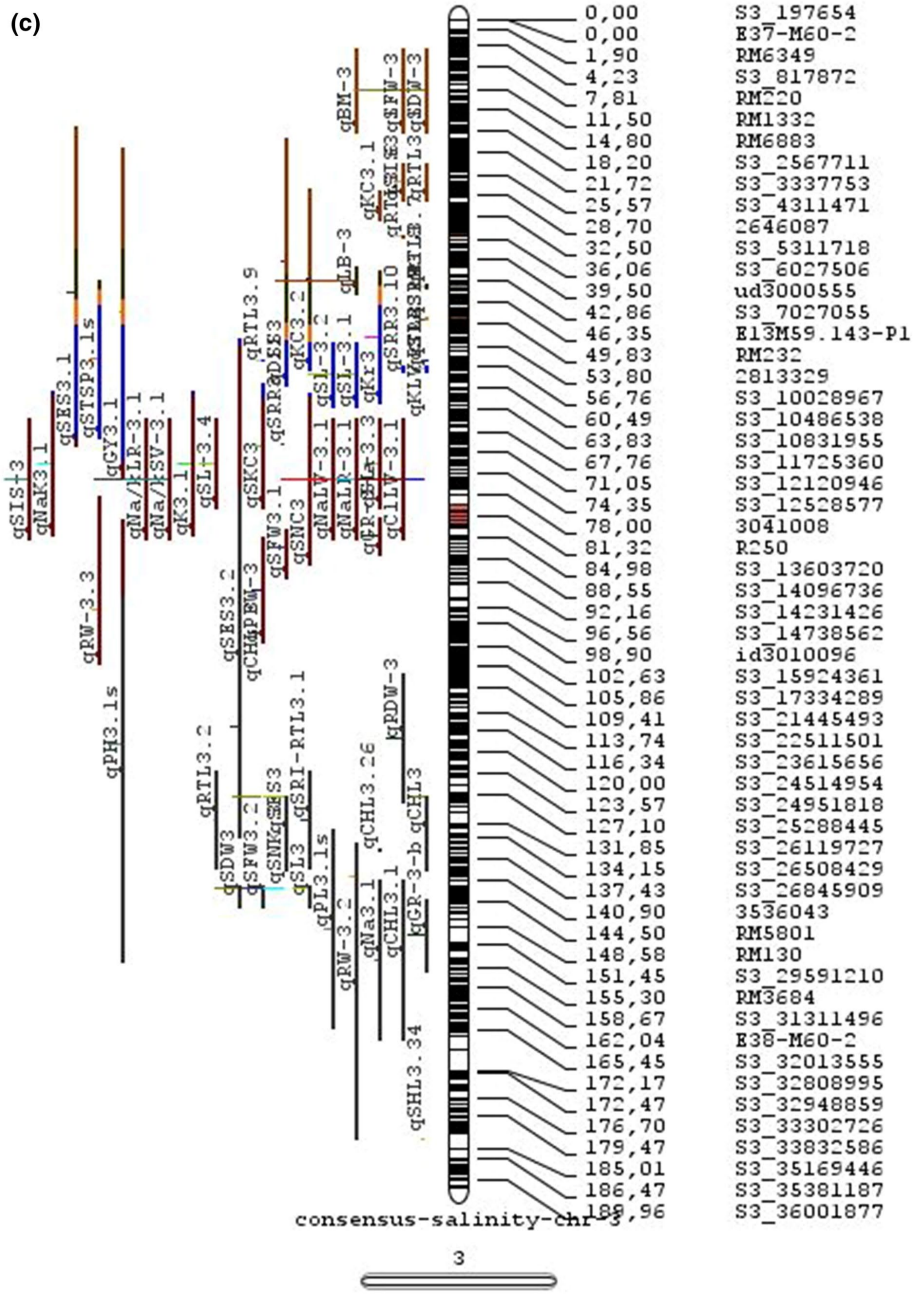

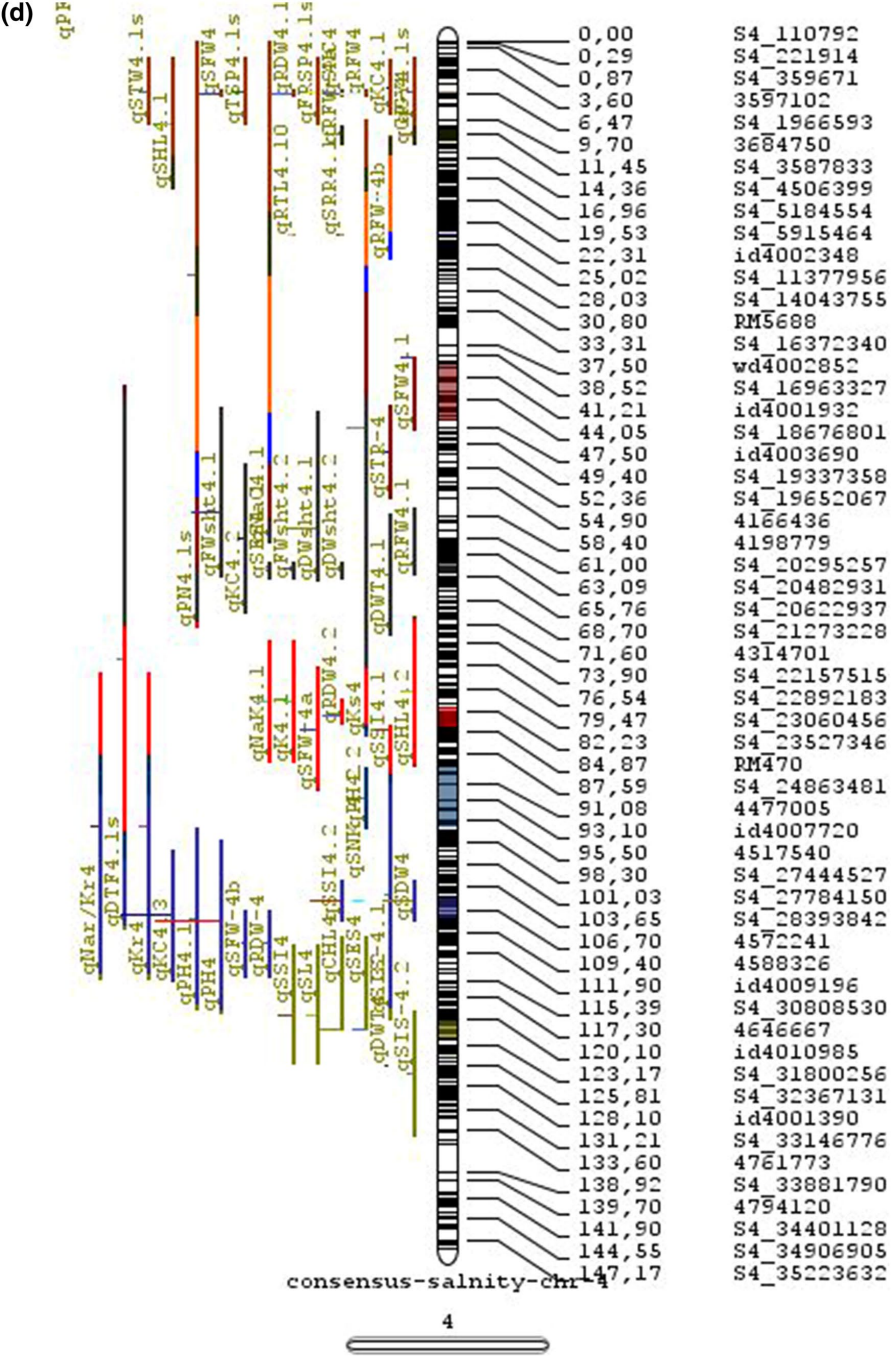

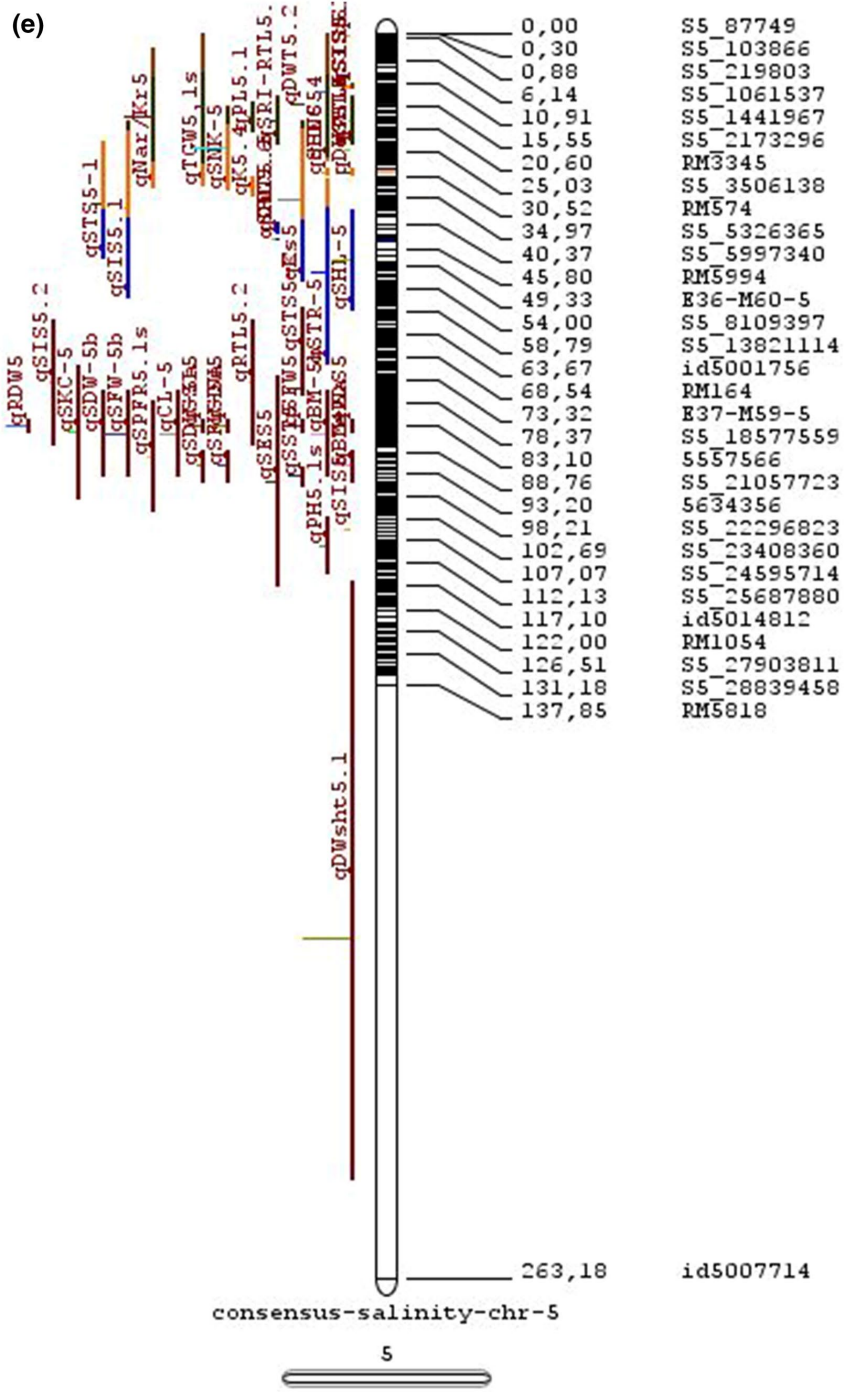

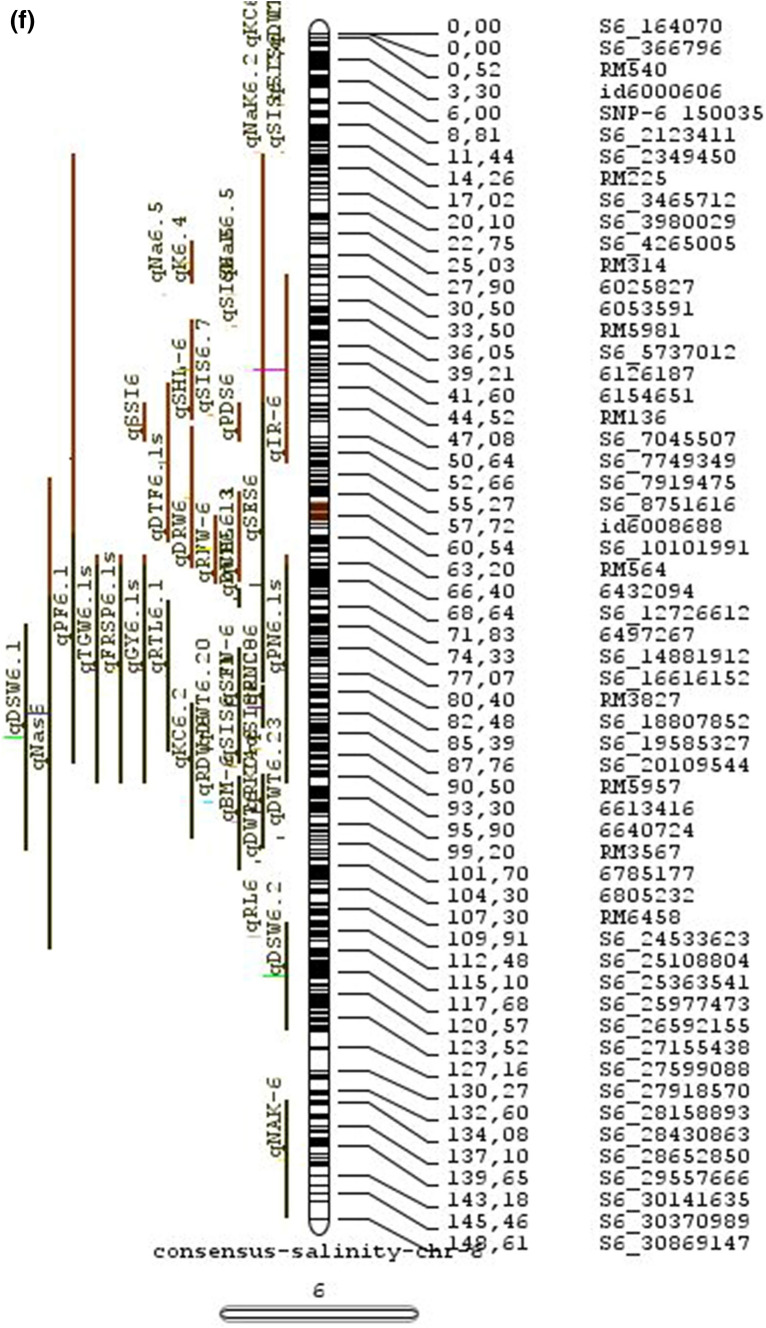

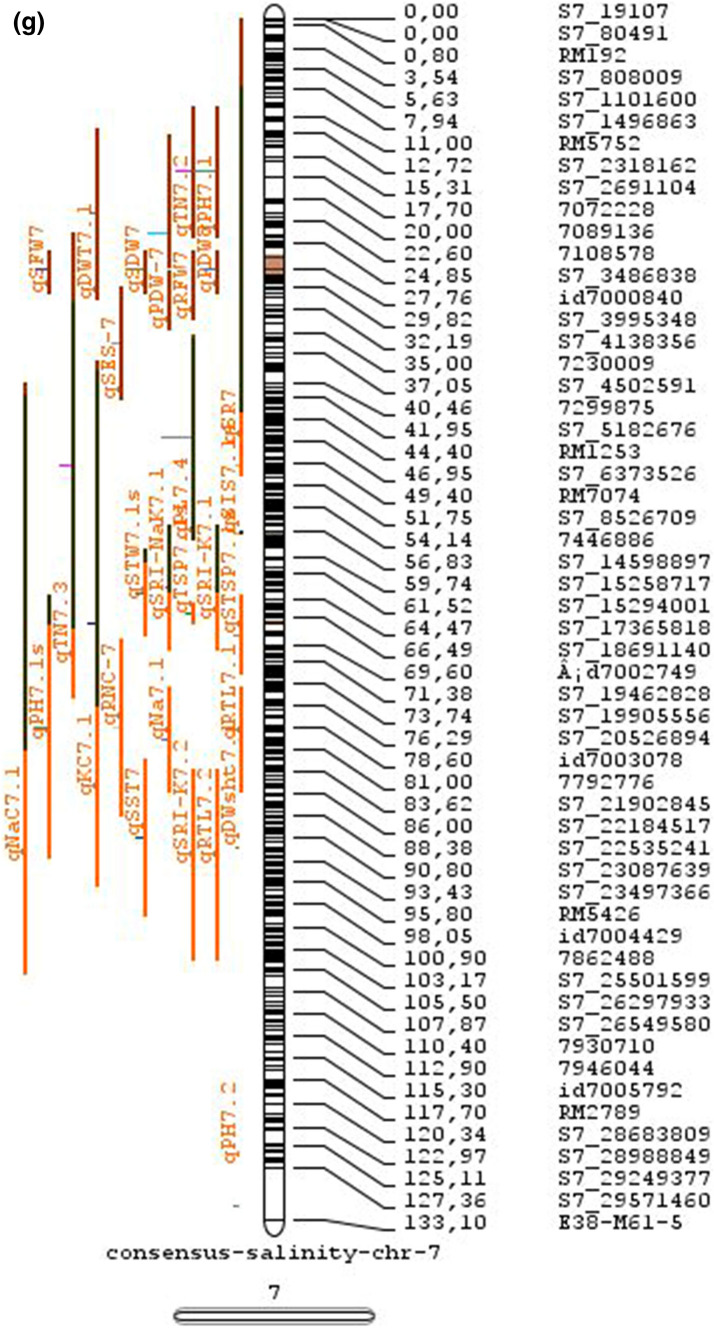

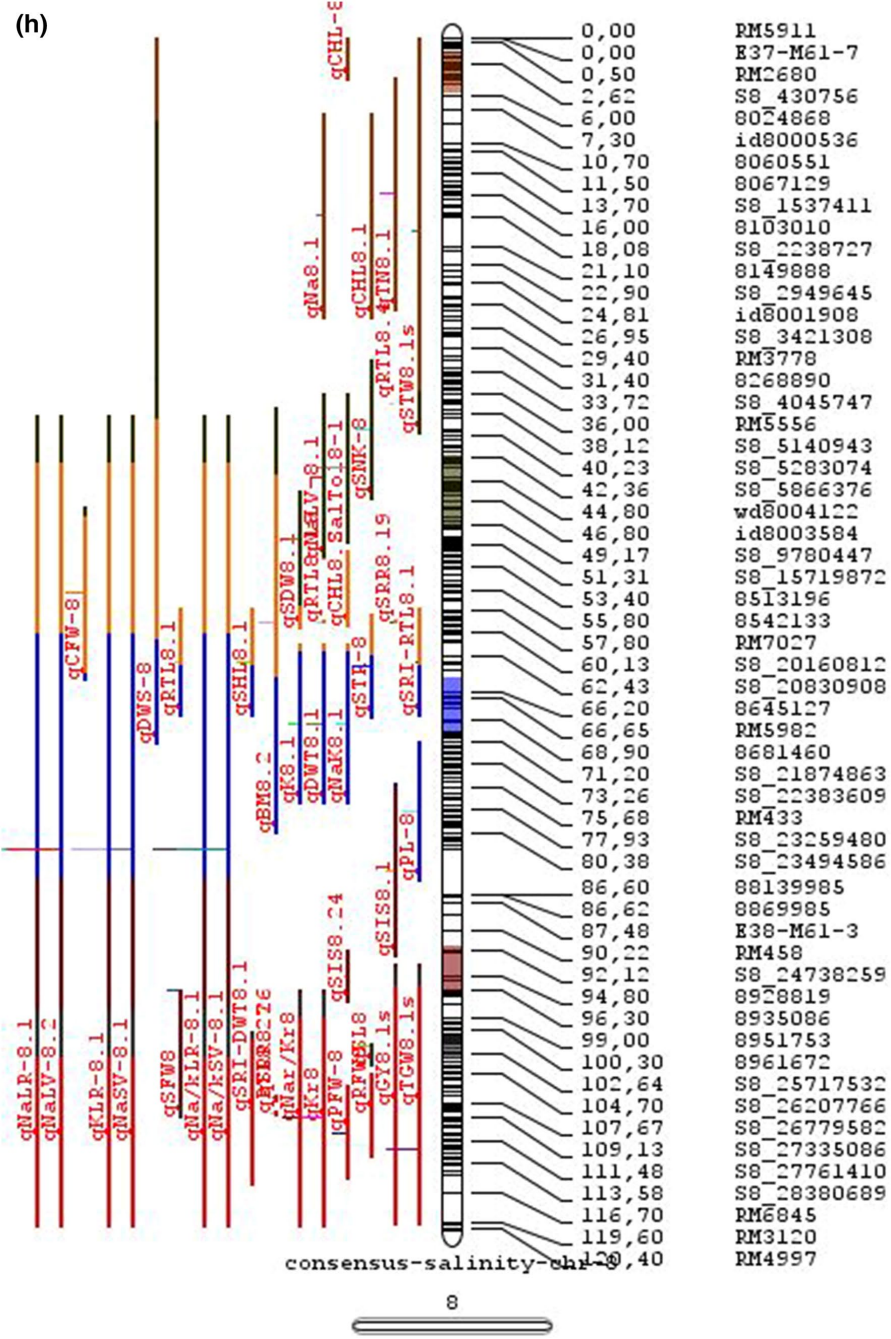

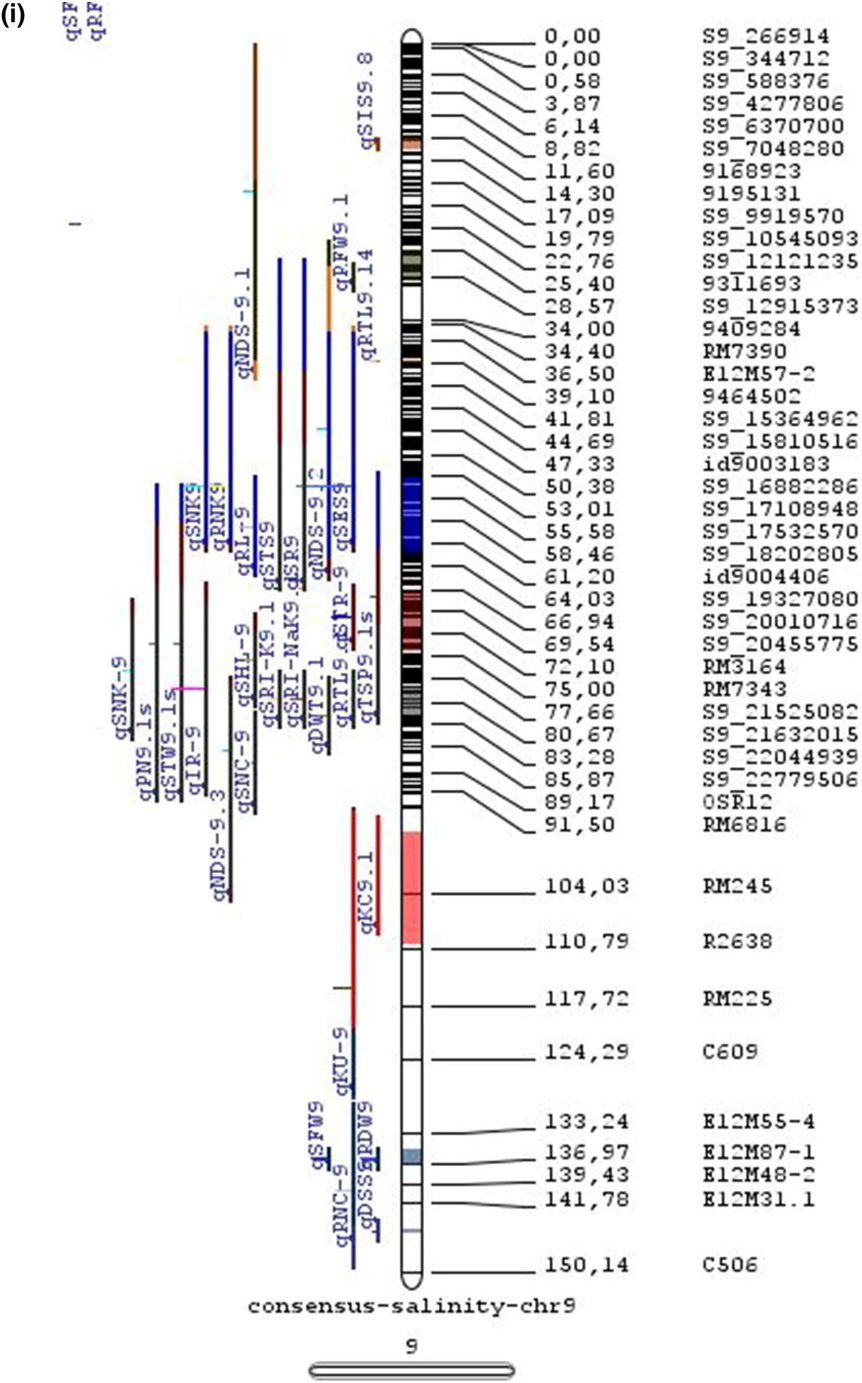

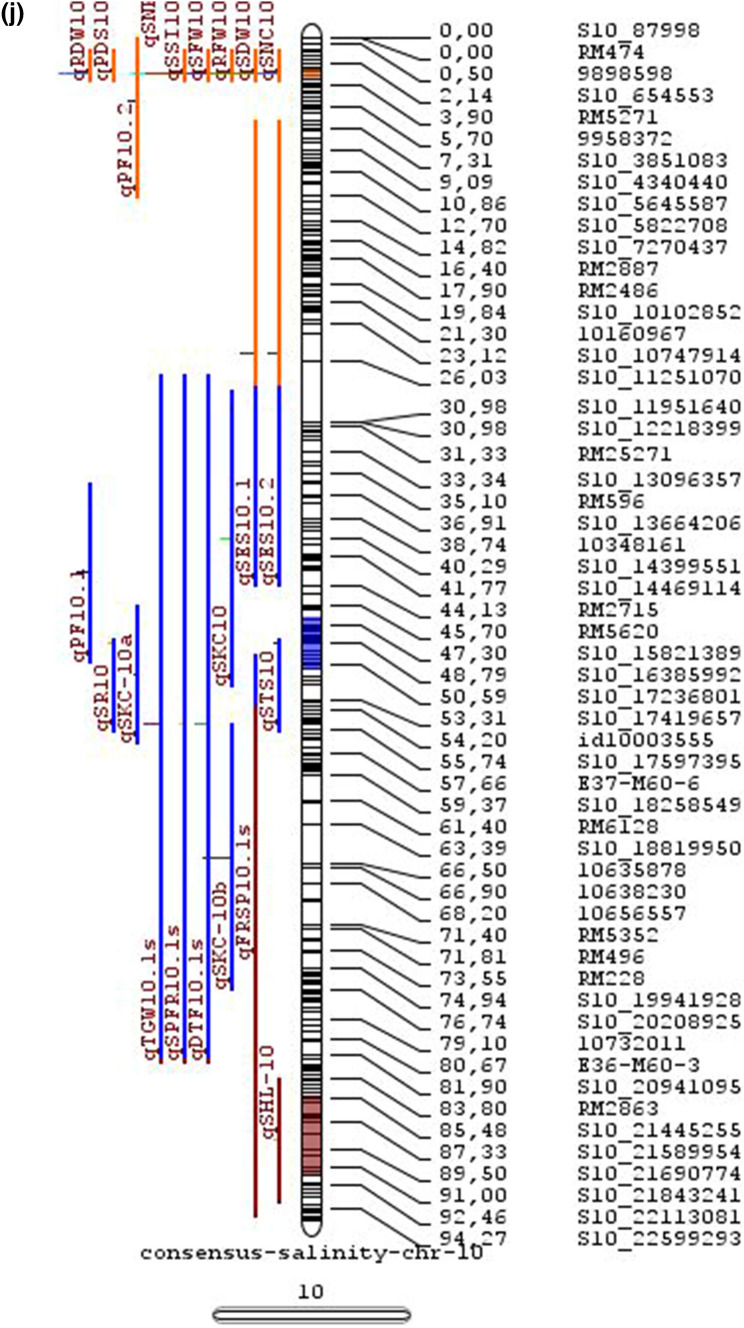

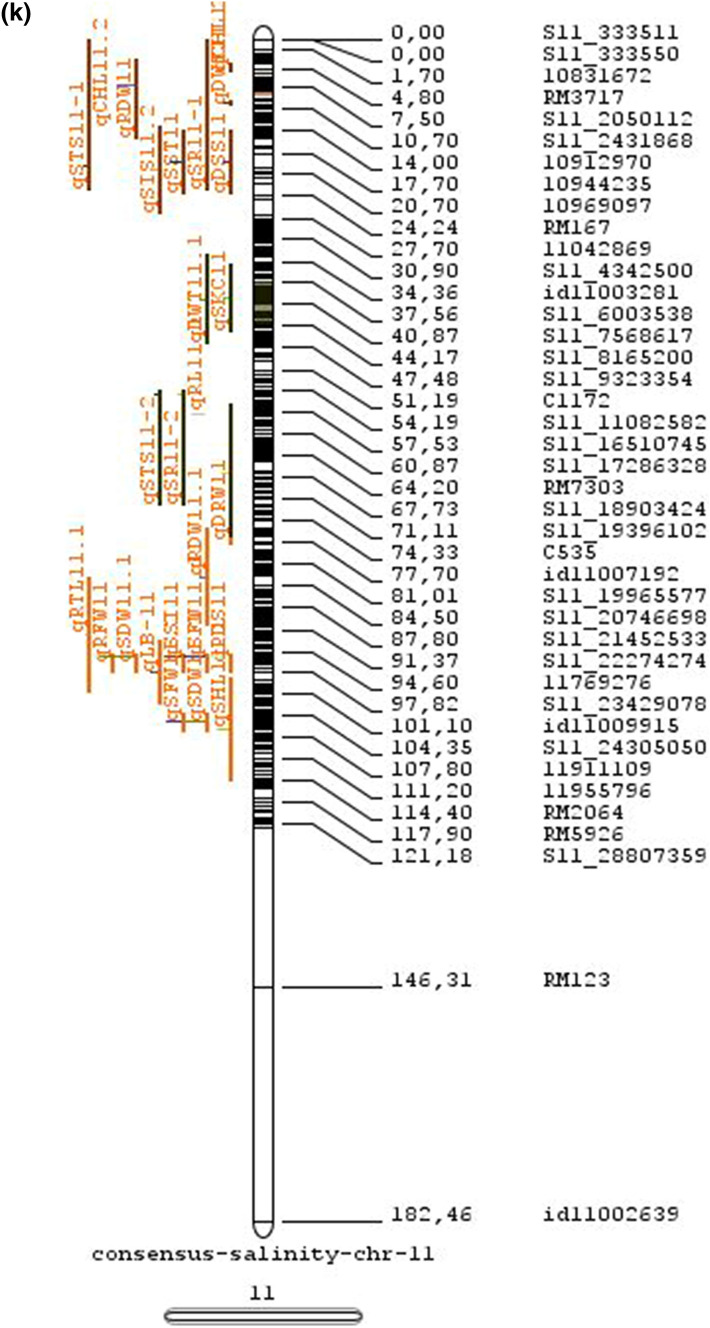

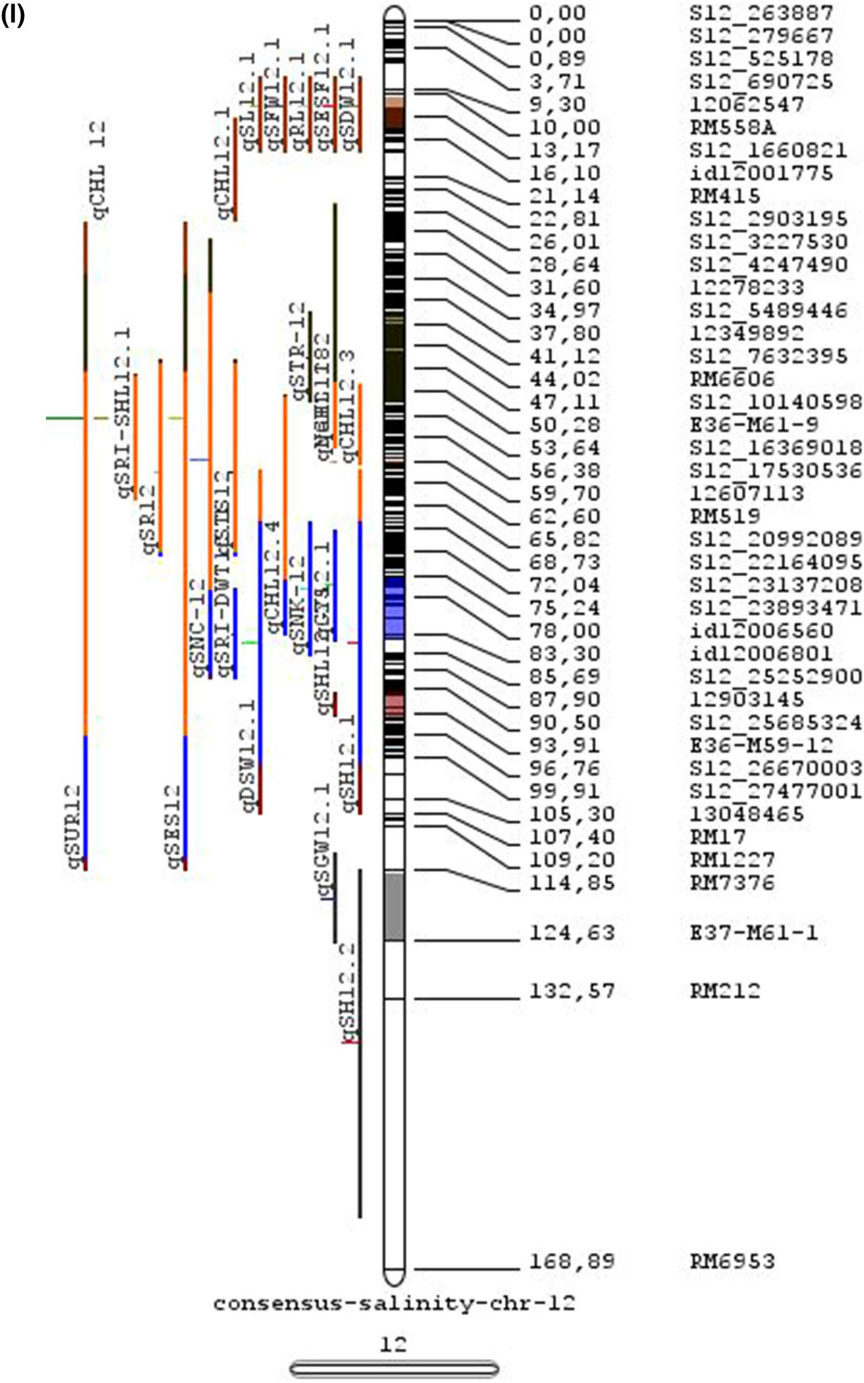
Chromosome-wise consensus meta-QTLs based on 567 QTLs from 46 studies for salinity-related traits

The meta-analysis reported by Islam et al. (2019) revealed 11 meta-QTLs for three salinity tolerance traits with small confidence intervals that were localized on chromosomes 1 and 2. Our meta-analysis indicated 24 candidate genes in 15 meta-QTLs that spanned physical intervals < 0.2 Mb, including genes that have been cloned previously (e.g., *EP3, LP, MIP1, HTD1, DSH1*, and *OsPNH1*; Wu et al. 2016). A total of 63 meta-QTLs with CI of 95% were identified from 567 QTLs detected from different studies projected for salinity traits (Table 6 and Fig. 3). The meta-QTLs indicate the most important genomic regions that have the highest probability of success if specifically targeted for the introgression of salt tolerance in breeding materials through marker-assisted selection. A number of studies have used a meta-QTL region to develop reliable flanking markers for introgressions. Among them, the successful programs include the introgression of *Saltol* within first mQTL1.1: mQTL1.1 and mQTL1.2 include genes responsible for salinity tolerance, like *OsCPK17, OsRMC, OsNHX1, OsHKT1;5* and *Sal*T. The roles of these five genes have been substantiated by Negrao et al. (2012) who reported their allelic variants and their haplotypes associated with salinity tolerance. Eleven out of 32 SNPs identified from four of five tested genes were found to be significantly associated with salt tolerance. *OsHKT1;5* (*LOC_Os01g20160*) for shoot K^+^ homeostasis was found to be the most diverse gene as evidenced from its 15 haplotypes in the germplasm based on 29 SNPs and two indels variants. NonaBokra, Koshihikari and Pokkali possessed the same haplotype, while other salt-tolerant genotypes like FL478, IR52724-2B-6-2B-1-1 or Hasawi exhibited different *OsHKT1;5* haplotypes, although all of them are highly salt-tolerant at the seedling stage.

**Table 6:** Meta-QTLs identified from the reported individual QTLs studies for seedling and reproductive stage salinity tolerance

S.No	Chromosome	MetaQTL	Position (cM)	CI (95%)	Flanking markers
1	1	mQTL 1.1	48.81	0.24	RM 577 -S1_7520107
2	1	mQTL 1.2	64.39	0.9	RM10745 -S1_9340031
3	1	mQTL 1.3	92.41	1.59	RM6880 -779469
4	1	mQTL 1.4	114.78	5.34	S1_24780749 -RM 486
5	1	mQTL 1.5	121.02	0.28	RM11542 -S1_26449563
6	1	mQTL 1.6	145.61	3.83	1163456 -RM3422
7	1	mQTL 1.7	172.71	0.02	RM8049 -S1_38794029
8	2	mQTL 2.1	47.93	1.25	S2_7478506 -S2_8096678
9	2	mQTL 2.2	103.85	2.6	RM 6107 -2236772
10	3	mQTL 3.1	35.42	0.59	id3004633 -S3_6027506
11	3	mQTL 3.2	43.87	0.84	S3_7066823 -S3_7209963
12	3	mQTL 3.3	49.29	0.6	RG369 -S3_8357070
13	3	mQTL 3.4	56.1	0.21	RM282 -S3_9891061
14	3	mQTL 3.5	81.25	3.61	id3009433 -3096758
15	3	mQTL 3.6	136.86	0.02	RM2593 -S3_26845909
16	4	mQTL 4.1	6.64	0.45	S4_1966593 -S4_2036989
17	4	mQTL 4.2	11.76	1.64	S4_3570866 -S4_3881858
18	4	mQTL 4.3	23.99	0.24	S4_10625625 -3883297
19	4	mQTL 4.4	23.99	0.4	S4_10625625 -S4_10841800
20	4	mQTL 4.5	43.47	7.1	id4003259 -id4003690
21	4	mQTL 4.6	64.87	1.32	S4_20523929 -S4_20622937
22	4	mQTL 4.7	83.2	2.68	S4_23278361 -S4_24125704
23	4	mQTL 4.8	92.96	7.49	4177005 -S4_27200682
24	4	mQTL 4.9	106.6	2.6	S4_28742183 -S4_29548991
25	4	mQTL 4.10	121.54	2.3	4665219 -S4_31772822
26	5	mQTL 5.1	11.76	0.27	S5_1545025 -S5_1671090
27	5	mQTL 5.2	15.3	0.59	S5_2111966 -S5_2173849
28	5	mQTL 5.3	29.95	1.21	S5_4565557 -S5_4699921
29	5	mQTL 5.4	43.84	0.56	5210158 -S5_644838
30	5	mQTL 5.5	85.18	0.09	S5_20342607 -S5_20461863
31	6	mQTL 6.1	60.23	2.57	¡d6008704 -S6_10384890
32	6	mQTL 6.2	74.04	0.27	S6_13743866 -S6_14881912
33	7	mQTL 7.1	27.53	2.44	S7_3578352 -ud7000557
34	7	mQTL 7.2	57.32	0.59	S7_14598897 -RM1135
35	7	mQTL 7.3	67.13	0.29	S7_18588805–S7_19086057
36	8	mQTL 8.1	3.69	4.29	RM1959 -8024868
37	8	mQTL 8.2	46.09	7.55	S8_586637 -S8_10877789
38	8	mQTL 8.3	59.13	0.2	S8_19884635 -S8_20039575
39	8	mQTL 8.4	67.51	5.51	S8_21050940 -S8_21613952
40	8	mQTL 8.5	94.3	4.79	id8006485 -RM5485
41	8	mQTL 8.6	101.95	2.43	8964581 -S8_25908509
42	8	mQTL 8.7	108.37	0.02	RM3571 -RM6019
43	9	mQTL 9.1	12.52	1.54	9168923 -9186082
44	9	mQTL 9.2	27.21	3.83	9302663 -9361710
45	9	mQTL 9.3	38.96	0.3	S9_14953982 -9466659
46	9	mQTL 9.4	57.94	9.35	S9_17109910 -9688613
47	9	mQTL 9.5	70.75	7.58	S9_20010716 -99776646
48	9	mQTL 9.6	80.63	3.41	9805325 -S9_21917093
49	9	mQTL 9.7	103.29	13.86	RM6797 -RM225
50	9	mQTL 9.8	136.37	2.22	E12M55-4 -E12M48-2
51	9	mQTL 9.9	145.32	0.6	E12M31.1 -C506
52	10	mQTL 10.1	3.05	0.93	9921967 -9941068
53	10	mQTL 10.2	48.98	4.39	S10_15613358 -S10_17272760
54	10	mQTL 10.3	88.51	6.42	S10_21407693 -S10_22060181
55	11	mQTL 11.1	8.66	0.77	S11_2167161 -S11_2379158
56	11	mQTL 11.2	40.96	6.74	S11_5945246 -S11_8343037
57	11	mQTL 11.3	97.86	0.08	S11_23429078 -11819865
58	12	mQTL 12.1	12.68	4.42	RM3483 -S12_2305577
59	12	mQTL 12.2	45.85	12.49	S12_7222741 -S12_15422550
60	12	mQTL 12.3	60.06	0.31	S12_18614318 -12617550
61	12	mQTL 12.4	79.57	8.21	S12_23893471 -S12_25142846
62	12	mQTL 12.5	92.72	3.43	S12_25696421 -S12_25927195
63	12	mQTL 12.6	120.22	9.49	RM7376 -RM212

### QTL hotspots for introgression of salinity tolerance in rice

Meta-QTL analysis identified several genomic regions governing salinity tolerance across the rice genome spanning 12 chromosomes. Among the 63 meta-QTL regions encompassing 5970 genes within 567 initially identified QTLs for salinity tolerance, we propose 15 meta-QTL regions to be QTL hotspots underpinning major traits governing salinity tolerance in rice. These are (Online Resource 2):

- On chromosome 1, mQTL 1.1, mQTL 1.2 and mQTL 1.6 with 26, 23 and 17 initial QTLs governing 20, 17 and 14 traits, respectively;
- On chromosome 2, QTL 2.1 is the major QTL hotspot region with 63 initial QTLs for 37 traits under salinity;
- Three meta-QTLs on chromosome 3, mQTL 3.1, mQTL 3.5 and mQTL 3.6 governing up to 15 traits;
- Two meta-QTLs on chromosome 4, mQTL 4.1 and mQTL 4.9 with 12 and 10 traits;
- On chromosome 5, one meta-QTL mQTL 5.5 governing 15 traits;
- Two meta-QTLs on chromosome 6, meta-QTL 6.1 and meta-QTL6.2 with 17 and 14 traits, and
- On chromosome 9, meta-QTL 9.6 with 12 and 15 traits.

In addition, these meta-QTL regions also possess candidate genes related to a wide range of functions including stress signaling and sensing pathways, genes coding integral membrane components, cell wall organization (wall-associated kinases), serine/threonine (Ser/Thr) kinases, pectinesterases, osmotic adjustment (chitinases, hydrolases), transcription factors regulating stress specific genes, ion homeostasis (Na^+^ and K^+^ transporters and vacuolar Na^+^/H^+^ exchangers) and other related genes. Some of the candidate genes present in these hotspot regions have been validated (Islam et al. 2019; Mirdar-Mansuri et al. 2020), while other needs to be validated for their tolerance in different genetic backgrounds. In addition to these QTL hotspots, there are several genomic regions with 5–10 traits associated with salinity tolerance.

### Candidate genes associated with salinity tolerance

The meta-QTL regions in the present study were mined for potential candidate genes. Integrating differentially expressed genes (DEG’s) identified in microarray studies, RNA-Seq data and the reported candidate genes from 111 published papers resulted in about 60 candidate genes in roots, 4 in shoots, 98 in leaves and 28 in seedlings. Among them, 20 genes localized in the QTL hotspot regions for yield and ion homeostasis are promising potential candidates for enhancing salt tolerance in rice and are validated for differential gene expression using qRT-PCR. Our results are in broad agreement with those of Mirdar-Mansuri et al. (2020) for the families of candidate genes detected in meta-QTL. These potential candidate genes are listed in Online Resource 3 and include pectinesterase, peroxidase, oxidoreductase of the aldo/keto reductase family, inorganic phosphate transporter, transcription regulators and OsHKT1. Over expression of the transcription factor OsNAC45 improves salt and drought tolerance in rice through ABA signal responses and regulation of expression of two specific genes, OsPM1 and OsLEA3-1 (Zhang et al. 2020).

The role of halotolerant genes *HAL1*, *HAL2*, *HAL3*, *HAL4* and *HAL5* encoding proteins with physiological roles in salt stress of rice landraces has been elucidated in addition to those involved in ion homeostasis (Na^+^/H^+^, OsNHX antiporters), compatible organic solutes (glycinebetaine and proline), antioxidative genes (*OsECS*, *OsVTE1*, *OsAPX* and *OsMSRA4.1*), salt responsive regulatory elements and genes encoding protein kinases (MAPKs, SAPKs and STRKs) (Bhatt et al. 2020). Differential expression of genes related to calcium signaling and transport under salinity was observed in IR 64 colonized by an endophyte found in Pokkali (Ramaiah et al. 2020), while a novel halotolerant PGPR strain *Glutamicibacter* sp. YD01 containing ACC deaminase activity-regulating ethylene production confers growth and salt tolerance in rice (Ji et al. 2020). In addition, genes related to ROS, Na^+^/K^+^ homeostasis, rice expansin 7 (OsEXPA7), encoding cell wall-loosening protein, response regulator 22 (OsRR22), a B-type response regulator protein involved in transcription factor regulating genes regulates salinity tolerance in rice (Qin et al. 2020).

### Marker-assisted strategy for introgression of salinity tolerance in rice and rice varieties for salt-affected soils

Conventional breeding methodology involving hybridization followed by progeny screening under stress and recurrent selection led to the development of tolerant lines tested over multi-location trials before release for cultivation (summarized in Islam et al. 2008; Gregorio et al. 2013 for BRRI dhan 47). This involved a participatory approach involving farmers, which helped the adaptation of varieties suitable for specific locations. The Bangladesh Rice Research Institute (BRRI), in collaboration with IRRI, released BRRI dhan 47 (IR63307-4B-4-3) for saline-prone areas in Bangladesh through this participatory approach. Recently, considerable progress has been made in the development of varieties for salinity tolerance through combining traditional breeding and molecular-marker technology. Anther culture-derived dihaploid lines developed from the cross IR5657-33-2 between two *indica* breeding lines IR5657-33-2 × IR4630-22-2-5-1-3 evaluated for salinity tolerance and yield led to the release of a promising line IR51500-AC11-1 as PSBRc50 'Bicol' (Senadhira et al. 2002). However, in spite of more than 50 years of research on the effects of salinity on rice only a part of the knowledge gained has been utilized in applied research to develop of salt-tolerant varieties (Table 7).

**Table 7:** Rice varieties released for salt tolerance globally

Name of the variety	Year of release	Designation	Parentage/Cross combination	Breeding method	Released in Country/State/Provinces	Reference
Jhona 349	1933	Jhona 349	–	Local selection	India	GOI notification No.716 (E), dt.20/2/1970
PVR 1	1968	PVR 1	SR 26-B/MTU 1	Pedigree	India	INGER(https://www.irri.org/inger)
Shwewartun	1974	IR 5mutant	PETA/TANGKAI ROTAN	Mutation breeding	Myanmar	INGER(https://www.irri.org/inger)
Type 100	1978	–	Selected from Bhanslot	Local selection	India	GOI notification No13(E), dt.9/12/1978
Narendra 2 (IET 4555)	1982	IR 2071-625-1- 252	IR 8 /Tadukan//TKM 6 /TN 1//IR 8 × IR 24	Pedigree selection (Introduction from IRRI)	India	GOI notification No165 (E), dt.6/3/1987
Vyttila 2	1982	Culture 174	Pure line sel. from Cheruviruppu	Pureline selection	India	GOI notification No.19 (E),dt 14/1/1982
Vikas (IET 3116)	1983	RP 516-31-6	IR 8/TKM 6	Pedigree selection	India	GOI notification No499 (E),dt.8/7/1983
Panvel 1 (IET 7337)	1984	PNL 5-30	BR 4-10/IR 8	Pedigree selection	India	GOI notification No540 (E),dt.24/7/1985
Panvel 2 (IET 8118)	1984	PNL 32-10-1-1	BR 4-10/IR 8	Pedigree selection	India	GOI notification No.386, dt.15/5/1990
Mohan (CSR 4)	1984		Mutant of IR 8	Mutation breeding	India	INGER(https://www.irri.org/inger)
Usar 1	1984	–	Jaya/Getu	Bulk selection	India	GOI notification No540 (E) dt.24/7/1985
ROK 5	1984		SR 26/Wellington	Bulk selection	Sierra Leone	http://www.fao.org/docrep/006/Y4751E/y4751e09.htm
Sangankhan 4	1985	–		Local selection	Myanmar	INGER(https://www.irri.org/inger)
OM576	1986		HUNGARI/OM 1630-108-2	Pedigree selection	Vietnam	Singh et al. (2010)
CSR 10 (IET 10349)	1989	81-H21-2-4	M 40-431-24-114/Jaya	Pedigree selection	India	GOI notification No. 915 (E), dt.6/11/1989
CST 7-1 (IET 9341)	1989	CST 7-1	Damodar/IR 24	Pedigree selection	India	GOI notification No. 793 (E),dt.22/11/1991
Vyttila 4 (IET 13418)	1991	KAU 906	Chettivirippu/IR 4630-22-2-17	Pedigree selection	India	http://agritech.tnau.ac.in/expert_system/paddy/KLvarieties.html
Lunishree (IET 10678)	1991	CRM 30	Nonasal mutant	Mutation	India	GOI notification No. 814 (E),dt.4/11/1992
WAR 1	1991		IR 4595-4-1-5/Pafant 213	Bulk Method	Sierra Leone	http://www.fao.org/docrep/006/Y4751E/y4751e09.htm
ITA 222	1992	FARO 36	Mahsuri/IET 1444	Bulk Method	Gambia	http://www.fao.org/docrep/006/Y4751E/y4751e09.htm
WAR 1	1992		IR 4595-4-1-5/Pafant 213	Bulk Method	Gambia	http://www.fao.org/docrep/006/Y4751E/y4751e09.htm
WAR 77-3-2-2	1992		IR 4595-4-1-5/Pafant 213	Pedigree selection	Gambia	http://www.fao.org/docrep/006/Y4751E/y4751e09.htm
Sagara	1992	–	Selection from Pureline Orumundakan	Pureline selection	India	GOI notification No. 599 (E),dt.25/4/2006
WAR 1	1993		IR 4595-4-1-5/Pafant 213	Introduction from Sierra Leone	Guinea Bissau	http://www.fao.org/docrep/006/Y4751E/y4751e09.htm
WAR 77-3-2-2	1993		IR 4595-4-1-5/Pafant 213	Introduction from Sierra Leone	Guinea Bissau	http://www.fao.org/docrep/006/Y4751E/y4751e09.htm
Vyttila 5 (IET 14527)	1993	KAU 655	Mutant of Mahsuri	Mutation breeding	India	http://agritech.tnau.ac.in/expert_system/paddy/KLvarieties.html
Giza 177	1994	GZ 4120-205	Giza 171/Yomji No.1//Pi No.4	Recombination breeding	Egypt	Hassan et al. (2013)
Giza 178	1995	GZ 4255-6-3	Giza 175/Milyang 49		Egypt	Hassan et al. (2013)
Narendra Usar Dhan 2 (IET 13556)	1995	NDRK 5020	IRRI Line F2	Selection from introduction	India	GOI notification No. 401 (E),dt.15/5/1998
B 38 D2	1995			NA	Sierra Leone	http://www.fao.org/docrep/006/Y4751E/y4751e09.htm
WAR 73-1-M-1	1996		Rice mill/I. Mahsuri	Recombination breeding	Guinea	http://www.fao.org/docrep/006/Y4751E/y4751e09.htm
WAR 77-3-2-2	1996		IR 4595-4-1-5/Pafant 213	Recombination breeding	Guinea	http://www.fao.org/docrep/006/Y4751E/y4751e09.htm
WAR 1	1997		IR 4595-4-1-5/Pafant 213	Recombination breeding	Senegal	http://www.fao.org/docrep/006/Y4751E/y4751e09.htm
WAR 77-3-2-2	1997		IR 4595-4-1-5/Pafant 213	Recombination breeding	Senegal	http://www.fao.org/docrep/006/Y4751E/y4751e09.htm
WAR 81-2-1-3-2	1997		Miniku 33A/Bayerputih 462-10	Recombination breeding	Senegal	http://www.fao.org/docrep/006/Y4751E/y4751e09.htm
WAR 1	1998		IR 4595-4-1-5/Pafant 213	Recombination breeding	Guinea	http://www.fao.org/docrep/006/Y4751E/y4751e09.htm
CSR 13 (IET 10348)	1998	80-H-3-13	CSR I/Basmati 370//CSR 5	Pedigree selection	India	GOI notification No. 425 (E),dt.8/6/1999
Sakha 104	1999		GZ 4096-8-1/GZ 4100-9-1	Recombination breeding	Egypt	Zayed et al. (2014)
CSR 27 (IET 13765)	1999	CSR-88IR-6	Nona Bokra/IR 5657-33-2	Recombination breeding	India	GOI notification No. 1050 (E),dt.26/10/1999
Narendra Usar Dhan 3 (IET 14659)	1999	IR 46330 (NDRK 14659)	Lung Y AI 148/IR 9125-209-2-2-2-1//IR1872-27-3-1	Selection from introduction	India	GOI notification No. 92 (E),dt.2/2/2001
Panvel 3 (IET 15368)	2000	PNL 18-5-H 7-2	Damodar/Pankaj	Pedigree selection	India	https://aicrp.icar.gov.in/sasusw/wp-content/uploads/2017/07/14.-Coastal-Saline-Soils-of-Maharashtra-Panvel-Centre.pdf
TRY 1 (IET 16643)	2000	TRY 1	BR 153-2B-10-1-3	Introduction from Bangladesh	India	GOI notification No. 92 (E),dt.2/2/2001
TRY (R) 2 (IET 12863)	2000	RP 2597-14-250	IET 6238/IR 36	Recombination breeding	India	GOI notification No. 1134 (E),dt.15/11/2001
OM2717	2000		OM 1738/TN 128	Recombination breeding	Vietnam	http://www.knowledgebank.irri.org/ricebreedingcourse/Breeding_for_salt_tolerance.htm
Dandi (IET 14906)	2001	PNL 2-58-1-1	PNL 2/IET 8320	Pedigree selection	India	GOI notification No. 283 (E),dt.12/3/2003
Basmati CSR 30 (Yamini: IET 14720)	2001	88-H5-1-1-2	Bhura Rata 4-10/Pak. Basmati	Pedigree selection	India	GOI notification No.1134 (E),dt 15/11/2001
CSR 23 (IET 13769)	2002	CSR-89IR-5	IR 64//IR 4630-22-2-5-1-3/IR 9764-45-2-2	Recombination breeding	India	GOI notification No. 161 (E),dt.4/2/2004
OM2517	2002		OM 1352/OMCS 94	Recombination breeding	Vietnam	http://www.knowledgebank.irri.org/ricebreedingcourse/Breeding_for_salt_tolerance.htm
AS996	2002		IR 64/O RUFIPOGON	Wide hybridization	Vietnam	INGER(https://www.irri.org/inger)
BRRI dhan40	2003	BR5331-93-2-8	IR4595-4-1-15/BR10	Recombination breeding	Bangladesh	INGER(https://www.irri.org/inger)
BRRI dhan41	2003	BR5828-11-1-4	BR23/BR1185-2B-16-1	Recombination breeding	Bangladesh	GOI notification No. 161 (E),dt.4/2/2004
Sumati (IET 13428)	2003	CSRC(S)2-1-7	Pankaj/NC 678	Pedigree selection	India	GOI notification No. 1566 (E),dt.5/11/2005
Jarava (IET 15420)	2003	B 90-15	B 32 Sel.4/*O.rufipogon*//B 29-6	Wide hybridization	India	GOI notification No. 1566 (E),dt.5/11/2005
Naina (CSR 36: IET 17340)	2005	CSR 36	CSR 13/Panvel 2//IR 36	Recombination breeding	India	GOI notification No. 1566 (E),dt.5/11/2005
Narendra Usar Sankar Dhan 3 (IET 16651)	2005	NDURH 3	IR 58025 A/NDRK 5026-1 R	Recombination breeding	India	
Vyttila 6	2005	–	Chiriviruppu/IR 5//Jaya	Recombination breeding	India	GOI notification No. 1566 (E),dt.5/11/2005
Bhutnath (IET 12855)	2005	CSRC(S) 5-2-2-5	SR 26B/Pankaj	Pedigree selection	India	GOI notification No. 1572 (E),dt.20/9/2006
Amal Mana (IET 18250)	2006	CSRC(S) 7-1-4	Pankaj/SR 26 B	Pedigree selection	India	GOI notification No. 454 (E),dt.11/2/2009
BRRI Dhan 47	2007	IR63307-4B-4-3	IR 51511-B-B-34-B/TCCP 266-2-49-B-B-3	Introduction from IRRI	Bangladesh	STRASA (IRRI)
CSR 22 (IET 15942)	2008	CSR 22	IR 64/IR 4630-22-2-5-1-3/IR 7969-45-2-2	Recombination breeding	India	GOI notification No. 2187 (E),dt.27/8/2009
DRR Dhan 39 (IET 19487)	2008	RP 4631-46-6-5-1-1-1	CSR 3/Kasturi	Pedigree selection	India	GOI notification No. 211 (E),dt.29/1/2010
Narendra Usar Dhan 2008(IET 18699)	2009	NDRK 5088	TCCP 266-249-B-B-3/IR 262-43-8-1	Selection from Introduction	India	GOI notification No. 2187 (E),dt.27/8/2009
CR Dhan 402(Luna Sampad:IET 19470)	2009	CR 2095-181-1	Mahsuri/Chakrakonda	Recombination breeding	India	https://krishi.icar.gov.in/jspui/bitstream/123456789/31960/1/1.7.pdf
NSIC Rc182 (Salinas 1)	2009	IR63307-4B-4-3	IR 51511-B-B-34-B/TCCP 266-2-49-B-B-3	Recombination breeding	Philippines	https://nseedcouncil.bpinsicpvpo.com.ph/seed%20catalogue.php
NSIC Rc184 (Salinas 2)	2009	PR26016-16-B-B-B	IR 8234-0T-9-2-4-2/GIZA 171	Bulk method	Philippines	https://nseedcouncil.bpinsicpvpo.com.ph/seed%20catalogue.php
NSIC Rc186 (Salinas 3)	2009	PR30244-AC-V2	WAGWAG (AC DERIVED)	Anther culture	Philippines	https://nseedcouncil.bpinsicpvpo.com.ph/seed%20catalogue.php
NSIC Rc188 (Salinas 4)	2009	PR28524-AC97-55	TCCP 266-1-3B-10-2-1/PSB RC 10	Recombination breeding	Philippines	https://nseedcouncil.bpinsicpvpo.com.ph/seed%20catalogue.php
NSIC Rc190 (Salinas 5)	2009	PR25997-B-B-B	IR 9764-45-2-2/IR 81491-AC-5-1	Bulk method	Philippines	https://nseedcouncil.bpinsicpvpo.com.ph/seed%20catalogue.php
BINA Dhan 8	2010	IR66946-3R-149-1-1	IR 29/POKKALI	Introduction from IRRI	Bangladesh	https://strasa.irri.org/
BRRI Dhan 53	2010	BR5778-156-1-3-HR14	BR 10 (BR 51-46-5)//BR 23/BR 847-76-1-1	Recombination breeding	Bangladesh	https://strasa.irri.org/
BRRI Dhan 54	2010	BR5999-82-3-2-HR1	BR 1185-2B-16-1/BR 548-128-1-1-3	Recombination breeding	Bangladesh	https://strasa.irri.org/
CR Dhan 403 (Luna Suvarna:IET 18697)	2010	CR 2096-71-2	Mahsuri/Ormundakan	Recombination breeding	India	https://krishi.icar.gov.in/jspui/bitstream/123456789/31960/1/1.7.pdf
Vytilla 8	2010	–	IR 47310-94-4-3-1/CSR 10	Recombination breeding	India	GOI notification No. 733 (E) dt.1/4/2010
CR Dhan 406 (Luna Barial)	2010	CR 2092-158-3	Jaya /Lunishree	Recombination breeding	India	https://krishi.icar.gov.in/jspui/bitstream/123456789/31960/1/1.7.pdf
Jagjeevan (IET 19487)	2010	RP 4631-46-6-5-1-1-1	CSR 3/ Kasturi	Recombination breeding	India	GOI notification S.O. 211 (E), dt 1/29/2010
BRRI Dhan 55	2011	IR73678-6-9-B (AS996)	IR 73382-121/IR 64	Wide hybridization (Introduction from IRRI)	Bangladesh	https://strasa.irri.org/
Pyi Myanmar Sein (IR10T107)	2011	IR 83412-6-B-3-1-1(NSIC 110)	IRRI 126/IR 71606-1-1-4-2-3-1-2	Recombination breeding (Introduction from IRRI)	Myanmar	http://cure.irri.org/events/myanmarpartnersproducehigh-yieldingsalinity-tolerantricevarieties
Shew ASEAN (CSR36)	2011	CSR 36	CSR 13/Panvel 2//IR 36	Recombination breeding (Introduction from India)	Myanmar	http://cure.irri.org/events/myanmarpartnersproducehigh-yieldingsalinity-tolerantricevarieties
NSIC Rc290 (Salinas 6)	2011	PR28377-AC97-54	IRRI 113/PSB RC 10 (IR 50404-57-2-2-3)	Anther culture	Philippines	https://strasa.irri.org/
NSIC Rc292 (Salinas 7)	2011	PR30244-AC-V19	WAGWAG (AC DERIVED)	Anther culture	Philippines	https://nseedcouncil.bpinsicpvpo.com.ph/seed%20catalogue.php
NSIC Rc294 (Salinas 8)	2011	PR28378-AC96-36	IRRI 113/IR 64	Anther culture	Philippines	https://strasa.irri.org/
NSIC Rc296 (Salinas 9)	2011	IR71896-3R-8-3-1	IR 55182-3B-14-3-2/IR 65195-3B-13-2-3 (PSB RC 86)	Pedigree method	Philippines	https://strasa.irri.org/
BINA Dhan 10	2012	IR64197-3B-14-2	IR 42598-B-B-B-B-12/NONA BOKRA	Recombination breeding (Introduction from IRRI)	Bangladesh	https://strasa.irri.org/
CR Dhan 405 (Luna Sankhi)	2012	IR 72046-B-R-8-3-1-3	IR 31406-333-1/2*IR 31142-14-1-1-3-2	Recombination breeding (Introduction from IRRI)	India	https://krishi.icar.gov.in/jspui/bitstream/123456789/31960/1/1.7.pdf
CSR 43 (IET 18259)	2012	CSR-89IR-8	KDML 105/IR 4630-22-2-5-1-3//IR 20925-33-3-1-28	Recombination breeding	India	GOI notification S.O. 244 (E) dt.1/24/2014
Gosaba 5 (IET 23403)	2012	Chinsurah Nona 1 (IR 55179-3B-11-3)	IR 4630-22-2-5-1-3/Nonabokra	Recombination breeding	India	GOI notification No. S.O.3540(E) dt.22/11/2016
BRRI Dhan 61	2013	BR7105-4R-2	IR64419-3B-4-3	Selection from Introduction	Bangladesh	https://strasa.irri.org/
Sangankhan Sinthwelatt	2013	Yn 3220 MAS 62-2-4	IR 53936-60-3-2-1/ Pokkali	Recombination breeding	Myanmar	https://strasa.irri.org/
Saltol Sin thew Latt	2013	SarNganKhan Sin Thwe Latt	IR53936-60-3-2-3-1/Pokkali	MAS product	Myanmar	https://strasa.irri.org/
NSIC Rc324 (Salinas 10)	2013	PR31607-2-B-B-B-B	IR 65185-3B-8-3-2 (PSB RC 84)/ASOMINORI	Bulk method	Philippines	https://strasa.irri.org/
NSIC Rc326 (Salinas 11)	2013	IR84084-B-B-1-1	IR 66946-3R-178-1-1/2*IR 64680-81-2-2-1-3	Back crossing	Philippines	https://strasa.irri.org/
NSIC Rc328 (Salinas 12)	2013	IR62700-2B-9-2-3	IR 8192-200-3-3-1-1//BG 367-4/SUAKOKO 8	Recombination breeding	Philippines	https://strasa.irri.org/
NSIC Rc330 (Salinas 13)	2013	PR37435-30-1	PSB RC 90/PR 29264-AC10//IR 64-1-1-4/IR 70030-7-2-2-1-2	Recombination breeding	Philippines	https://strasa.irri.org/
NSIC Rc332 (Salinas 14)	2013	PR38566-WAGWAG V9-3- 2-15-2		Selection	Philippines	https://strasa.irri.org/
NSIC Rc334 (Salinas 15)	2013	IR83410-6-B-4-1-1-2	IRRI 126/IR 64680-81-2-2-1-3	Pedigree method	Philippines	https://strasa.irri.org/
NSIC Rc336 (Salinas 16)	2013	IR84095-AJY3-8-SD01-B	IR 68144-2B-2-2-3-1/IR 66946-3R-78-1-1//IR 77080-B-4-2-2	Shuttle breeding	Philippines	https://strasa.irri.org/
NSIC Rc338 (Salinas 17)	2013	PR30665-1B-1-B-B-Cg	IR 52717-B-B-4-B-B-1-3//IR 9884-54-3/NONA BOKRA///POKKALI	Recombination breeding	Philippines	https://strasa.irri.org/
NSIC Rc340 (Salinas 18)	2013	IR84096-AJY 4-2-SDO- 4-B	IR 72593-B-3-2-3-3/IR 72875-94-3-3-2//IR 66946-3R-156-1-1	Shuttle breeding	Philippines	https://strasa.irri.org/
BRRI Dhan 65	2014	OM1490	OM606/IR44592-62-1-1-3	Recombination breeding (Introduction from Vietnam)	Bangladesh	https://strasa.irri.org/
BRRI Dhan 69	2014	BR 7100-R-6-6	IR61247-3B-8-2-1/ BRRI dhan36	Recombination breeding	Bangladesh	https://strasa.irri.org/
ITA 212	2014	FARO 35	BG 90-2*4/TETEP	Back Cross	Gambia	https://strasa.irri.org/
ARICA 11	2014	IR 63275-B-1-1-1-3-3-2	IR 68/TCCP 266-2-49-B-B-3	Pedigree method	Gambia	https://strasa.irri.org/
CSR 46	2014		IR72/CSR23	Recombination breeding	India	S.O. No. 6318(E) dt.26/12 2018
Inpari 34 Salin Agritan ((NSIC RC 106)	2014	IR78788-B-B-10-1-2-4-AJY1	BR 41/IR 61920-3B-22-2-1	Modified bulk pedigree	Indonesia	ASEAN%20Regional%20Guidelines%20on%20Promoting%20CSA%20Practices-endorsed%2037th%20AMAF.pdf
Inpari 35 Salin Agritan	2014	CSR-90IR-2	IR 10206-29-2-1/SUAKOKO (SEL)	Recombination breeding	Indonesia	ASEAN%20Regional%20Guidelines%20on%20Promoting%20CSA%20Practices-endorsed%2037th%20AMAF.pdf
NSIC Rc390 (Salinas 19)	2014	IR 83140-B-28-B (IRRI 184)	IR 82869-11/IR 82870-11	Modified bulk pedigree	Philippines	https://strasa.irri.org/
NSIC Rc392 (Salinas 20)	2014	IR 84675-58-4-1-B-B (IRRI 185)	IR 64*3/MADHUKAR//IR 64*3/BINAM	Modified bulk pedigree	Philippines	https://strasa.irri.org/
Rohyb 183-B-5-B-1	2014	WAB0006141		Recombination breeding	Sierra Leone	http://www.fao.org/3/y4347e/y4347e1l.htm
Rohyb 162- B-1	2014			Recombination breeding	Sierra Leone	http://www.fao.org/3/y4347e/y4347e1l.htm
ROK 37 (WAR77-3-2-2)	2014	WAR77-3-2-2	IR 4595-4-1-5/PA FANT 213	Pedigree selection	Sierra Leone	http://www.fao.org/3/y4347e/y4347e1l.htm
NERICA-L 20	2014	WAS 122-IDSA-1-WAS	WAB 1291/3*IR 64	Backcross	Sierra Leone	http://www.fao.org/3/y4347e/y4347e1l.htm
BR 78 (BRRI Dhan 78)	2016		IR 127-80-1-10/PANBIRA//IR 297-9-1-3-2-2-2	Recombination breeding with help of MAS	Bangladesh	https://strasa.irri.org/
CSR 49	2016		CSR-2 K-242	Recombination breeding	India	GOI notification No. S.O. 3220(E) dt5/09/2019
NSIC Rc462 (Salinas 21)	2016	PR30025-99AC-WSAL-1086	PR30025-99AC-WSAL-1086	Anther culture	Philippines	https://nseedcouncil.bpinsicpvpo.com.ph/seed%20catalogue.php
NSIC Rc464 (Salinas 22)	2016	IR86385-38-1-1-B	IRRI 149/IRRI 128	Recombination breeding with help of MAS	Philippines	https://nseedcouncil.bpinsicpvpo.com.ph/seed%20catalogue.php
NSIC Rc466 (Salinas 23)	2016	IR84089-7-3-AJY1-B	IR66946-312-178-1-1/IR72875-94-3-3-2//IR72875-94-3-3-2	Pedigree Selection	Philippines	https://nseedcouncil.bpinsicpvpo.com.ph/seed%20catalogue.php
NSIC Rc468 (Salinas 24)	2016	IR06M139	IR72158-16-3-3/IR74646-96-2-3-3	Recombination breeding	Philippines	https://nseedcouncil.bpinsicpvpo.com.ph/seed%20catalogue.php
NSIC Rc470 (Salinas 25)	2016	PR30245-IR640-ID-18-1-4	IR64/AC97WP (Anther culture)	Anther culture	Philippines	https://nseedcouncil.bpinsicpvpo.com.ph/seed%20catalogue.php
CSR 52	2017		CSR 23/CSR 27	Recombination breeding	India	GOI notification No. S.O. 3220(E) dt5/09/2019
ROK 35 (No. 1 B.P 148)	2017			Selection	Sierra Leone	http://www.fao.org/3/y4347e/y4347e1l.htm
Vyttila 3		–	Vyttila 1/TN 1	Recombination breeding	India	

Pooling of physiological traits was suggested (Flowers and Yeo 1995) and proved successful in generating tolerant lines of rice for salinity tolerance (Gregorio et al. 2002; Thomson et al. 2010; Singh et al. 2021) and other abiotic stresses (Ali et al. 2017). Initially, screening of available germplasm resources to explore the natural variability for salt tolerance led to identification of promising genotypes which were further utilized in breeding for further improvement. Generation advance through conventional methods requires several years to stabilize the variability created before a cultivar is released for farmer cultivation. Recently, a technique called ‘Speed Breeding’ or Rapid Generation Advance (RGA) has been proposed that produces 4–5 generations per year. While there is much recent work in this area (Tanaka et al. 2016; Collard et al. 2017), it is out of the scope of this review, which is focused on reproductive stage phenotyping for salinity stress. Physiological parameters linked to salinity tolerance formed the basis of grouping the genotypes (Asch et al. 2000; Zeng et al. (2003), while other studies considered both physiological traits and yield as well as agronomic characteristics (Li and Xu 2007; Natarajan et al. 2005; Zeng and Shannon, 2000a,b; Zeng et al. 2002; Zeng et al. (2003). The complexity of breeding for salinity tolerance lies with the varied levels of tolerance during crop growth, differences between genotypes that are poorly correlated, with some reports indicating independent inheritance. Hence pyramiding of genetic components controlling tolerance at different growth stages could be the best approach.

Bohnert and Jensen (1996) suggested that successful releases of tolerant varieties of crop plants require large-scale metabolic engineering that include transfer of many genes. Despite the difficulties of dealing with physiological complexity of traits that are determined by sub-components each with a different set of genes in number and quantum effect (Flowers et al. 2000), MAS and marker-assisted backcrossing (MABC) have been vital tools in the transfer of tolerance-related genes/QTLs for the effects of drought, salinity and submergence to elite lines of rice. Several physiological mechanisms and their underlying genomic regions (genes/QTLs) have been tagged. Flanking markers have been found to differ in their reliability and stability for specific QTLs: for example, RM8094 and RM 3412 were found reliable and diagnostic for *Saltol,* being more closely flanking than others markers like RM 493 (Islam et al. 2012; Al-Amin et al. 2013; Babu et al. 2014). The successful utilization of MAS for salinity tolerance is illustrated by introgression of *Saltol* into several elite varieties of different countries like PB1121 and PB6 in India (Singh et al. 2011), AS996 BT7, Bac Thom 7 and Q5BD in Vietnam (Huyen et al. 2012, 2013; Linh et al. 2012; Vu et al. 2012; Luu et al. 2012), BRRI dhan 49 in Bangladesh (Hoque et al. 2015) and Novator in Russia (Usatov et al. 2015). Singh et al. 2016 reported the introgression of *Saltol* into seven popular varieties (ADT45, CR1009, Gayatri, MTU1010, PR114, Pusa 44 and Sarjoo 52) through a multi-institutional network project 'From QTL to variety: marker assisted breeding of abiotic stress tolerant rice varieties with major QTLs for drought, submergence and salt tolerance' a collaborative project of India and IRRI, Philippines. *Saltol* introgressed QTLs NILs in the genetic background of Pusa 44 and Sarjoo 52, the high yielding mega varieties of India, exhibited improved salinity tolerance at the seedling stage salinity (Krishnamurthy et al. 2020).

Coastal areas, especially in the wet season, need rice varieties not only with salinity tolerance but also submergence tolerance due to frequent inundation of the crop either by tides or estuarine river outflow. IRRI developed a number of salinity and submergence tolerance lines (like IR84649-81-4-B-B and IR84645-311-22-1-B) through introgression of *Saltol* from ‘Pokkali’ a saline-tolerant landrace and *Sub* 1 from Swarna-*Sub*1, a submergence-tolerant variety, through marker-assisted backcrossing (Gregorio et al. 2013). Recently, two dual tolerant two-in-one IRRI-derived rice varieties, BRRI dhan 78 in Bangladesh and Salinas 22 (IR86385-38-1-1-B) in Philippines were released for commercial cultivation (Table 7).

## Conclusion

Recent developments in understanding the responses rice to salt stress need to be integrated to supplement conventional rice breeding and harness the maximum genetic improvement for salinity tolerance. A simple, reliable efficient phenotyping method, availability of adequate genetic variability, knowledge of the genetic control and physiological mechanisms governing salinity stress are tools a breeder can use to improve salt tolerance. For rice, a species whose tolerance to salinity varies over its life, understanding the mechanisms involved at different growth stages is of the utmost importance for generating genotypes that are salt-tolerant throughout the crop growing period. Various phenotyping methods, focused on screening at the seedling stage, have been proposed and utilized to develop varieties with enhanced salt tolerance. However, screening at the reproductive stage, which is more complex than screening seedlings but particularly important as tolerance at this later stage translates into grain yield, has yet to be utilized. A novel approach to phenotyping for reproductive stage screening, developed at IRRI since 2013 (Calapit-Palao et al 2013), has been described in detail. An inventory of salt-tolerant donors was made that can be used in breeding programs for generating breeding material with broad genetic base. A comprehensive compilation of previously reported QTLs for salinity tolerance has also been made. However, QTL confidence intervals are often too large to be utilized in marker-assisted introgression. Hence, a meta-QTL analysis was conducted to integrate the genetic linkage maps of different studies utilized for individual QTL mapping into a single consensus linkage map. Meta-analysis has redefined the confidence interval of QTL to a smaller physical and genetic interval that facilitates the identification of candidate genes for salinity tolerance. Validation of these meta-QTLs and the candidate genes would further facilitate their utilization and introgression through breeding programs. Conventional breeding combined with molecular and genomic approaches has supported the development of salt-tolerant rice varieties. However, the association between seedling and reproductive stage tolerance is known to be poor. Furthermore, none of the salt-tolerant donors possess all the desirable alleles for all salt-tolerant mechanisms. A recombination breeding strategy that involves combining all the favorable and complementary traits into a genetic background without any yield penalty would pave the way for the development of a variety with an outstanding performance in farmers’ fields throughout the growing period.

## Supplementary Information

Below is the link to the electronic supplementary material.

Supplementary file1 (DOCX 242 kb)

Supplementary file2 (DOCX 24 kb)

Supplementary file3 (DOCX 52 kb)
