# Supplementary material for: Salt tolerance in rice: seedling and reproductive stage QTL mapping come of age
Source: Theor Appl Genet. 2021 Jul 21;134(11):3495–533. doi: 10.1007/s00122-021-03890-3 (PMC8519845; doi:10.1007/s00122-021-03890-3)
Supplement: Supplementary file 1 — Supplementary file1 (DOCX 242 kb) [file 122_2021_3890_MOESM1_ESM.docx]

**Online Resource 1** Details of the reported QTL studies in rice for salinity tolerance

| **QTL study/ reference** | **Cross combination** | **Population type** | **Population size** | **QTL** | **Trait governing QTL** | **QTL position (Mb/** cM**)*** | **Chr** | **LOD/ P- Value** | **PV (%)** | **Flanking markers** |
| --- | --- | --- | --- | --- | --- | --- | --- | --- | --- | --- |
| Claes et al.(1990) | Fixed line | Fixed line | TN1 | *sal*T | Na+ accumulation in roots | **13.8** | 1 |  |  | RG146B |
| Gregorio (1997) | IR 29/ Pokkali | F8 RILs | 80 | *Saltol* | *Saltol/K absorption* | 14.7 | 1 | 17.23 | 80.2 | P3/M9-8-*Saltol* |
|  |  |  |  | *Saltol* | *Saltol/Na absorption* | 14.7 | 1 | 14.54 | 64.6 | P3/M9-8-*Saltol* |
|  |  |  |  | *Saltol* | *Saltol/Na -K Ratio* | 14.7 | 1 | 14.51 | 64.3 | P3/M9-8-*Saltol* |
|  |  |  |  | *qSRTL-6* | seedling root length | 106.1 | 6 | 2.85 | 18.9 | RG162-RG653 |
|  |  |  |  | *qSDM-6* | seedling dry matter | 120 | 6 | 2.47 | 16.7 | CDO544-Amy2A |
|  |  |  |  | Trait based QTL | Shoot weight | 79.65 | 11 | >3 | 14.38 | RM209-RM206 |
|  |  |  |  | Trait based QTL | K+Conc | 53.62 | 12 | >3 | 17.45 | G24-R1684 |
|  |  |  |  | Trait based QTL | Na+ Conc | 132.3 | 2 | >3 | 7.22 | RM240- RM213 |
|  |  |  |  | Trait based QTL | Na+/K+ ratio | 141.53 | 1 | >3 | 9.14 | C86-RM212 |
|  |  |  |  | Trait based QTL | Na+/K+ ratio | 27.94 | 7 | >3 | 5.86 | RM214-RM1789 |
|  |  |  |  | Trait based QTL | Na+/K+ ratio | 53.62 | 12 | >3 | 8.81 | G24-R1684 |
|  |  |  |  |  |  |  |  |  |  |  |
| Lang et al. (2001B) | Tesanai 2/CB | F8 RILs | 108 | qNDS-1.0 | No. of days Survival | 98.2 | 1 | 3 | 5.8 | C178 |
|  |  |  |  | qSNaKR-1 | Shoot Na/K Ratio | 33 | 1 | 3 | 9.14 | C86 |
|  |  |  |  | qNDS-2.1 | No. of days Survival | 122.1 | 2 | 3 | 6.6 | C560 |
|  |  |  |  | qNDS-2.2 | No. of days Survival | 134 | 2 | 3 | 6.1 | C747 |
|  |  |  |  | qNDS-2.3 | No. of days Survival | 45.8 | 2 | 3 | 5.2 | R26 |
|  |  |  |  | qSNC-2 | Shoot Na Conc | 22.65 | 2 | 3 | 7.22 | RM240 |
|  |  |  |  | qSNaKR-2 | Shoot Na/K Ratio | 134 | 2 | 3 | 5.25 | C747 |
|  |  |  |  | qNDS-3 | No. of days Survival | 211.25 | 3 | 3 | 8.5 | R3156 |
|  |  |  |  | qRW-3.1 | Root Weight | 211.25 | 3 | 3 | 9.34 | R3156 |
|  |  |  |  | qRW-3.2 | Root Weight | 194.7 | 3 | 3 | 6.95 | C563 |
|  |  |  |  | qRW-3.3 | Root Weight | 141.8 | 3 | 3 | 6.35 | C397 |
|  |  |  |  | qSNaKR-7 | Shoot Na/K Ratio | 63.6 | 7 | 3 | 5.86 | RM214 |
|  |  |  |  | qNDS-9.1 | No. of days Survival | 106.2 | 9 | 3 | 11.5 | C711 |
|  |  |  |  | qNDS-9.2 | No. of days Survival | 125.7 | 9 | 3 | 11.6 | C1454 |
|  |  |  |  | qNDS-9.3 | No. of days Survival | 150.2 | 9 | 3 | 6.9 | R1751 |
|  |  |  |  | qSW-11.0 | Shoot weight | 156 | 11 | 3 | 14.38 | RM209 |
|  |  |  |  | qSKC-12 | Shoot K Conc. | 35.5 | 12 | 3 | 17.45 | G24 |
|  |  |  |  | qSNaKR-12 | Shoot Na/K Ratio | 35.5 | 12 | 3 | 8.81 | G24 |
|  |  |  |  |  |  |  |  |  |  |  |
| Koyama et al. (2001) | IR55178 (IR4630-22-2-5-1-3/IR15324-117-3-2-2) | F6 RILs | 118 | qNU-1 | Na+ Uptake | 74 | 1 | 3 | 8.9 | E12M63-1 to E12M48-6 |
|  |  |  |  | qKC-1 | K+ Conc. | 56 | 1 | 3 | 10.6 | E12M46-7 to E12M37-1 |
|  |  |  |  | qNKR-1 | Na:K ratio | 74 | 1 | 3 | 9.1 | E12M63-1 to E12M48-6 |
|  |  |  |  | qKU-4 | K+ Uptake | 10 | 4 | 3 | 6.8 | E12M65-1 toE12M3812 |
|  |  |  |  | qKC-4 | K+ Conc. | 90 | 4 | 3 | 8.8 | E15M53-2 to E12M79-4 |
|  |  |  |  | qNC-4 | Na+ Conc. | 24 | 4 | 3 | 6.7 | RM261 to E12M60-1 |
|  |  |  |  | qNKR-4 | Na+:K+ ratio | 14 | 4 | 3 | 9.6 | E12M65-1 to RM 261 |
|  |  |  |  | qDM-6 | Dry mass | 34 | 6 | 3 | 9.7 | E12M80-2 to E12M37-2 |
|  |  |  |  | qKU-6 | K+ Uptake | 30 | 6 | 3 | 7.6 | E12M80-2 to RZ413B |
|  |  |  |  | qNC-6 | Na+ Conc. | 106 | 6 | 3 | 6.4 | E12M35-3 to E12M87-8 |
|  |  |  |  | qKU-9 | K+ Uptake | 96 | 9 | 3 | 19.6 | E12M55-4 to RM 205 |
|  |  |  |  |  |  |  |  |  |  |  |
| Bonilla et al. (2002) | IR 29/ Pokkali | F8 RILs | 54 | *saltol-qNU-1* | *Saltol Na+ Uptake* | 63.6 | 1 | 5.8 | 39.2 | RM140-C1733S |
|  |  |  |  | *saltol-qKU-1* | *Saltol K+ uptake* | 63.6 | 1 | 6.8 | 43.9 | RM140-C1733S |
|  |  |  |  | *saltol-qNKR-1* | *Saltol Na:K ratio* | 63.6 | 1 | 6.6 | 43.2 | RM140-C1733S |
|  |  |  |  |  |  |  |  |  |  |  |
| Niones (2004) | IR 29/ Pokkali | BC3F4 NILs | 3000 | *saltol*-qNU-KU-NKR-1 | *Saltol ion uptake and Na/K ratio* | 68.9 | 1 | 5.02 | 0.44 | CP6224 - RM 8094 |
|  |  |  |  | *saltol*-qNU-KU-NKR-1 | *Saltol ion uptake and Na/K ratio* | 67 | 1 | 3.34 | 0.44 | CP6224 - RM 8094 |
|  |  |  |  |  |  |  |  |  |  |  |
| Lin et al. (2004) | Nona Bokra/Koshihikari | F2:F3 | 133 | *qSDS-1* | Seedling survival | 188.95 | 1 | 4.88 | 18 | C813-C86 |
|  |  |  |  | *qSKC-1* | Shoot K+ concentration | 82.5 | 1 | 11.74 | 40.1 | C1211-S2139 |
|  |  |  |  | *qRNTQ-1* | Root Na+ total quantity | 189 | 1 | 3.25 | 12.4 | C813-C86 |
|  |  |  |  | *qRKC-4* | Root K+ concentration | 13.3 | 4 | 4.28 | 21.6 | C891-C513 |
|  |  |  |  | *qSDS-6* | Seedling survival | 104.6 | 6 | 3.63 | 17 | C214-R2549 |
|  |  |  |  | *qSDS-7* | Seedling survival | 51.7 | 7 | 3.32 | 13.9 | R2401-L538T7 |
|  |  |  |  | *qSNC-7* | Shoot Na+ concentration | 31.7 | 7 | 7.66 | 48.5 | C1057-R2401 |
|  |  |  |  | *qSNTQ-7* | Shoot Na+ total quantity | 31.7 | 7 | 4.26 | 16.1 | C1057-R2401 |
|  |  |  |  | *qRKC-7* | Root K+ concentration | 31.7 | 7 | 3.48 | 17.8 | C1057-R2401 |
|  |  |  |  | *qRKTQ-7* | Root K+ total quantity | 31.7 | 7 | 3.82 | 17.3 | C1057-R2401 |
|  |  |  |  | *qRNC-9* | Root Na+ concentration | 71.5 | 9 | 3.25 | 16.7 | R1751-R2638 |
|  |  |  |  |  |  |  |  |  |  |  |
| Takehisa et al. (2004) | Nipponbare/Kasalath//Nipponbare | BC_1_F_9_ to BC_1_F_12_ | 98 | *qSL-1.1* | Shoot length | 32.5 | 1 | 4.39 | 22 | C813/C86 |
|  |  |  |  | *qSL-1.2* | Shoot length | 19.25 | 1 | 6.38 | 30 | R2414/C742 |
|  |  |  |  | *qSL-1.3* | Shoot length | 19.25 | 1 | 5.16 | 24 | R2414/C742 |
|  |  |  |  | *qSL-1.4* | Shoot length | 19.25 | 1 | 2.44 | 12 | R2414/C742 |
|  |  |  |  | *qTN-2.1* | Tiller number | 32.8 | 2 | 2.22 | 12 | C747/R3393 |
|  |  |  |  | *qTN-2.2* | Tiller number | 22.65 | 2 | 4.26 | 23 | C1408/C560 |
|  |  |  |  | *qTN-2.3* | Tiller number | 22.65 | 2 | 2.43 | 12 | C1408/C560 |
|  |  |  |  | *qTN-2.4* | Tiller number | 22.65 | 2 | 2.68 | 13 | C1408/C560 |
|  |  |  |  | *qSL-3.1* | Shoot length | 17.05 | 3 | 2.99 | 14 | C944/C595 |
|  |  |  |  | *qSL-3.2* | Shoot length | 17.05 | 3 | 5.3 | 24 | C944/C595 |
|  |  |  |  | *qSL-3.3* | Shoot length | 30.7 | 3 | 2.3 | 14 | R250/C136 |
|  |  |  |  | *qSL-3.4* | Shoot length | 30.7 | 3 | 2.8 | 17 | R250/C136 |
|  |  |  |  | *qSL-7.1* | Shoot length | 32.6 | 7 | 3.9 | 18 | R2401/R1488 |
|  |  |  |  | *qSL-7.2* | Shoot length | 19.45 | 7 | 3.45 | 16 | R1488 |
|  |  |  |  | *qSL-7.3* | Shoot length | 10.15 | 7 | 4.45 | 23 | C1057/R565 |
|  |  |  |  | *qSL-7.4* | Shoot length | 32.6 | 7 | 4.71 | 22 | R2401/R1488 |
|  |  |  |  |  |  |  |  |  |  |  |
| Ren et al.(2005) | Nona Bokra/Koshihikari | BC3F2 | 192 | *qSKC-1* | Shoot K+ concentration | 38.4 | 1 | 11.74 | 40.1 | K159-K061 |
|  |  |  |  |  |  |  |  |  |  |  |
| Yao et al.(2005) | Jiucaiqing/IR36 | F2 | 250 | qSTR-1 | Salinity tolerance rating | 122.3 | 1 | 2.06 | 6.7 | RM9-RM128 |
|  |  |  |  | qNAK-2 | Na+/K+ ratio in roots | 89 | 2 | 2.73 | 19.3 | RM318-RM262 |
|  |  |  |  | qSTR-5 | Salinity tolerance rating | 20.7 | 5 | 2.72 | 14.3 | RM161-RM13 |
|  |  |  |  | qNAK-6 | Na+/K+ ratio in roots | 7.2 | 6 | 2.03 | 7.9 | RM176-RM345 |
|  |  |  |  | qDWS-8 | Dry matter weight of shoot | 101.8 | 8 | 2.47 | 7.5 | RM223-RM152 |
|  |  |  |  | qSTR-9 | Salinity tolerance rating | 10.7 | 9 | 2.06 | 7 | RM278-RM215 |
|  |  |  |  | qDWS-9 | Dry matter weight of shoot | 10.7 | 9 | 2.31 | 11.5 | RM278-RM215 |
|  |  |  |  |  |  |  |  |  |  |  |
| Lee et al 2006 | Milyang 23/Gihobyeo | RIL (F19) | 164 | *qST1* | seedling tolerance | 194.15 | 1 | 11.58 | 27.76 | Estl-2 &RZ569A |
|  |  |  |  | *qST3* | seedling tolerance | 107.25 | 3 | 3.42 | 9.16 | RG179-RZ596 |
|  |  |  |  |  |  |  |  |  |  |  |
| Takehisa et al. (2006) | Nipponbare/Kasalath//Nipponbare | BILs | 98 | *qLB-3* | Leaf bronzing | 2.4 | 3 | 31.7 | 83 | R1925 |
|  |  |  |  | *qLB-11* | Leaf bronzing | 67.3 | 11 | 3.5 | 8 | C1350-C477 |
|  |  |  |  |  |  |  |  |  |  |  |
| Ammar et al. (2007) | CSR 27/MI48 | F2:F3 population | 200 | qSIS-1.1 | seedling stage salt injury | 29.35 | 1 | 2.8 | 5.77 | RM84-RM259 |
|  |  |  |  | qSIS-1.2 | seedling stage salt injury | 53.35 | 1 | 2.33 | 14.38 | RM572-RM294 |
|  |  |  |  | qSIS-3 | seedling stage salt injury | 57.75 | 3 | 2.62 | 25.8 | RM563-RM186 |
|  |  |  |  | qSIS-4.1 | seedling stage salt injury | 100.7 | 4 | 2.32 | 5.13 | RM5320-RM3648 |
|  |  |  |  | qSIS-4.2 | seedling stage salt injury | 121.6 | 4 | 2.54 | 7.11 | RM3648-RM280 |
|  |  |  |  | qSIS-5 | seedling stage salt injury | 108.7 | 5 | 2.24 | 8.7 | RM233B-RM334 |
|  |  |  |  |  |  |  |  |  |  |  |
| Sabouri and Sabouri (2008) | Tarommahalli/Khazar | F2:F3 | 192 | *qPL-2* | plant stand | 59.6 | 2 | 3.15 | 16.45 | RM5699-RM262 |
|  |  |  |  | *qCHLC-3* | chlorophyll content | 48.4 | 3 | 4.67 | 14.5 | RM1022-RM6283 |
|  |  |  |  | *qRL-1* | Root length | 79.7 | 1 | 2.7 | 13.63 | RM8068-RM8231 |
|  |  |  |  | *qRL-4* | Root length | 63.3 | 4 | 2.91 | 11.56 | RM5473-RM551 |
|  |  |  |  | *qRL-5* | Root length | 102.6 | 5 | 3.67 | 14.8 | RM421-RM480 |
|  |  |  |  | *qRL-7* | Root length | 78.8 | 7 | 4.24 | 16.21 | RM1048-RM11 |
|  |  |  |  | *qRL-9a* | Root length | 84 | 9 | 4.47 | 14.12 | RM1553-RM5702 |
|  |  |  |  | *qRL-9b* | Root length | 66.3 | 9 | 3.58 | 11.51 | RM7424-RM5702 |
|  |  |  |  | *qSHL-3* | Root length | 139.9 | 3 | 2.69 | 23.57 | RM7389-RM7000 |
|  |  |  |  | *qSHL-10* | shoot length | 30.9 | 10 | 5.3 | 19.19 | RM7545-RM4455 |
|  |  |  |  | *qGLA-3* | Green leaf area | 48.4 | 3 | 5.54 | 12.81 | RM1022-RM6283 |
|  |  |  |  | *qFWSH-1* | Fresh weight shoot | 121.8 | 1 | 3.36 | 22.44 | RM8235-RM8144 |
|  |  |  |  | *qFWSH-3* | Fresh weight shoot | 48.4 | 3 | 5.01 | 22.97 | RM1022-RM6283 |
|  |  |  |  | *qFWRO-3a* | Fresh weight root | 48.4 | 3 | 4.7 | 20.91 | RM1022-RM6283 |
|  |  |  |  | *qFWRO-3b* | Fresh weight root | 78.7 | 3 | 4.64 | 17.72 | RM6283-RM6832 |
|  |  |  |  | *qDWSH-3* | Dry weight shoot | 48.4 | 3 | 4.46 | 23.21 | RM1022-RM6283 |
|  |  |  |  | *qDWSH-7* | Dry weight shoot | 70.9 | 7 | 2.84 | 23.17 | RM5481-RM11 |
|  |  |  |  | *qDWRO-3* | Dry weight root | 48.4 | 3 | 3.58 | 21.41 | RM1022-RM6283 |
|  |  |  |  | *qDWRO-5a* | Dry weight root | 102.6 | 5 | 2.65 | 21.74 | RM421-RM480 |
|  |  |  |  | *qDWRO-5b* | Dry weight root | 94.5 | 5 | 2.87 | 22.55 | RM480-RM440 |
|  |  |  |  | *qDWRO-9a* | Dry weight root | 75.2 | 9 | 3.24 | 27.43 | RM1553-RM7424 |
|  |  |  |  | *qDWRO-9b* | Dry weight root | 66.3 | 9 | 2.95 | 25.5 | RM7424-RM5702 |
|  |  |  |  | *qNAUP-1a* | Na+ uptake | 94.8 | 1 | 2.84 | 13.03 | RM562-RM543 |
|  |  |  |  | *qNAUP-1b* | Na+ uptake | 77.1 | 1 | 5.6 | 22.17 | RM8068-RM8231 |
|  |  |  |  | *qNAUP-3* | Na+ uptake | 112.2 | 3 | 2.81 | 13.62 | RM416-RM5626 |
|  |  |  |  | *qNAUP-9a* | Na+ uptake | 75.2 | 9 | 4.97 | 17.71 | RM1553-RM7424 |
|  |  |  |  | *qNAUP-9b* | Na+ uptake | 66.3 | 9 | 4.43 | 16.95 | RM7424-RM5702 |
|  |  |  |  | *qNAUP-10* | Na+ uptake | 30.9 | 10 | 3.8 | 13.84 | RM7545-RM4455 |
|  |  |  |  | *qKUP-3* | K+ uptake | 48.4 | 3 | 4.48 | 22.15 | RM1022-RM6283 |
|  |  |  |  | *qKUP-8* | K+ uptake | 7.3 | 8 | 5.16 | 38.22 | RM4955-RM152 |
|  |  |  |  | *qNAKUP-6* | Na+/K+ uptake | 101.8 | 6 | 3.83 | 12.35 | RM3827-RM340 |
|  |  |  |  | *qNAKUP-3* | Na+/K+ uptake | 117.1 | 3 | 4.25 | 9.03 | RM6832-RM7389 |
|  |  |  |  |  |  |  |  |  |  |  |
| Zang et al. (2008) | IR64/Binam | BC_2_F_8_ Ils | 99 | qSST-2 | Score of salt toxicity of leaves | 131.1 | 2 | __ | __ | RM 250-RM 208 |
|  |  |  |  | qSST-3 | Score of salt toxicity of leaves | 15.7 | 3 | __ | __ | RM231-RM175 |
|  |  |  |  | qSST-8 | Score of salt toxicity of leaves | 8.5 | 8 | __ | __ | RM38-RM25 |
|  |  |  |  | qSDS-2a | survival days of seedlings | 3.3 | 2 | __ | __ | OSR17-RM211 |
|  |  |  |  | qSDS-2b | survival days of seedlings | 131.1 | 2 | __ | __ | RM530-RM250 |
|  |  |  |  | qSDS-3 | survival days of seedlings | 15.7 | 3 | __ | __ | RM231-RM175 |
|  |  |  |  | qSDS-8 | survival days of seedlings | 8.5 | 8 | __ | __ | RM38-RM25 |
|  |  |  |  | qSKC-1 | shoot K+ concentration | 58.5 | 1 | __ | __ | RM562-RM9 |
|  |  |  |  | qSKC-3 | shoot K+ concentration | 7.8 | 3 | __ | __ | RM81B-RM22 |
|  |  |  |  | qSKC-6 | shoot K+ concentration | 45.1 | 6 | __ | __ | RM50-RM539 |
|  |  |  |  | qSKC-11 | shoot K+ concentration | 41.7 | 11 | __ | __ | RM120-RM181 |
|  |  |  |  | qSNC-3 | shoot Na+ concentration | 15.5 | 3 | __ | __ | RM231-RM175 |
|  |  |  |  | qSNC-6 | shoot Na+ concentration | 78 | 6 | __ | __ | RM527-RM3 |
|  |  |  |  | qPh-1 | plant height | 31.9 | 1 | __ | __ | RM243-RM600 |
|  |  |  |  | qPh-4 | plant height | 82.1 | 4 | __ | __ | RM142-RM273 |
|  |  |  |  | qPh-5 | plant height | 1.2 | 5 | __ | __ | RM122-RM13 |
|  |  |  |  | qPh-7 | plant height | 117.4 | 7 | __ | __ | RM172-RM248 |
|  |  |  |  | qPh-8 | plant height | 89.9 | 8 | __ | __ | RM223-RM210 |
|  |  |  |  | qPh-9 | plant height | 57.5 | 9 | __ | __ | RM105-RM409 |
|  |  |  |  | qTN-1 | Tiller number | 58.5 | 1 | __ | __ | RM562-RM9 |
|  |  |  |  | qTN-2 | Tiller number | 35 | 2 | __ | __ | RM71-RM324 |
|  |  |  |  | qTN-5 | Tiller number | 1.2 | 5 | __ | __ | RM122-RM13 |
|  |  |  |  | qTN-6 | Tiller number | 125.4 | 6 | __ | __ | RM528-RM30 |
|  |  |  |  | qTN-8 | Tiller number | 80.4 | 8 | __ | __ | RM339-RM42 |
|  |  |  |  | qTN-9 | Tiller number | 58.8 | 9 | __ | __ | RM409-RM566 |
|  |  |  |  | qTN-10 | Tiller number | 29.8 | 10 | __ | __ | RM222-RM259 |
|  |  |  |  | qSFW-1 | freshweight of shoot | 32.3 | 1 | __ | __ | RM575-RM259 |
|  |  |  |  | qSFW-2 | freshweight of shoot | 35 | 2 | __ | __ | RM71-RM324 |
|  |  |  |  | qSFW-2 | freshweight of shoot | 118.5 | 2 | __ | __ | RM318-RM530 |
|  |  |  |  | qSFW-4 | freshweight of shoot | 2.8 | 4 | __ | __ | RM335-RM261 |
|  |  |  |  | qSFW-7 | freshweight of shoot | 63.5 | 7 | __ | __ | RM10-RM234 |
|  |  |  |  | qSFW-8 | freshweight of shoot | 52.2 | 8 | __ | __ | RM38-RM25 |
|  |  |  |  | qSFW-10 | freshweight of shoot | 54 | 10 | __ | __ | RM239-RM467 |
|  |  |  |  | qSFW-11 | freshweight of shoot | 0.8 | 11 | __ | __ | RM181-RM260 |
|  |  |  |  | qSFW-11 | freshweight of shoot | 85.7 | 11 | __ | __ | RM229-RM21 |
|  |  |  |  |  |  |  |  |  |  |  |
| Cheng et al.(2008) | TN1/CJ06 | DH | 120 | qRGE10 | Relative germination energy | 54 | 10 | <0.0001 | 6.59 | RM467–RM271 |
|  |  |  |  | qRGE11 | Relative germination energy | 10.5 | 11 | <0.01 | 4.15 | RM1812–RM5599 |
|  |  |  |  | qRGR7 | Relative germination rage | 83.2 | 7 | <0.01 | 4.72 | RM3826–RM1279 |
|  |  |  |  | qRSH4 | Relative seedling height | 29.2 | 4 | <0.001 | 8.21 | RM3471–RM307 |
|  |  |  |  | qRSH6 | Relative seedling height | 108.3 | 6 | <0.001 | 7.03 | RM454–RM528 |
|  |  |  |  | qRRL3 | Relative root length | 79.1 | 3 | <0.0001 | 15.1 | RM251–RM3280 |
|  |  |  |  | qRRL7 | Relative root length | 89.2 | 7 | <0.01 | 10.11 | RM3826–RM1279 |
|  |  |  |  | qRRN1 | Relative root number | 23.5 | 1 | <0.0001 | 11.78 | RM1–RM1195 |
|  |  |  |  | qRRN2 | Relative root number | 122.5 | 2 | <0.0001 | 15.8 | RM450–RM5472 |
|  |  |  |  | qRRN5 | Relative root number | 89.7 | 5 | <0.0001 | 9.08 | RM5642–RM6972 |
|  |  |  |  | qRVI3 | Relative vigor index | 43.6 | 3 | <0.0001 | 5.21 | RM3280–RM282 |
|  |  |  |  | qADG10 | Alkali damage rate in germination period | 33.4 | 10 | ,<0.01 | 4.79 | RM216–RM467 |
|  |  |  |  | qADS3 | Alkali damage rate at early seedling stage | 79.1 | 3 | <0.0001 | 14.85 | RM251–RM3280 |
|  |  |  |  | qADS7 | Alkali damage rate at early seedling stage | 83.2 | 7 | <0.0001 | 11.28 | RM3826–RM1279 |
|  |  |  |  |  |  |  |  |  |  |  |
| Kim et al. (2009) | Ilpumbyeo/Moroberekan | BC3F5-ILs | 117 | *qRDW-6* | Reduction of dry weight | 95.43 | 6 | 2.1 | 10.2 | RM7269 |
|  |  |  |  | *qRFW-6* | Reduction of fresh weight | 95.43 | 6 | 2.1 | 10.9 | RM7269 |
|  |  |  |  | *qRDW-7* | Reduction of dry weight | 75.9 | 7 | 3.4 | 13.6 | RM320-RM11 |
|  |  |  |  | *qRFW-7* | Reduction of fresh weight | 75.9 | 7 | 3.2 | 13.9 | RM320-RM11 |
|  |  |  |  | *qRLA-7* | Reduction of leaf area | 75.9 | 7 | 3 | 12.1 | RM320-RM11 |
|  |  |  |  | *qRSH-7* | Reduction of seedling height | 75.9 | 7 | 2.13 | 10.5 | RM320-RM11 |
|  |  |  |  |  |  |  |  |  |  |  |
| Ammar et al. (2009) | CSR27/ MI48 | F2&F3 families | 200 | qSIS-1.1 | Seedling salt injury score | 53.35 | 1 | 2.33 | 14.38 | RM572-RM294 |
|  |  |  |  | qNaSV-1.1 | Na+ in stem at vegetative stage | 53.35 | 1 | 6.62 | 52.27 | RM563-RM186 |
|  |  |  |  | qKSV-1.1 | K+ in stem at vegetative stage | 56.2 | 1 | 2.18 | 11.13 | E60275-RM3395 |
|  |  |  |  | qNa/KSV-1.1 | Na+/k+ Ratio in stem at vegetative stage | 53.35 | 1 | 2.79 | 48.54 | RM3395-RM281 |
|  |  |  |  | qClSV-1.1 | Cl^-^ in stem at vegetative stage | 53.35 | 1 | 6.45 | 55.72 | RM145-RM5699 |
|  |  |  |  | qNaLR-2.1 | Na+ in leaves at reproductive stage | 39.45 | 2 | 2.66 | 33.25 | RM563-RM186 |
|  |  |  |  | qNaSV-2.1 | Na+ in stem at vegetative stage | 39.45 | 2 | 6.57 | 49 | RM3395-RM281 |
|  |  |  |  | qNa/kSV-2.1 | Na+/k+ Ratio in stem at vegetative stage | 29 | 2 | 8.18 | 46.57 | RM572-RM294 |
|  |  |  |  | qNa/kSV-2.2 | Na+/k+ Ratio in stem at vegetative stage | 29 | 2 | 10.08 | 45.5 | RM145-RM5699 |
|  |  |  |  | qNa/kSV-2.3 | Na+/k+ Ratio in stem at vegetative stage | 39.45 | 2 | 9.7 | 42.88 | RM3395-RM281 |
|  |  |  |  | qClLR-2.1 | Cl^-^ in leaf at reproductive stage | 39.45 | 2 | 7.57 | 26.26 | RM563-RM186 |
|  |  |  |  | qClSV-2.1 | Cl^-^ in stem at vegetative stage | 39.45 | 2 | 5.89 | 42.5 | RM3395-RM281 |
|  |  |  |  | qNaLV-3.1 | Na+ in leaves at vegetative stage | 57.75 | 3 | 6.6 | 53.8 | RM294-RM23 |
|  |  |  |  | qNaLR-3.1 | Na+ in leaves at reproductive stage | 57.75 | 3 | 2.85 | 40.09 | RM563-RM186 |
|  |  |  |  | qKLV 3.1 | K+ in leaves at vegetative stage | 57.75 | 3 | 2.01 | 37.82 | RM3395-RM281 |
|  |  |  |  | qNa/kLR-3.1 | Na+/k+ Ratio in leaf at reproductive stage | 57.75 | 3 | 9.32 | 52.63 | RM572-RM294 |
|  |  |  |  | qNa/kSV-3.1 | Na+/k+ Ratio in stem at vegetative stage | 57.75 | 3 | 5.29 | 47.51 | RM3732-RM145 |
|  |  |  |  | qClLV-3.1 | Cl^-^  in leaf at vegetative stage | 57.75 | 3 | 7.78 | 48.51 | RM3732-RM145 |
|  |  |  |  | qNaLV-8.1 | Na+ in leaves at vegetative stage | 45.05 | 8 | 2.17 | 12.73 | RM145-RM5699 |
|  |  |  |  | qNaLV-8.2 | Na+ in leaves at vegetative stage | 86.4 | 8 | 4.78 | 55.18 | RM563-RM186 |
|  |  |  |  | qNaLR-8.1 | Na+ in leaves at reproductive stage | 86.4 | 8 | 3.95 | 47.59 | RM3395-RM281 |
|  |  |  |  | qNaSV-8.1 | Na+ in stem at vegetative stage | 86.4 | 8 | 4.72 | 53.63 | RM563-RM186 |
|  |  |  |  | qKLR-8.1 | K+ in leaves at reproductive stage | 86.4 | 8 | 2.82 | 36.49 | RM145-RM5699 |
|  |  |  |  | qNa/kLR-8.1 | Na+/k+ Ratio in leaf at reproductive stage | 86.4 | 8 | 6 | 51.03 | RM572-RM294 |
|  |  |  |  | qNa/kSV-8.1 | Na+/k+ Ratio in stem at vegetative stage | 86.4 | 8 | 4.84 | 51.78 | RM145-RM5699 |
|  |  |  |  |  |  |  |  |  |  |  |
| Sabouri et al. (2009) | Tarommahalli/Khazar | F2:F3 | 192 | *qSTR-6* | standard tolerance ranking | 101.8 | 6 | 17.51 | 17.25 | RM3727-RM340 |
|  |  |  |  | *qSTR-3a* | standard tolerance ranking | 48.4 | 3 | 13.44 | 16.15 | RM1022-RM6283 |
|  |  |  |  | *qSTR-3b* | standard tolerance ranking | 117.1 | 3 | 24.51 | 13.07 | RM6832-RM7389 |
|  |  |  |  | *qDM-3* | Dry mass of shoot | 48.4 | 3 | 20.5 | 20.9 | RM1022-RM6283 |
|  |  |  |  | *qDM-8* | Dry mass of shoot | 7.3 | 8 | 20.24 | 17.72 | RM4955-RM152 |
|  |  |  |  | *qNA-2a* | Na+ content in shoot | 81.3 | 2 | 12.59 | 10.55 | RM8264-RM262 |
|  |  |  |  | *qNA-2b* | Na+ content in shoot | 37.6 | 2 | 12.57 | 12.7 | RM7426-RM236 |
|  |  |  |  | *qNA-6* | Na+ content in shoot | 96.2 | 6 | 11.92 | 10.13 | RM3827-RM5371 |
|  |  |  |  | *qNA-3* | Na+ content in shoot | 117.1 | 3 | 16.34 | 10.92 | RM6832-RM7389 |
|  |  |  |  | *qK-6* | K+ content in shoot | 101.8 | 6 | 14.46 | 10.8 | RM3827-RM340 |
|  |  |  |  | *qK-5A* | K+ content in shoot | 102.6 | 5 | 17.16 | 15.58 | RM421-RM480 |
|  |  |  |  | *qK-5B* | K+ content in shoot | 94.5 | 5 | 12.35 | 9.7 | RM480-RM440 |
|  |  |  |  | *qNAK-6* | Na+/k+ Ratio | 101.8 | 6 | 16.68 | 12.35 | RM3827-RM340 |
|  |  |  |  | *qNAK-3* | Na+/k+ ratio | 117.1 | 3 | 18.52 | 9.03 | RM6832-RM7389 |
|  |  |  |  |  |  |  |  |  |  |  |
| Thomson et al. (2010) | R29/Pokkali | RILs | 140 | qSNC1 | Shoot Na+ concentration | 36 | 1 | 3.6 | 14 | RM1287-RM10793 |
|  |  |  |  | qSKC1 | Shoot K+ concentration | 41.2 | 1 | 3.2 | 13 | RM8094-RM10825 |
|  |  |  |  | qSNK1 | shoot Na-K Ratio | 41.2 | 1 | 7.6 | 27 | RM1287-RM10825 |
|  |  |  |  | qRKC1 | Root K+ concentration | 36 | 1 | 4 | 19 | RM1287-RM11300 |
|  |  |  |  | qRNK1 | Root Na-K Ratio | 41.2 | 1 | 5.5 | 21 | RM1287-RM10825 |
|  |  |  |  | qSUR1 | Seedling survival | 125.2 | 1 | 3.2 | 11 | RM7643-RM11874 |
|  |  |  |  | qCHL 1 | Leaf chlorophyll content | 125.2 | 1 | 3.6 | 27 | RM7643-RM11874 |
|  |  |  |  | qPH2 | Seedling height | 72.4 | 2 | 11.1 | 67 | RM13197-RM6318 |
|  |  |  |  | qRKC2 | Root K+ concentration | 72.4 | 2 | 3.8 | 36 | RM13197-RM6318 |
|  |  |  |  | qCHL2 | Leaf chlorophyll content | 72.4 | 2 | 11.3 | 56 | RM12713-RM6318 |
|  |  |  |  | qSUR2 | Seedling survival | 61.6 | 2 | 1.7 | 6 | RM300-RM13332 |
|  |  |  |  | qCHL 2 | Leaf chlorophyll content | 72.4 | 2 | 4.1 | 52 | RM13197-RM6318 |
|  |  |  |  | qCHL3 | Leaf chlorophyll content | 104.8 | 3 | 3.1 | 13 | RM5626-RM3867 |
|  |  |  |  | qSES3 | Initial SES tolerance score | 104.8 | 3 | 3.8 | 23 | RM6369-RM3867 |
|  |  |  |  | qCHL 3 | Leaf chlorophyll content | 104.8 | 3 | 3.5 | 30 | RM6329-RM3867 |
|  |  |  |  | qPH4 | Seedling height | 118.8 | 4 | 4.3 | 65 | RM17391-RM127 |
|  |  |  |  | qSES4 | Final SES tolerance score | 129.2 | 4 | 3.1 | 13 | RM3843-RM127 |
|  |  |  |  | qCHL4 | Leaf chlorophyll content | 129.2 | 4 | 3.9 | 25 | RM3843-RM127 |
|  |  |  |  | qRKC6 | Root K+ concentration | 81.2 | 6 | 3.9 | 12 | RM19840-RM20350 |
|  |  |  |  | qRNK6 | Root Na-K Ratio | 81.2 | 6 | 3.8 | 12 | RM19840-RM20350 |
|  |  |  |  | qSNK9 | shoot Na-K Ratio | 52.8 | 9 | 2.8 | 25 | RM296-RM7175 |
|  |  |  |  | qRNK9 | Root Na-K Ratio | 52.8 | 9 | 2.5 | 26 | RM296-RM7175 |
|  |  |  |  | qSES9 | Final SES tolerance score | 52.8 | 9 | 2.8 | 55 | RM296-RM7175 |
|  |  |  |  | qSES12 | Initial SES tolerance score | 50.8 | 12 | 3.4 | 15 | RM27933-RM17 |
|  |  |  |  | qSES12 | Final SES tolerance score | 50.8 | 12 | 5.5 | 47 | RM27933-RM17 |
|  |  |  |  | qSUR12 | Seedling survival | 50.8 | 12 | 4.2 | 38 | RM27933-RM17 |
|  |  |  |  | qCHL 12 | Leaf chlorophyll content | 50.8 | 12 | 3.7 | 15 | RM27933-RM17 |
|  |  |  |  |  |  |  |  |  |  |  |
| Pandit et al. (2010) | CSR 27/MI48 | F7 RILs | 216 | qKLV-1.1 | K+ concentration in leaves at vegetative stage | 191.21 | 1 | 2.94 | 7.39 | HvSSR01-24: RM572 |
|  |  |  |  | qNaSH-1.1 | Na+ concentration in straw at high salt stress | 265.81 | 1 | 3.17 | 8.53 | RM294: HvSSR01-37 |
|  |  |  |  | qKSH-1.1 | K+ concentration in straw at high salt stress | 290.11 | 1 | 4.43 | 11.07 | HvSSR01-40 :HvSSR01-44 |
|  |  |  |  | qNa/KSH-1.1 | Na+/K+ ratio in straw at high-salinity stress | 294.91 | 1 | 3.47 | 7.22 | HvSSR01-44: HvSSR01-46 |
|  |  |  |  | qNaSH-8.1 | Na+ concentration in straw at high salt stress | 90.51 | 8 | 4.22 | 14.05 | RM1235 : RM25 |
|  |  |  |  | qClLV-8.1a | Cl^-^ ion concentration in leaves at vegetative stage | 12.51 | 8 | 3.87 | 8.85 | HvSSR08-25: RM3395 |
|  |  |  |  | qCILV-8.1b | Cl^-^ ion concentration in leaves at vegetative stage | 131.91 | 8 | 3.3 | 7.37 | RM3395 : HvSSR08-35 |
|  |  |  |  | qSSISFH-8.1 | SSI for spikelet fertility at high salt conc | 127.91 | 8 | 4.17 | 8 | HvSSR08-25 : RM3395 |
|  |  |  |  | qNaSV-12.1 | Na+ concentration in stem at vegetative stage | 63.31 | 12 | 3.01 | 5.86 | HvSSR12-24 : RM1261 |
|  |  |  |  |  |  |  |  |  |  |  |
|  |  |  |  |  |  |  |  |  |  |  |
| Alam et al. (2011) | IR 29/ Pokkali | BC3F4 | 181 | qSES1.1 | SES score | **45.6** | 1 | 3.1 | 4 | SKC 1 |
|  |  |  |  | qSES1.2 | SES score | **48.8** | 1 | 12.47 | 18.42 | RM493 |
|  |  |  |  | qSES3.1 | SES score | **32.4** | 3 | 3.28 | 8 | RM5639 |
|  |  |  |  | qSES3.2 | SES score | **98.8** | 3 | 3.85 | 9 | RM5626 |
|  |  |  |  | qSES3.3 | SES score | **120.4** | 3 | 6.14 | 14 | RM3867 |
|  |  |  |  | qSES3.4 | SES score | **120.4** | 3 | 5.23 | 7.32 | RM3867 |
|  |  |  |  | qSES4 | SES score | **38.8** | 4 | 4.21 | 5.28 | RM6659 |
|  |  |  |  | qSES5 | SES score | **94.6** | 5 | 4.56 | 11 | RM163 |
|  |  |  |  | qSES6 | SES score | **82.4** | 6 | 5.19 | 13 | RM20224 |
|  |  |  |  | qSES10.1 | SES score | **26.6** | 10 | 5.72 | 14 | RM222 |
|  |  |  |  | qSES10.2 | SES score | **26.6** | 10 | 7.78 | 10.83 | RM222 |
|  |  |  |  | qSES11.1 | SES score | **22.6** | 11 | 6.39 | 15 | RM26063 |
|  |  |  |  | qSES11.2 | SES score | **97.8** | 11 | 7.05 | 10.01 | RM224 |
|  |  |  |  |  |  |  |  |  |  |  |
| Ahmadi and Fotokian (2011) | Tarome-Molaei*3/ Tiqing | BC2F5 BILs | 62 | qKr1.1 | Potassium in root ( K^+^R) | 12.1 | 1 | 3.5 | 17 | RM200-RM220 |
|  |  |  |  | qKr1.2 | Potassium in root ( K^+^R) | 124.55 | 1 | 7.8 | 30 | RM473A-RM128 |
|  |  |  |  | qNas1 | Sodium in root (Na^+^R) | 139.15 | 1 | 3.8 | 15 | RM128-RM212 |
|  |  |  |  | qNas/Ks1 | Na^+^/K^+^ Ratio in shoot | 78.85 | 1 | 4 | 19 | RM23-RM5 |
|  |  |  |  | qNar/Kr1 | Na^+^/K^+^ Ratio in root | 124.55 | 1 | 4 | 18.4 | RM473A-RM128 |
|  |  |  |  | qKr3 | Potassium in root ( K^+^R) | 90.05 | 3 | 4 | 14 | RM251-RM282 |
|  |  |  |  | qKs4 | Potassium in Shoot ( K^+^S) | 64.45 | 4 | 4.1 | 19 | RM261d-RM273 |
|  |  |  |  | qKr4 | Potassium in root ( K^+^R) | 122.05 | 4 | 3 | 9 | RM241-RM348 |
|  |  |  |  | qNar/Kr4 | Na^+^/K^+^ Ratio in root | 122.05 | 4 | 3 | 9 | RM241-RM348 |
|  |  |  |  | qKs5 | Potassium in Shoot ( K^+^S) | 41.1 | 5 | 5.7 | 22 | RM413-**RM**289 |
|  |  |  |  | qNar/Kr5 | Na^+^/K^+^ Ratio in root | 13.1 | 5 | 5 | 27.6 | RM122-RM413 |
|  |  |  |  | qNas6 | Sodium in root (Na^+^R) | 91.2 | 6 | 4 | 24 | RM3-RM528 |
|  |  |  |  | qKr8 | Potassium in root ( K^+^R) | 110.15 | 8 | 3.5 | 20 | RM149-RM264 |
|  |  |  |  | qNar/Kr8 | Na^+^/K^+^ Ratio in root | 110.15 | 8 | 3.7 | 16.7 | RM149-RM264 |
|  |  |  |  |  |  |  |  |  |  |  |
| Tian et al. (2011) | Teqing/Oryza rufipogon Accession | ILs | 87 | qSTS1 | salt tolerance score | 31.88 | 1 | 0.001 (p) | 11 | RM243 |
|  |  |  |  | qSTS2 | salt tolerance score | 48.87 | 2 | 0.005 (*p*) | 9 | RM301 |
|  |  |  |  | qSTS7 | salt tolerance score | 63.5 | 7 | 0.002 (*p*) | 10 | RM10 |
|  |  |  |  | qRRW3 | relative root dry weight | 134.84 | 3 | 0.007 (*p*) | 8 | RM422 |
|  |  |  |  | qRRW6 | relative root dry weight | 24.92 | 6 | 0.001 (*p*) | 17 | RM276 |
|  |  |  |  | qRRW7 | relative root dry weight | 78.33 | 7 | 0.000 (*p*) | 22 | RM560 |
|  |  |  |  | qRRW10 | relative root dry weight | 59.4 | 10 | 0.000 (*p*) | 26 | RM271 |
|  |  |  |  | qRSW6 | relative shoot dry weight | 24.92 | 6 | 0.006 (*p*) | 8 | RM276 |
|  |  |  |  | qRSW7 | relative shoot dry weight | 78.33 | 7 | 0.001 (*p*) | 11 | RM560 |
|  |  |  |  | qRSW9 | relative shoot dry weight | 90.88 | 9 | 0.009 (*p*) | 8 | RM205 |
|  |  |  |  | qRSW10 | relative shoot dry weight | 59.4 | 10 | 0.000 (*p*) | 19 | RM271 |
|  |  |  |  | qRTW6 | relative total dry weight | 24.92 | 6 | 0.002 (*p*) | 11 | RM276 |
|  |  |  |  | qRTW7 | relative total dry weight | 78.33 | 7 | 0.000 (*p*) | 14 | RM560 |
|  |  |  |  | qRTW9 | relative total dry weight | 90.88 | 9 | 0.007 (*p*) | 8 | RM205 |
|  |  |  |  | qRTW10 | relative total dry weight | 59.4 | 10 | 0.000 (*p*) | 22 | RM271 |
|  |  |  |  |  |  |  |  |  |  |  |
| Javed et al. (2011) | Shaheen Basmati,/Pokkali | F2:3 | 190 | qShT-7 | Seedling height | 87.5 | 7 | 0.0053 (*p)* | 5.51 | RM336 |
|  |  |  |  | qSFW-7 | shoot fresh weight | 102.6 | 7 | 0.0035 (*p)* | 5.27 - 5.91 | RM18 |
|  |  |  |  | qSFW-7 | shoot fresh weight | 87.5 | 7 | 0.0065 (*p)* | 5.27- 5.91 | RM336 |
|  |  |  |  | qSFW-11 | shoot fresh weight | 67.1 | 11 | 0.0072 (*p)* | 5.27 -5.91 | RM287 |
|  |  |  |  | qSDW-1 | shoot dry weight | 155.6 | 1 | 0.0055 (p*)* | 4.89 -5.41 | RM431 |
|  |  |  |  | qSDW-1 | shoot dry weight | 109.3 | 1 | 0.0095 (p*)* | 4.89 -5.41 | RM246 |
|  |  |  |  | qSDW-7 | shoot dry weight | 3.2 | 7 | 0.0092 (*p)* | 4.89 -5.41 | RM481 |
|  |  |  |  | qSNC-6 | Shoot Na+ content | 75.5 | 6 | 0.0007 (*p)* | 7.82 | RM541 |
|  |  |  |  | qSNC-8 | Shoot Na+ content | 97.9 | 8 | 0.0004 (*p)* | 8.31 | RM80 |
|  |  |  |  | qSKC-3 | Shoot K+ content | 35.3 | 3 | 0.0058 (*p)* | 5.47 | RM545 |
|  |  |  |  | qSKC-8 | Shoot K+ content | 97.9 | 8 | 0.0006 (*p)* | 7.82 | RM80 |
|  |  |  |  | qRSC-1a | Root Na+ content | 132.2 | 1 | 0.009-0.0005(*p)* | 5.63- 8.91 | RM212 |
|  |  |  |  | qRSC-1b | Root Na+ content | 19.3 | 1 | 0.009-0.0005(*p)* | 5.63- 8.91 | RM428 |
|  |  |  |  | qRSC-3 | Root Na+ content | 109.6 | 3 | 0.009-0.0005(*p)* | 5.63- 8.91 | RM135 |
|  |  |  |  | qRSC-4 | Root Na+ content | 107.4 | 4 | 0.009-0.0005(*p)* | 5.63- 8.91 | RM241 |
|  |  |  |  | qRSC-6 | Root Na+ content | 75.5 | 6 | 0.009-0.0005(*p)* | 5.63- 8.91 | RM541 |
|  |  |  |  | qRKC-9 | Root K+ content | 75.2 | 9 | 0.0001 *(p*) | 10.29 | RM242 |
|  |  |  |  | qNKR-9a | Na+/K+ Ratio | 75.2 | 9 | 0.0001 *(p*) | 5.37 | RM242 |
|  |  |  |  | qNKR-9b | Na+/K+ Ratio | 80.7 | 9 | 0.0097 *(p*) | 10.55 | RM201 |
|  |  |  |  | qSIS-3 | Salt Injury Score | 48.6 | 3 | 0.0085 *(p*) | 4.97- 9.34 | RM7 |
|  |  |  |  | qSIS-6 | Salt Injury Score | 75.5 | 6 | 0.0006 *(p*) | 4.97- 9.34 | RM541 |
|  |  |  |  | qSIS-8 | Salt Injury Score | 97.9 | 8 | 0.0001 *(p*) | 4.97- 9.34 | RM80 |
|  |  |  |  |  |  |  |  |  |  |  |
| Islam et al. (2011) | BRRI dhan40/IR61920-3B-22-2-1 | F2 | 300 | *SalTol*1-1 | salinity tolerance at seedling stage | 9.35 | 1 | 2.7 | 12.5 | RM8094-RM3412 |
|  |  |  |  | *SalTol*8-1 | salinity tolerance at seedling stage | 21.75 | 8 | 7 | 29 | RM25-RM210 |
|  |  |  |  | *SalTol*10-1 | salinity tolerance at seedling stage | 47.1 | 10 | 4.5 | 20.2 | RM25092-RM25519 |
|  |  |  |  |  |  |  |  |  |  |  |
| Wang et al. (2011) | IR26/Jiucaiqing | F2:9 RILs | 150 | qGP-2 | Germination percentage | 76.05 | 2 | 32 | 36.5 | RM8254–RM5804 |
|  |  |  |  | qGP-3 | Germination percentage | 25.85 | 3 | 10.9 | 11.3 | RM49–RM6712 |
|  |  |  |  | qIR-4 | Imbibition rate | 116.4 | 4 | 3.5 | 6.5 | RM3687–RM3306 |
|  |  |  |  | qIR-6 | Imbibition rate | 10.75 | 6 | 7 | 33.6 | RM276–RM5531 |
|  |  |  |  | qGP-7.2 | Germination percentage | 50.9 | 7 | 4 | 9.6 | RM5623–RM1132 |
|  |  |  |  | qIR-9 | Imbibition rate | 173.4 | 9 | 5.8 | 33.7 | RM2144–RM3320 |
|  |  |  |  | qGP-9 | Germination percentage | 61.25 | 9 | 26.6 | 43.7 | RM219–RM7048 |
|  |  |  |  |  |  |  |  |  |  |  |
| Wang et al.(2012) | IR 26/Jiucaiqing | F2:9 RILS | 150 | *qSH1.1* | Seedling height | 40.3 | 1 | 4.1 | 9.3 | RM294-RM11179 |
|  |  |  |  | *qSH1.3* | Seedling height | 158.9 | 1 | 4.2 | 14.7 | RM3482-RM3362 |
|  |  |  |  | *qSH12.1* | Seedling height | 93.8 | 12 | 3 | 11.9 | RM5609-RM7376 |
|  |  |  |  | *qSH12.2* | Seedling height | 93.8 | 12 | 4.6 | 17.2 | RM7376–RM6953 |
|  |  |  |  | *qDSW6.1* | Dry shoot weight | 66.3 | 6 | 4.1 | 20.1 | RM6818-RM6811 |
|  |  |  |  | *qDSW6.2* | Dry shoot weight | 114.4 | 6 | 6.9 | 23.9 | RM340-RM3509 |
|  |  |  |  | *qDSW12.1* | Dry shoot weight | 93.8 | 12 | 4.7 | 17.1 | RM5609-RM7376 |
|  |  |  |  | *qDRW6* | dry root weight | 28.7 | 6 | 3 | 7.8 | RM5531-RM3183 |
|  |  |  |  | *qDRW11* | dry root weight | 53.6 | 11 | 2.9 | 9.6 | RM6091-RM229 |
|  |  |  |  |  |  |  |  |  |  |  |
| Ghomi et al. (2013) | Gharib/Sepidroud | F2:F4 | 148 | qRDW-1 | Root dry weight | 202.35 | 1 | 2.94 | 0.26 | RM1268-RM8231 |
|  |  |  |  | qSTR-1 | standard tolerance ranking | 112.6 | 1 | 2.53 | 12.41 | RM8132-E36-M60-7 |
|  |  |  |  | qSKC-1 | shoot K concentration | 143.85 | 1 | 2.69 | 16.69 | E38-M59-4-RM1287 |
|  |  |  |  | qSDW-2 | shoot dry weight | 43.15 | 2 | 3.9 | 17.88 | RM279-RM6911 |
|  |  |  |  | qSKC-2 | shoot K concentration | 96.1 | 2 | 2.8 | 1.95 | RM262-RM7624 |
|  |  |  |  | qSFW-3 | shoot fresh weight | 20.75 | 3 | 2.8 | 0.02 | RM60-RM489 |
|  |  |  |  | qSDW-3 | shoot dry weight | 20.75 | 3 | 2.65 | 0.72 | RM60-RM489 |
|  |  |  |  | qBM-3 | Biomass | 20.75 | 3 | 2.78 | 3.9 | RM60-RM489 |
|  |  |  |  | qRFW-4a | Root fresh weight | 1.15 | 4 | 3.08 | 7.81 | RM8213-E36-M59-5 |
|  |  |  |  | qRFW-4b | Root fresh weight | 9.4 | 4 | 3.92 | 19.06 | E36-M59-5-E37-M60-3 |
|  |  |  |  | qSFW-4a | Shoot fresh weight | 84.8 | 4 | 2.54 | 16.13 | RM252-E37-M61-6 |
|  |  |  |  | qSFW-4b | Shoot fresh weight | 178.4 | 4 | 2.55 | 3.48 | E36-M60-10-E38-M61-4 |
|  |  |  |  | qRDW-4 | Root dry weight | 178.4 | 4 | 2.76 | 5.03 | E36-M60-10-E38-M61-4 |
|  |  |  |  | qSTR-4 | Standard tolerance ranking | 41.55 | 4 | 2.78 | 5.48 | E37-M61-9-RM1359 |
|  |  |  |  | qSFW-5a | Shoot fresh weight | 181.65 | 5 | 3.36 | 9.91 | E36-M59-10-RM440 |
|  |  |  |  | qSFW-5b | Shoot fresh weight | 203.4 | 5 | 3.91 | 22.89 | RM459-RM3800 |
|  |  |  |  | qSDW-5a | Shoot dry weight | 181.65 | 5 | 2.58 | 5.3 | E36-M59-10-RM440 |
|  |  |  |  | qSDW-5b | Shoot dry weight | 203.4 | 5 | 3.94 | 1.59 | RM459-RM3800 |
|  |  |  |  | qBM-5a | Biomass | 181.65 | 5 | 3.47 | 16.27 | E36-M59-10-RM440 |
|  |  |  |  | qBM-5b | Biomass | 203.4 | 5 | 3.13 | 15.3 | RM459-RM3800 |
|  |  |  |  | qSHL-5 | Shoot length | 116.65 | 5 | 3.64 | 19.57 | RM13-RM164 |
|  |  |  |  | qSNK-5 | Shoot Na/K ratio | 8.45 | 5 | 4.19 | 0.32 | RM6320-E38-M61-11 |
|  |  |  |  | qSFW-6 | Shoot fresh weight | 247 | 6 | 2.7 | 6.51 | RM340-E36-M61-3 |
|  |  |  |  | qBM-6 | Biomass | 229.9 | 6 | 3.34 | 6 | E36-M60-6-RM5371 |
|  |  |  |  | qSHL-6 | Shoot length | 93.35 | 6 | 2.65 | 14.63 | RM402-RM549 |
|  |  |  |  | qRDW-7 | Root dry weight | 11.55 | 7 | 2.81 | 14.6 | E38-M61-6-E37-M61-12 |
|  |  |  |  | qSTR-8 | Standard tolerance ranking | 84.2 | 8 | 2.88 | 19.66 | RM7027-RM8264 |
|  |  |  |  | qSNK-8 | Shoot Na/K ratio | 33.3 | 8 | 2.69 | 15.12 | RM3572-RM404 |
|  |  |  |  | qCHL-8 | Chlorophyll | 2.9 | 8 | 2.83 | 15.78 | E37-M61-7-RM152 |
|  |  |  |  | qSTR-9 | Standard tolerance ranking | 122.5 | 9 | 2.65 | 21.7 | E36-M61-2-RM257 |
|  |  |  |  | qRL-9 | Root length | 73.95 | 9 | 3.28 | 15.59 | RM219-RM7038 |
|  |  |  |  | qSHL-9 | Shoot length | 141.7 | 9 | 2.55 | 9.99 | E37-M60-13-E36-M60-1 |
|  |  |  |  | qSNC-9 | Shoot Na concentration | 233.05 | 9 | 3.002 | 2.14 | RM201-RM215 |
|  |  |  |  | qSNK-9 | Shoot Na/K ratio | 161.8 | 9 | 2.61 | 9.88 | RM288-RM278 |
|  |  |  |  | qSHL-10 | Shoot length | 116.25 | 10 | 2.87 | 4.13 | RM2863-E36-M61-13 |
|  |  |  |  | qSKC-10a | Shoot K concentration | 46.9 | 10 | 2.66 | 3.71 | RM5620-RM258 |
|  |  |  |  | qSKC-10b | Shoot K concentration | 62.15 | 10 | 2.54 | 29.71 | RM258-RM333 |
|  |  |  |  | qSTR-11 | Standard tolerance ranking | 46.25 | 11 | 2.96 | 1.8 | E36-M59-6-RM202 |
|  |  |  |  | qSTR-12 | Standard tolerance ranking | 73.95 | 12 | 2.55 | 2.14 | RM2935-E36-M60-4 |
|  |  |  |  | qSNC-12 | Shoot Na concentration | 141.1 | 12 | 3.35 | 10.66 | RM1337-RM519 |
|  |  |  |  | qSNK-12 | Shoot Na/K ratio | 141.1 | 12 | 3.91 | 9.38 | RM1337-RM519 |
|  |  |  |  |  |  |  |  |  |  |  |
| Mohammadi et al. (2013) | Sadri/FL478 | F2 | 232 | qPH1.1s | Plant height | 189.8 | 1 | 9.4 | 17 | RM246–RM431 |
|  |  |  |  | qPL1.1s | Panicle length | 189.8 | 1 | 3.5 | 6.8 | RM246–RM431 |
|  |  |  |  | qPL2.1s | Panicle length | 48.6 | 2 | 3.8 | 7.2 | RM174–RM424 |
|  |  |  |  | qFRSP2.1s | Number of fertile spikelets | 28.1 | 2 | 3.3 | 6.3 | RM423–RM174 |
|  |  |  |  | qGY2.1s | Grain yield per plant | 39.15 | 2 | 3.6 | 6.9 | RM555–RM324 |
|  |  |  |  | qSPFR2.1s | Spikelet fertility | 28.1 | 2 | 4.2 | 7.9 | RM423–RM174 |
|  |  |  |  | qPH3.1s | Plant height | 117.2 | 3 | 7.8 | 14.3 | RM487–RM130 |
|  |  |  |  | qPL3.1s | Panicle length | 147 | 3 | 4.3 | 8.2 | RM520–RM570 |
|  |  |  |  | qSTSP3.1s | Number of sterile spikelets | 65.65 | 3 | 4.8 | 9.2 | RM251–RM338 |
|  |  |  |  | qDTF4.1s | Days to flowering | 95.45 | 4 | 3.6 | 6.9 | RM119–RM470 |
|  |  |  |  | qPN4.1s | Number of panicles | 35.45 | 4 | 4.6 | 8.6 | RM335–RM119 |
|  |  |  |  | qSTW4.1s | Straw dry weight | 4 | 4 | 5.1 | 9.6 | RM551–RM518 |
|  |  |  |  | qFRSP4.1s | Number of fertile spikelets | 4 | 4 | 4.4 | 8.3 | RM551–RM518 |
|  |  |  |  | qTSP4.1s | Total spikelets number | 4 | 4 | 6.7 | 12.4 | RM551–RM518 |
|  |  |  |  | qGY4.1s | Grain yield per plant | 4 | 4 | 4.2 | 7.9 | RM551–RM518 |
|  |  |  |  | qPH5.1s | Plant height | 118.6 | 5 | 3.4 | 6.6 | RM421–RM538 |
|  |  |  |  | qSPFR5.1s | Spikelet fertility | 102.1 | 5 | 2.2 | 4.2 | RM440–RM421 |
|  |  |  |  | qTGW5.1s | 1000-grain weight | 14.6 | 5 | 4 | 7.6 | RM507–RM13 |
|  |  |  |  | qDTF6.1s | Days to flowering | 63.55 | 6 | 4.8 | 9.1 | RM539–RM20224 |
|  |  |  |  | qPN6.1s | Number of panicles | 85.55 | 6 | 4.9 | 9.2 | RM20224–RM528 |
|  |  |  |  | qFRSP6.1s | Number of fertile spikelets | 85.55 | 6 | 4.1 | 7.7 | RM20224–RM528 |
|  |  |  |  | qGY6.1s | Grain yield per plant | 85.55 | 6 | 4.4 | 8.4 | RM20224–RM528 |
|  |  |  |  | qTGW6.1s | 1000-grain weight | 85.55 | 6 | 3.2 | 6.2 | RM20224–RM528 |
|  |  |  |  | qPH7.1s | Plant height | 79.55 | 7 | 6.8 | 12.6 | RM560–RM351 |
|  |  |  |  | qSTW7.1s | Straw dry weight | 55.9 | 7 | 4.2 | 8 | RM445–RM11 |
|  |  |  |  | qSTSP7.1s | Number of sterile spikelets | 62.4 | 7 | 3.7 | 7.1 | RM432–RM560 |
|  |  |  |  | qTSP7.1s | Total spikelets number | 59.4 | 7 | 4.1 | 7.8 | RM445–RM11 |
|  |  |  |  | qSTW8.1s | Straw dry weight | 24.35 | 8 | 3.2 | 6.2 | RM407–RM310 |
|  |  |  |  | qGY8.1s | Grain yield per plant | 144.8 | 8 | 4.4 | 8.4 | RM80–RM281 |
|  |  |  |  | qTGW8.1s | 1000-grain weight | 144.8 | 8 | 18 | 30 | RM80–RM281 |
|  |  |  |  | qPN9.1s | Number of panicles | 92.35 | 9 | 4.1 | 7.9 | RM410–RM215 |
|  |  |  |  | qSTW9.1s | Straw dry weight | 92.35 | 9 | 3.3 | 6.4 | RM410–RM215 |
|  |  |  |  | qTSP9.1s | Total spikelets number | 76.2 | 9 | 3.6 | 6.9 | RM434–RM242 |
|  |  |  |  | qDTF10.1s | Days to flowering | 55.45 | 10 | 7.2 | 13.3 | RM271–RM484 |
|  |  |  |  | qFRSP10.1s | Number of fertile spikelets | 104.2 | 10 | 3.1 | 5.9 | RM484–RM590 |
|  |  |  |  | qSPFR10.1s | Spikelet fertility | 55.45 | 10 | 2.7 | 5.2 | RM271–RM484 |
|  |  |  |  | qTGW10.1s | 1000-grain weight | 55.45 | 10 | 9.7 | 17.6 | RM271–RM484 |
|  |  |  |  |  |  |  |  |  |  |  |
| Bimpong et al. (2013) | IR29×Hasawi | F5 RILS | 300 | qPH1.1 | Plant height | 162.6 | 1 | 4.657 | 13.8 | id1023892 - id1024836 |
|  |  |  |  | qPH1.2 | Plant height | 168.6 | 1 | 4.257 | 12.7 | id1024972 - id1025983 |
|  |  |  |  | qDW1.1 | Dry weight | 102.6 | 1 | 11.342 | 30.4 | id1012048 - id1014176 |
|  |  |  |  | qDW2.1 | Dry weight | 116.8 | 2 | 17.169 | 42.3 | id2009319 - id2012453 |
|  |  |  |  | qDW2.2 | Dry weight | 130.8 | 2 | 16.851 | 41.7 | id2014034 - id2016108 |
|  |  |  |  | qFW2.1 | Fresh weight | 68.8 | 2 | 3.487 | 10.6 | id2004774 - id2007526 |
|  |  |  |  | qDW6.1 | Dry weight | 108.5 | 6 | 3.604 | 10.9 | id6013529 - id6014475 |
|  |  |  |  |  |  |  |  |  |  |  |
| Mardani et al. (2014) | Gharib/Sepidroud | F2:F4 | 148 | qGR-1 | Germination rate | 160.75 | 1 | 3.05 | 18.31 | RM237:RM246 |
|  |  |  |  | qRL-1 | Radicle length | 112.6 | 1 | 3.69 | 15.17 | RM8132:E36-M60-7 |
|  |  |  |  | qCFW-1 | Coleoptile fresh weight | 143.85 | 1 | 4.1 | 18.19 | E38-M59-4:RM1287 |
|  |  |  |  | qCDW-1 | Coleoptile dry weight | 154.8 | 1 | 3.08 | 10.03 | RM1287:RM237 |
|  |  |  |  | qGR-3-a | Germination rate | 82 | 3 | 3.73 | 18.53 | E37-M59-11:RM6832 |
|  |  |  |  | qGR-3-b | Germination rate | 176.05 | 3 | 3.84 | 19.23 | E36-M61-11: E36-M61-11 |
|  |  |  |  | qPFW-3 | Plumule fresh weight | 93.2 | 3 | 3.13 | 10.7 | RM6832:RM5636 |
|  |  |  |  | qRDW-3 | Radicle dry weight | 111 | 3 | 3.52 | 17.27 | RM5626:RM135 |
|  |  |  |  | qGP-4 | Germination percentage | 1.15 | 4 | 3.14 | 15.65 | RM8213:E37-M60-3 |
|  |  |  |  | qCL-5 | Coleoptile length | 203.4 | 5 | 3.38 | 17.7 | RM459:RM305 |
|  |  |  |  | qRFW-6 | Radicle fresh weight | 135 | 6 | 4.29 | 19.76 | RM3330:RM7179 |
|  |  |  |  | qPDW-7 | Plumule dry weight | 41.15 | 7 | 3.14 | 21.9 | RM5711:E36-M59-1 |
|  |  |  |  | qPL-8 | Plumule length | 96 | 8 | 3.19 | 16.1 | RM8264:E38-M61-1 |
|  |  |  |  | qPFW-8 | Plumule fresh weight | 126.75 | 8 | 3.81 | 15.13 | E36-M59-7:E38-M61-1 |
|  |  |  |  | qCFW-8 | Coleoptile fresh weight | 60.55 | 8 | 4.48 | 19.1 | RM42:RM331 |
|  |  |  |  | qRFW-11-a | Radicle fresh weight | 21.7 | 11 | 3.54 | 21.6 | E37-M59-10:RM1812 |
|  |  |  |  | qRFW-11-b | Radicle fresh weight | 34.75 | 11 | 2.7 | 10.06 | RM1812:E36-M59-6 |
| Zheng et al. (2014) | Dongnong425/Changbai10 | BC2F2:3 | 190 | qSNC-1 | Shoot Na+ concentration | 86.3 | 1 | 2.87 | 12.4 | RM580 - RM9 |
|  |  |  |  | qSKC-1 | Shoot K+ concentration | 86.3 | 1 | 2.82 | 12.4 | RM580 - RM9 |
|  |  |  |  | qSES-2 | Visual tolerance score | 25.5 | 2 | 4.05 | 11.3 | RM1285 - RM423 |
|  |  |  |  | qSNC-2 | Shoot K+ concentration | 25.5 | 2 | 2.95 | 10.1 | RM1285 - RM423 |
|  |  |  |  | qSKC-2 | Shoot K+ concentration | 25.5 | 2 | 3.22 | 13.5 | RM1285 - RM423 |
|  |  |  |  | qSKC-5 | Shoot K+ concentration | 104.3 | 5 | 2.95 | 8.4 | RM459 - RM1271 |
|  |  |  |  | qRNC-6 | Root K+ concentration | 74.7 | 6 | 3.8 | 14.3 | RM1340 -RM20341 |
|  |  |  |  | qRKC-6 | Root K+ concentration | 95.1 | 6 | 2.83 | 10.1 | RM20404- RM528 |
|  |  |  |  | qSES-7 | Visual tolerance score | 19.5 | 7 | 3.12 | 8 | RM82 - RM180 |
|  |  |  |  | qRNC-7 | root sodium concentration | 97.5 | 7 | 2.58 | 6.5 | RM560 - RM346 |
|  |  |  |  | qRNC-9 | root sodium concentration | 74.3 | 9 | 4.13 | 16.6 | RM201 - RM215 |
|  |  |  |  | qSNC-11 | Shoot Na+ concentration | 92.3 | 11 | 3.83 | 9.3 | RM229 - RM224 |
|  |  |  |  | qSNC-12 | Shoot Na+ concentration | 79 | 12 | 3.22 | 18 | RM28033 - RM1310 |
|  |  |  |  |  |  |  |  |  |  |  |
|  |  |  |  |  |  |  |  |  |  |  |
| Calpit-Palao et al. (2015) | IR64/IR4630-22-2-5-1-3 | F2 | 201 | qSDW1.1 | Shoot dry weight | 112.75 | 1 | 6.08 | 13.1 | RM200-RM443 |
|  |  |  |  | qKC1.1 | % Potassium (K) | 5.15 | 1 | 4.47 | 1.37 | RM495-RM10115 |
|  |  |  |  | qKC1.2 | % Potassium (K) | 45.6 | 1 | 3.44 | 2.82 | RM11008- RM10825 |
|  |  |  |  | qKC1.3 | % Potassium (K) | 73.7 | 1 | 4.6 | 2.09 | RM312-RM5638 |
|  |  |  |  | qKC1.4 | % Potassium (K) | 107.5 | 1 | 3.53 | 3.4 | RM488-RM443 |
|  |  |  |  | qPF2.1 | Pollen fertility | 125.85 | 2 | 2.71 | 6.1 | RM497-RM3248 |
|  |  |  |  | qTN2.1 | Tiller number | 25.35 | 2 | 3.4 | 30.84 | RM279-RM424 |
|  |  |  |  | qSDW2.1 | Shoot dry weight | 19.45 | 2 | 3.14 | 8 | RM236-RM492 |
|  |  |  |  | qNaC2.1 | % Sodium (Na) | 76.05 | 2 | 3.01 | 8.13 | RM327-RM3570 |
|  |  |  |  | qKC2.1 | % Potassium (K) | 36.3 | 2 | 9.9 | 3.83 | RM492-RM424 |
|  |  |  |  | qKC2.2 | % Potassium (K) | 76.05 | 2 | 3.81 | 0.22 | RM327-RM13570 |
|  |  |  |  | qKC3.1 | % Potassium (K) | 17.5 | 3 | 5.09 | 3.95 | RM231-RM545 |
|  |  |  |  | qKC3.2 | % Potassium (K) | 28.05 | 3 | 6.53 | 2.93 | RM218-OSR16 |
|  |  |  |  | qPF4.1 | Pollen fertility | 34.35 | 4 | 10.44 | 1.03 | RM471-RM518 |
|  |  |  |  | qPH4.1 | Plant height | 31 | 4 | 4.13 | 76.02 | RM551-RM471 |
|  |  |  |  | qPH4.2 | Plant height | 108.35 | 4 | 4.11 | 9.1 | RM17319-RM273 |
|  |  |  |  | qNaC4.1 | % Sodium (Na) | 34.35 | 4 | 4.33 | 5.08 | RM518-RM471 |
|  |  |  |  | qKC4.1 | % Potassium (K) | 3.95 | 4 | 5.19 | 3.72 | RM551-RM518 |
|  |  |  |  | qKC4.2 | % Potassium (K) | 69.9 | 4 | 10.84 | 6.23 | RM471-RM3558 |
|  |  |  |  | qKC4.3 | % Potassium (K) | 119.6 | 4 | 4.64 | 0.51 | RM273-RM280 |
|  |  |  |  | qNaK4.1 | Na-K ratio | 97.55 | 4 | 4.46 | 1.78 | RM252-RM470 |
|  |  |  |  | qPL5.1 | Panicle length | 4.15 | 5 | 3.85 | 5.05 | RM122-RM3345 |
|  |  |  |  | qPF6.1 | Pollen fertility | 58.1 | 6 | 11.09 | 2.71 | RM19715-RM3628 |
|  |  |  |  | qKC6.1 | % Potassium (K) | 3.7 | 6 | 4.16 | 1.34 | RM508-RM589 |
|  |  |  |  | qKC6.2 | % Potassium (K) | 95.85 | 6 | 3.6 | 1.85 | RM3628-RM528 |
|  |  |  |  | qPH7.2 | Plant height | 103.85 | 7 | 2.97 | 7.09 | RM429-RM248 |
|  |  |  |  | qNaC7.1 | % Sodium (Na) | 49.8 | 7 | 3.74 | 8.2 | RM125-RM455 |
|  |  |  |  | qKC7.1 | % Potassium (K) | 47.55 | 7 | 11.4 | 9.76 | RM125-RM11 |
|  |  |  |  | qPF8.1 | Pollen fertility | 41.9 | 8 | 15.13 | 2.31 | RM152-RM223 |
|  |  |  |  | qPL8.1 | Panicle length | 41.9 | 8 | 7.79 | 13.01 | RM152-RM223 |
|  |  |  |  | qTN8.1 | Tiller number | 41.9 | 8 | 2.7 | 6.9 | RM152-RM210 |
|  |  |  |  | qSDW8.1 | Shoot dry weight | 102.65 | 8 | 4.07 | 11.56 | RM210-RM458 |
|  |  |  |  | qRDW8.1 | Root dry weight | 102.65 | 8 | 11.5 | 26.48 | RM210-RM458 |
|  |  |  |  | qKC8.1 | % Potassium (K) | 41.9 | 8 | 15.31 | 5.89 | RM152-RM223 |
|  |  |  |  | qNaK8.1 | Na-K ratio | 102.65 | 8 | 12.13 | 1.07 | RM210-RM458 |
|  |  |  |  | qKC9.1 | % Potassium (K) | 72.3 | 9 | 2.65 | 1.36 | RM257-RM6643 |
|  |  |  |  | qKC10.1 | % Potassium (K) | 61.1 | 10 | 5.1 | 4.68 | RM271-RM304 |
|  |  |  |  | qNaK10.1 | Na-K ratio | 61.1 | 10 | 2.73 | 10.16 | RM271-RM304 |
|  |  |  |  | qRDW11.1 | Root dry weight | 75.1 | 11 | 2.97 | 7.69 | RM229-RM21 |
|  |  |  |  | qSGW12.1 | Single-grain weight | 56.9 | 12 | 6.2 | 13.4 | RM511-RM313 |
|  |  |  |  |  |  |  |  |  |  |  |
| Hossain et al. (2015) | Pusa Basmati 1/Cheriviruppu | F2 | 218 | qPH1.1 | Plant height | 133.65 | 1 | 20.8 | 48.7 | RM128-RM472 |
|  |  |  |  | qPH1.1 | Plant height | 133.65 | 1 | 22.3 | 47.1 | RM128-RM472 |
|  |  |  |  | qPL1.2 | Panicle length | 133.65 | 1 | 3.2 | 4.2 | RM128-RM472 |
|  |  |  |  | qPL1.2 | Panicle length | 133.65 | 1 | 4.9 | 6.3 | RM128-RM472 |
|  |  |  |  | qPF1.4 | Pollen fertility | 116.45 | 1 | 4.1 | 13.4 | RM246-RM6648 |
|  |  |  |  | qPF1.5 | Pollen fertility | 133.65 | 1 | 4.4 | 13.7 | RM128-RM472 |
|  |  |  |  | qNa1.6 | Sodium Concentration | 147.55 | 1 | 3.3 | 11.1 | RM7250-3482 |
|  |  |  |  | qNa1.7 | Sodium Concentration | 119.25 | 1 | 4.5 | 13.5 | RM1349-RM7250 |
|  |  |  |  | qNaKR1.8 | Na/K Ratio | 122 | 1 | 3.8 | 11 | RM237-RM7250 |
|  |  |  |  | qGY2.1 | Grain Yield | 13.5 | 2 | 7.9 | 12.2 | RM154-RM1313 |
|  |  |  |  | qGY3.1 | Grain Yield | 73.5 | 3 | 7.2 | 3.8 | RM3297-RM5626 |
|  |  |  |  | qPH4.1 | Plant height | 123.05 | 4 | 3.1 | 7.6 | RM17391-RM567 |
|  |  |  |  | qPH4.1 | Plant height | 123.05 | 4 | 3.8 | 7.2 | RM17391-RM567 |
|  |  |  |  | qPH7.1 | Plant height | 6.85 | 7 | 4.8 | 22.5 | RM51-RM1243 |
|  |  |  |  | qTN7.2 | Tiller number | 6.85 | 7 | 4 | 16.3 | RM51-RM1243 |
|  |  |  |  | qTN7.3 | Tiller number | 43.55 | 7 | 3.3 | 12 | RM180-RM7110 |
|  |  |  |  | qPL7.4 | Panicle length | 32.45 | 7 | 8.4 | 30 | RM180-RM3635 |
|  |  |  |  | qPL7.4 | Panicle length | 32.45 | 7 | 11.4 | 35.1 | RM180-RM3635 |
|  |  |  |  | qTN8.1 | Tiller number | 10.75 | 8 | 3.1 | 14.4 | RM6369-RM547 |
|  |  |  |  | qTN8.1 | Tiller number | 51.45 | 8 | 6.8 | 11.3 | RM3215-RM44 |
|  |  |  |  | qBM8.2 | Biomass | 51.45 | 8 | 3.7 | 17.9 | RM3215-RM44 |
|  |  |  |  | qPF10.1 | Pollen fertility | 55.25 | 10 | 3 | 12.8 | RM6142-RM181 |
|  |  |  |  | qPF10.2 | Pollen fertility | 7.3 | 10 | 3.2 | 8.6 | RM474-RM7515 |
|  |  |  |  | qGY12.1 | Grain Yield | 83.8 | 12 | 5.9 | 8.1 | RM519-RM1103 |
|  |  |  |  |  |  |  |  |  |  |  |
| Qui (2015) | Ce258/IR75862-206- | BC1F10 | 400 | qSST1 | score of salt toxicity | 117.85 | 1 | 8.79 | 11.13 | RM3143–RM443 |
|  | 2-8-3-B-B-B ; Zhongguangxiang 1/IR75862-206- |  |  |  |  |  |  |  |  |  |
|  | 2-8-3-B-B-B |  |  |  |  |  |  |  |  |  |
|  |  |  |  | qSKC1.2 | shoot K+ concentration | 141.3 | 1 | 3.04 | 8.65 | RM128–RM302 |
|  |  |  |  | qSKC1.1 | shoot K+ concentration | 110.8 | 1 | 4.7 | 9.07 | RM488–RM473 |
|  |  |  |  | qSST2 | score of salt toxicity | 32.1 | 2 | 2.64 | 5.43 | RM211–RM71 |
|  |  |  |  | qDSS2.1 | days of seedling survival | 3.45 | 2 | 6.16 | 9.41 | RM109–RM110 |
|  |  |  |  | qDSS3 | days of seedling survival | 46.6 | 3 | 2.8 | 5.13 | RM489–RM7 |
|  |  |  |  | qSNC3 | shoot Na+ concentration | 113.15 | 3 | 2.65 | 9.31 | RM282–RM156 |
|  |  |  |  | qSKC3 | shoot K+ concentration | 91.15 | 3 | 2.83 | 7.54 | RM5488–RM282 |
|  |  |  |  | qSST5 | score of salt toxicity | 98.95 | 5 | 9.08 | 13.32 | RM161–RM3476 |
|  |  |  |  | qSNC5 | shoot Na+ concentration | 13.35 | 5 | 3.54 | 8.93 | RM153–RM413 |
|  |  |  |  | qSST7 | score of salt toxicity | 75.15 | 7 | 4.32 | 8.16 | RM3691–RM1132 |
|  |  |  |  | qSST9 | score of salt toxicity | 100.05 | 9 | 5.34 | 9.34 | OSR28–RM225 |
|  |  |  |  | qDSS9 | days of seedling survival | 83.9 | 9 | 2.75 | 5.31 | RM160–OSR28 |
|  |  |  |  | qSKC10 | shoot K+ concentration | 36 | 10 | 5.43 | 10.44 | RM311–RM467 |
|  |  |  |  | qDSS11 | days of seedling survival | 32.7 | 11 | 2.5 | 7.39 | RM332–RM167 |
|  |  |  |  | qSST11 | score of salt toxicity | 32.7 | 11 | 10.04 | 13.75 | RM332–RM167 |
|  |  |  |  | qDSS11 | days of seedling survival | 32.7 | 11 | 8.01 | 12.51 | RM332–RM167 |
|  |  |  |  | qSKC11 | shoot K+ concentration | 48.95 | 11 | 3.82 | 6.31 | RM441–RM202 |
|  |  |  |  |  |  |  |  |  |  |  |
| Gimhani et al. (2016) | Bg352/At354 | F5 RILs | 281 | qSNK 1 | shoot Na+/K+ ratio | 90.8 | 1 | 5.03 | 21.8 | id1012784-779469 |
|  |  |  |  | qSNC1 | Shoot Na+ concentration | 90.8 | 1 | 4.03 | 17.9 | id1012784-779469 |
|  |  |  |  | qSKC1 | Shoot K+ concentration | 42.8 | 1 | 4.3 | 19 | 311989-328663 |
|  |  |  |  | qSSI1 | Salinity survival index | 90.8 | 1 | 4.36 | 19.2 | id1012784-779469 |
|  |  |  |  | qSL1 | Shoot length | 90.8 | 1 | 4.65 | 20.4 | id1012784-779469 |
|  |  |  |  | qSNK 2 | shoot Na+/K+ ratio | 134.6 | 2 | 5.77 | 24.6 | 2422788-2437583 |
|  |  |  |  | qSNC2 | Shoot Na+ concentration | 134.6 | 2 | 4.52 | 19.9 | 2422788-2437583 |
|  |  |  |  | qSKC2 | Shoot K+ concentration | 134.6 | 2 | 4.41 | 19.4 | 2422788-2437583 |
|  |  |  |  | qSSI2 | Salinity survival index | 134.6 | 2 | 7.48 | 30.7 | 2422788-2437583 |
|  |  |  |  | qPDS2 | Percentage damage of shoot | 134.6 | 2 | 7.57 | 31 | 2422788-2437583 |
|  |  |  |  | qSL2 | Shoot length | 134.6 | 2 | 4.79 | 20.9 | 2422788-2437583 |
|  |  |  |  | qSFW2 | Shoot fresh weight | 134.6 | 2 | 9.3 | 36.6 | 2422788-2437583 |
|  |  |  |  | qRFW2 | Root fresh weight | 134.6 | 2 | 7.02 | 29.1 | 2422788-2437583 |
|  |  |  |  | qSDW2 | Shoot dry weight | 134.6 | 2 | 8.5 | 34 | 2422788-2437583 |
|  |  |  |  | qRDW2 | Root dry weight | 134.6 | 2 | 5.73 | 24.5 | 2422788-2437583 |
|  |  |  |  | qSNK 3 | shoot Na+/K+ ratio | 140.3 | 3 | 5.46 | 23.5 | 3528886-id3017899 |
|  |  |  |  | qSNC3 | Shoot Na+ concentration | 140.3 | 3 | 4.95 | 21.5 | 3528886-id3017899 |
|  |  |  |  | qSKC3 | Shoot K+ concentration | 114.3 | 3 | 4.05 | 18 | id3013325-3382804 |
|  |  |  |  | qSSI3 | Salinity survival index | 142.3 | 3 | 5.04 | 21.9 | 3528886-id3017899 |
|  |  |  |  | qPDS3 | Percentage damage of shoot | 142.3 | 3 | 4.42 | 19.5 | 3528886-id3017899 |
|  |  |  |  | qSL3 | Shoot length | 140.3 | 3 | 3.99 | 17.8 | 3528886-id3017899 |
|  |  |  |  | qSFW3.1 | Shoot fresh weight | 84.3 | 3 | 3.03 | 13.8 | id3009515-id3010402 |
|  |  |  |  | qSFW3.2 | Shoot fresh weight | 140.3 | 3 | 4.66 | 20.4 | 3528886-id3017899 |
|  |  |  |  | qRFW3 | Root fresh weight | 142.3 | 3 | 4.46 | 19.6 | 3528886-id3017899 |
|  |  |  |  | qSDW3 | Shoot dry weight | 140.3 | 3 | 5.96 | 25.3 | 3528886-id3017899 |
|  |  |  |  | qSNK 4.1 | shoot Na+/K+ ratio | 78.1 | 4 | 3.12 | 14.2 | 4355198-4384860 |
|  |  |  |  | qSNK 4.2 | shoot Na+/K+ ratio | 104.1 | 4 | 3.05 | 13.9 | 4543652-4572241 |
|  |  |  |  | qSNC4 | Shoot Na+ concentration | 4.1 | 4 | 4.26 | 18.8 | 3610354-3623485 |
|  |  |  |  | qSKC4 | Shoot K+ concentration | 78.1 | 4 | 2.7 | 12.4 | 4355198-4384860 |
|  |  |  |  | qSSI4.1 | Salinity survival index | 84.1 | 4 | 3.41 | 15.4 | 4412114-4458054 |
|  |  |  |  | qSSI4.2 | Salinity survival index | 104.1 | 4 | 7.56 | 31 | 4543652-4572241 |
|  |  |  |  | qPDS4 | Percentage damage of shoot | 78.1 | 4 | 5.11 | 22.1 | 4355198-4384860 |
|  |  |  |  | qSFW4 | Shoot fresh weight | 4.1 | 4 | 4.78 | 20.9 | 3610354-3623485 |
|  |  |  |  | qRFW4 | Root fresh weight | 4.1 | 4 | 5.92 | 25.2 | 3610354-3623485 |
|  |  |  |  | qSDW4 | Shoot dry weight | 104.1 | 4 | 7.74 | 31.5 | 4543652-4572241 |
|  |  |  |  | qRDW4.1 | Root dry weight | 4.1 | 4 | 5.82 | 24.8 | 3610354-3623485 |
|  |  |  |  | qRDW4.2 | Root dry weight | 82.1 | 4 | 4.16 | 18.4 | 4384951-4412114 |
|  |  |  |  | qSNC5 | Shoot Na+ concentration | 80.7 | 5 | 3.14 | 14.2 | 5525279-id5008590 |
|  |  |  |  | qSSI5 | Salinity survival index | 80.7 | 5 | 3.92 | 17.5 | 5525279-id5008590 |
|  |  |  |  | qPDS5 | Percentage damage of shoot | 80.7 | 5 | 3.39 | 15.3 | 5525279-id5008590 |
|  |  |  |  | qSFW5 | Shoot fresh weight | 80.7 | 5 | 2.95 | 13.5 | 5525279-id5008590 |
|  |  |  |  | qSDW5 | Shoot dry weight | 80.7 | 5 | 3.21 | 14.5 | 5525279-id5008590 |
|  |  |  |  | qRDW5 | Root dry weight | 80.7 | 5 | 4.57 | 20.1 | 5525279-id5008590 |
|  |  |  |  | qSSI6 | Salinity survival index | 46.7 | 6 | 3.53 | 15.9 | 6178909-6228054 |
|  |  |  |  | qPDS6 | Percentage damage of shoot | 46.7 | 6 | 3.87 | 17.3 | 6178909-6228054 |
|  |  |  |  | qRL6 | Root length | 110.7 | 6 | 3.23 | 14.6 | 6855146-6873320 |
|  |  |  |  | qSFW7 | Shoot fresh weight | 15 | 7 | 2.92 | 13.3 | 7043229-7072228 |
|  |  |  |  | qRFW7 | Root fresh weight | 15 | 7 | 6.79 | 28.3 | 7043229-c7p5071487 |
|  |  |  |  | qSDW7 | Shoot dry weight | 15 | 7 | 4.15 | 18.4 | 7043229-7072228 |
|  |  |  |  | qRDW7 | Root dry weight | 15 | 7 | 2.72 | 12.5 | 7043229-7072228 |
|  |  |  |  | qSL8 | Shoot length | 101 | 8 | 2.73 | 12.5 | 8964581-8983572 |
|  |  |  |  | qSFW8 | Shoot fresh weight | 95 | 8 | 2.95 | 13.5 | 8926819-9008641 |
|  |  |  |  | qRFW8 | Root fresh weight | 111 | 8 | 5.03 | 21.8 | 8990744-9049928 |
|  |  |  |  | qSFW9 | Shoot fresh weight | 87.9 | 9 | 3.6 | 16.2 | 9859122-9880778 |
|  |  |  |  | qRFW9 | Root fresh weight | 81.9 | 9 | 3.03 | 13.8 | 9833069-9852552 |
|  |  |  |  | qRDW9 | Root dry weight | 87.9 | 9 | 3.82 | 17 | 9859122-9880778 |
|  |  |  |  | qSNK 10 | shoot Na+/K+ ratio | 2.5 | 10 | 7.65 | 31.2 | 9898598-id10000153 |
|  |  |  |  | qSNC10 | Shoot Na+ concentration | 2.5 | 10 | 8.47 | 34 | 9898598-id10000153 |
|  |  |  |  | qSKC10 | Shoot K+ concentration | 0.5 | 10 | 5.54 | 23.8 | 13069784-9922981 |
|  |  |  |  | qSSI10 | Salinity survival index | 2.5 | 10 | 9.78 | 38.1 | 9898598-id10000153 |
|  |  |  |  | qPDS10 | Percentage damage of shoot | 2.5 | 10 | 10.92 | 41.4 | 9898598-id10000153 |
|  |  |  |  | qSL10 | Shoot length | 0.5 | 10 | 7.79 | 31.7 | 13069784-9922981 |
|  |  |  |  | qSFW10 | Shoot fresh weight | 2.5 | 10 | 12.86 | 46.7 | 9898598-id10000153 |
|  |  |  |  | qRFW10 | Root fresh weight | 2.5 | 10 | 11.57 | 43.3 | 9898598-id10000153 |
|  |  |  |  | qSDW10 | Shoot dry weight | 2.5 | 10 | 12.31 | 45.3 | 9898598-id10000153 |
|  |  |  |  | qRDW10 | Root dry weight | 2.5 | 10 | 7.82 | 31.8 | 9898598-id10000153 |
|  |  |  |  | qSSI11 | Salinity survival index | 93.7 | 11 | 4.37 | 19.3 | 11756589-11789220 |
|  |  |  |  | qPDS11 | Percentage damage of shoot | 93.7 | 11 | 4.84 | 21.1 | 11756589-11789220 |
|  |  |  |  | qSFW11.1 | Shoot fresh weight | 93.7 | 11 | 5.18 | 22.4 | 11756589-11789220 |
|  |  |  |  | qSFW11.2 | Shoot fresh weight | 103.7 | 11 | 3.72 | 16.7 | c11p25591958-11887397 |
|  |  |  |  | qRFW11 | Root fresh weight | 93.7 | 11 | 4.63 | 20.3 | 11756589-11789220 |
|  |  |  |  | qSDW11.1 | Shoot dry weight | 93.7 | 11 | 6.55 | 37.4 | 11756589-11789220 |
|  |  |  |  | qSDW11.2 | Shoot dry weight | 103.7 | 11 | 5 | 21.7 | c11p25591958-11887397 |
|  |  |  |  | qRDW11 | Root dry weight | 5.7 | 11 | 4.18 | 18.5 | 10831672->5cM |
|  |  |  |  | qSNK 12 | shoot Na+/K+ ratio | 107.8 | 12 | 4.44 | 19.6 | 13048465-13069784 |
|  |  |  |  | qSNC12 | Shoot Na+ concentration | 107.8 | 12 | 4.75 | 20.8 | 13048465-13069784 |
|  |  |  |  | qSSI12 | Salinity survival index | 107.8 | 12 | 4.96 | 21.6 | 13048465-13069784 |
|  |  |  |  | qPDS12 | Percentage damage of shoot | 107.8 | 12 | 5.04 | 21.9 | 13048465-13069784 |
|  |  |  |  | qSL12 | Shoot length | 107.8 | 12 | 3.38 | 15.2 | 13048465-13069784 |
|  |  |  |  | qSFW12 | Shoot fresh weight | 107.8 | 12 | 6.74 | 28.1 | 13048465-13069784 |
|  |  |  |  | qRFW12 | Root fresh weight | 107.8 | 12 | 6.33 | 26.7 | 13048465-13069784 |
|  |  |  |  | qSDW12 | Shoot dry weight | 107.8 | 12 | 6.69 | 27.9 | 13048465-13069784 |
|  |  |  |  | qRDW12 | Root dry weight | 107.8 | 12 | 6.07 | 25.7 | 13048465-13069784 |
|  |  |  |  |  |  |  |  |  |  |  |
| De Leon et al.(2016) | Bengal/ Pokkali | F6 RILs | 187 | qK1.8 | K+ concentration | 63.35 | 1 | 5.79 | 13.65 | S1_8656025-S1_8901503 |
|  |  |  |  | qK1.11 | K+ concentration | 71.13 | 1 | 5.93 | 13.66 | S1_11529325-S1_11581799 |
|  |  |  |  | qK1.38 | K+ concentration | 173.17 | 1 | 3.51 | 8.3 | S1_38794029 - S1_39047133 |
|  |  |  |  | qK1.11 | K+ concentration | 71.13 | 1 | 7.74 | 16.08 | S1_11529325-S1_11581799 |
|  |  |  |  | qK1.38 | K+ concentration | 173.17 | 1 | 5.38 | 10.71 | S1_38794029- S1_39047133 |
|  |  |  |  | qNaK1.11 | NaK ratio | 71.13 | 1 | 4.15 | 9.83 | S1_11529325 -S1_11581799 |
|  |  |  |  | qNaK1.11 | NaK ratio | 71.13 | 1 | 2.64 | 5.66 | S1_11529325- S1_11581799 |
|  |  |  |  | qSHL1.1 | Shoot length | 11.17 | 1 | 2.04 | 5.03 | S1_1708228 -S1_1747144 |
|  |  |  |  | qSHL1.7a | Shoot length | 47.93 | 1 | 3.93 | 9.26 | S1_7259818 -S1_7296346 |
|  |  |  |  | qSHL1.38 | Shoot length | 168.81 | 1 | 25.35 | 48.03 | S1_38286772 - S1_38611845 |
|  |  |  |  | qSHL1.7b | Shoot length | 50.14 | 1 | 6.27 | 5.86 | S1_7520182 - S1_7569628 |
|  |  |  |  | qSHL1.38 | Shoot length | 168.81 | 1 | 36.91 | 51.64 | S1_38286772 - S1_38611845 |
|  |  |  |  | qRTL1.26 | Root length | 121.03 | 1 | 2.73 | 6.52 | S1_26421289 - S1_26447134 |
|  |  |  |  | qRTL1.22 | Root length | 102.05 | 1 | 2.27 | 3.54 | S1_22666852 - S1_22677418 |
|  |  |  |  | qRTL1.26 | Root length | 121.03 | 1 | 2.18 | 3.41 | S1_26421289 - S1_26447134 |
|  |  |  |  | qDWT1.21 | Dry weight | 97.13 | 1 | 2.34 | 5.6 | S1_21707357-S1_21733437 |
|  |  |  |  | qDWT1.40 | Dry weight | 185.08 | 1 | 2.07 | 3.13 | S1_40372283 - S1_40412316 |
|  |  |  |  | qSRR1.7 | Shoot-root ratio | 50.14 | 1 | 3.79 | 9.09 | S1_7520182 - S1_7569628 |
|  |  |  |  | qSRR1.29 | Shoot-root ratio | 135.07 | 1 | 3.12 | 7.42 | S1_29561423 - S1_29568978 |
|  |  |  |  | qSRR1.36 | Shoot-root ratio | 159.02 | 1 | 5.84 | 13.42 | S1_36158467 - S1_36189206 |
|  |  |  |  | qSRR1.382 | Shoot-root ratio | 168.81 | 1 | 10.31 | 23.01 | S1_38286772 -S1_38611845 |
|  |  |  |  | qSRR1.7 | Shoot-root ratio | 50.14 | 1 | 6.93 | 8.73 | S1_7520182- S1_7569628 |
|  |  |  |  | qSRR1.386 | Shoot-root ratio | 171.51 | 1 | 15.64 | 22.43 | S1_38636497 - S1_38768787 |
|  |  |  |  | qNa2.7 | Na+ concentration | 47.9 | 2 | 2.3 | 5.55 | S2_7769844 -S2_7939496 |
|  |  |  |  | qNa2.7 | Na+ concentration | 47.9 | 2 | 2.3 | 5.55 | S2_7769844 -S2_7939496 |
|  |  |  |  | qSIS2.8 | Salt injury score | 50.7 | 2 | 3.54 | 8.58 | S2_8730258 - S2_8927908 |
|  |  |  |  | qSIS2.19 | Salt injury score | 81.07 | 2 | 3.21 | 7.66 | S2_19331684 - S2_19454952 |
|  |  |  |  | qSIS2.28 | Salt injury score | 130.89 | 2 | 2.64 | 6.37 | S2_28239596 -S2_28274467 |
|  |  |  |  | qCHL2.20 | Chlorophyll content | 86.1 | 2 | 3.69 | 7.44 | S2_20258450 -S2_20346560 |
|  |  |  |  | qCHL2.30 | Chlorophyll content | 142.77 | 2 | 2.34 | 4.69 | S2_30353435 - S2_30402468 |
|  |  |  |  | qSHL2.18 | Shoot length | 76.76 | 2 | 3 | 2.71 | S2_18806154- S2_18937362 |
|  |  |  |  | qRTL2.24 | Root length | 114.1 | 2 | 4.14 | 9.72 | S2_24961302 - S2_24961342 |
|  |  |  |  | qRTL2.26 | Root length | 119.92 | 2 | 4.21 | 9.91 | S2_26028043 -S2_26070191 |
|  |  |  |  | qRTL2.33 | Root length | 160.11 | 2 | 3.94 | 9.5 | S2_33573567 -S2_33614297 |
|  |  |  |  | qSRR2.28 | Shoot-root ratio | 132.6 | 2 | 4.71 | 10.96 | S2_28317911 -S2_28375704 |
|  |  |  |  | qSRR2.31 | Shoot-root ratio | 146.39 | 2 | 3.2 | 7.62 | S2_31037977 -S2_31043939 |
|  |  |  |  | qSRR2.33 | Shoot-root ratio | 160.11 | 2 | 4.18 | 9.9 | S2_33573567 - S2_33614297 |
|  |  |  |  | qSRR2.34 | Shoot-root ratio | 170.41 | 2 | 2.94 | 7.37 | S2_34660774 -S2_35085922 |
|  |  |  |  | qSRR2.33 | Shoot-root ratio | 160.11 | 2 | 8.53 | 10.92 | S2_33573567 -S2_33614297 |
|  |  |  |  | qCHL3.26 | Chlorophyll content | 136.25 | 3 | 3.23 | 6.42 | S3_26705619 - S3_26709038 |
|  |  |  |  | qSHL3.34 | Shoot length | 183.74 | 3 | 2.36 | 5.65 | S3_34720589 - S3_35060080 |
|  |  |  |  | qSHL3.34 | Shoot length | 183.74 | 3 | 4.4 | 3.96 | S3_34720589 -S3_35060080 |
|  |  |  |  | qRTL3.6 | Root length | 35.76 | 3 | 3.47 | 8.23 | S3_6011601 - S3_6027452 |
|  |  |  |  | qRTL3.7 | Root length | 43.89 | 3 | 4.47 | 10.7 | S3_7130220 - S3_7209963 |
|  |  |  |  | qRTL3.10 | Root length | 57.41 | 3 | 5.04 | 11.99 | S3_10116591 - S3_10132745 |
|  |  |  |  | qRTL3.9 | Root length | 56.03 | 3 | 4.29 | 7.59 | S3_9853159 - S3_9891061 |
|  |  |  |  | qSRR3.8 | Shoot-root ratio | 49.29 | 3 | 2.65 | 6.32 | S3_8327882 -S3_8353264 |
|  |  |  |  | qSRR3.10 | Shoot-root ratio | 57.41 | 3 | 2.69 | 6.58 | S3_10116591- S3_10132745 |
|  |  |  |  | qSRR3.11 | Shoot-root ratio | 69.85 | 3 | 2.48 | 5.93 | S3_11848358 - S3_11865689 |
|  |  |  |  | qSRR3.9 | Shoot-root ratio | 56.03 | 3 | 4.33 | 5.25 | S3_9853159 - S3_9891061 |
|  |  |  |  | qRTL4.10 | Root length | 23.99 | 4 | 2.01 | 4.88 | S4_10625625- S4_10726368 |
|  |  |  |  | qDWT4.32 | Dry weight | 125.96 | 4 | 2.39 | 5.73 | S4_32367131 -S4_32367159 |
|  |  |  |  | qDWT4.32 | Dry weight | 125.96 | 4 | 3.66 | 5.93 | S4_32367131- S4_32367159 |
|  |  |  |  | qSRR4.10 | Shoot-root ratio | 23.99 | 4 | 2.44 | 5.91 | S4_10625625 -S4_10726368 |
|  |  |  |  | qK5.4 | K+ concentration | 32.8 | 5 | 2.25 | 5.51 | S5_4699921- S5_5326365 |
|  |  |  |  | qSIS5.03 | Salt injury score | 1.02 | 5 | 2.83 | 6.74 | S5_312457 -S5_329699 |
|  |  |  |  | qSIS5.1a | Salt injury score | 11.91 | 5 | 2.83 | 6.76 | S5_1686924 - S5_1707475 |
|  |  |  |  | qSIS5.24 | Salt injury score | 105.45 | 5 | 3.13 | 7.51 | S5_24057323 -S5_24281632 |
|  |  |  |  | qSIS5.1b | Salt injury score | 11.2 | 5 | 9.71 | 13.33 | S5_1441967 -S5_1454837 |
|  |  |  |  | qSHL5.4 | Shoot length | 29.74 | 5 | 2.32 | 5.64 | S5_4565557- S5_4699921 |
|  |  |  |  | qSHL5.6 | Shoot length | 43.96 | 5 | 2.03 | 4.96 | S5_6356744 -S5_6433933 |
|  |  |  |  | qSHL5.3 | Shoot length | 24.88 | 5 | 7.08 | 6.79 | S5_3353753 -S5_3506138 |
|  |  |  |  | qDWT5.2 | Dry weight | 15.26 | 5 | 4.63 | 10.8 | S5_2116055- S5_2167880 |
|  |  |  |  | qDWT5.4 | Dry weight | 29.74 | 5 | 6.58 | 15.04 | S5_4565557- S5_4699921 |
|  |  |  |  | qDWT5.5 | Dry weight | 41.58 | 5 | 6.54 | 15.47 | S5_5997340 -S5_6196044 |
|  |  |  |  | qDWT5.4 | Dry weight | 29.74 | 5 | 7.57 | 12.98 | S5_4565557 - S5_4699921 |
|  |  |  |  | qNa6.5 | Na+ concentration | 32.94 | 6 | 2.4 | 5.97 | S6_5269698 - S6_5533752 |
|  |  |  |  | qNa6.5 | Na+ concentration | 32.94 | 6 | 2.4 | 5.97 | S6_5269698- S6_5533752 |
|  |  |  |  | qK6.4 | K+ concentration | 28.94 | 6 | 3.33 | 8.21 | S6_4890290 -S6_5269698 |
|  |  |  |  | qNaK6.2 | Na/K ratio | 15 | 6 | 3.58 | 8.46 | S6_2927160 - S6_2962502 |
|  |  |  |  | qNaK6.5 | Na/K ratio | 32.94 | 6 | 5.12 | 13.21 | S6_5269698 - S6_5533752 |
|  |  |  |  | qNaK6.5 | Na/K ratio | 32.94 | 6 | 3.71 | 8.85 | S6_5269698 - S6_5533752 |
|  |  |  |  | qSIS6.2 | Salt injury score | 15 | 6 | 2.08 | 5.04 | S6_2927160 - S6_2962502 |
|  |  |  |  | qSIS6.5 | Salt injury score | 36.9 | 6 | 3.04 | 7.23 | S6_5848568 - S6_5905669 |
|  |  |  |  | qSIS6.7 | Salt injury score | 48.12 | 6 | 3.12 | 7.41 | S6_7646442 -S6_7661883 |
|  |  |  |  | qSIS6.20 | Salt injury score | 89.97 | 6 | 3.96 | 9.44 | S6_20929261 - S6_20929283 |
|  |  |  |  | qSIS6.2b | Salt injury score | 9.27 | 6 | 3.59 | 4.46 | S6_2123411- S6_2242943 |
|  |  |  |  | qSIS6.21 | Salt injury score | 92.17 | 6 | 6.92 | 9.11 | S6_21253244- S6_21256132 |
|  |  |  |  | qDWT6.13 | Dry weight | 71.07 | 6 | 2.01 | 5.16 | S6_13046472 - S6_13097774 |
|  |  |  |  | qDWT6.20 | Dry weight | 89.97 | 6 | 3.75 | 8.95 | S6_20929261- S6_20929283 |
|  |  |  |  | qDWT6.23 | Dry weight | 101.17 | 6 | 3.71 | 8.91 | S6_23812023 - S6_24039384 |
|  |  |  |  | qDWT6.06 | Dry weight | 2.73 | 6 | 3.71 | 6.02 | S6_692773 -S6_782975 |
|  |  |  |  | qDWT6.24 | Dry weight | 104.1 | 6 | 4.46 | 7.46 | S6_24107596- S6_24228831 |
|  |  |  |  | qSIS7.14 | Salt injury score | 57.13 | 7 | 3.62 | 4.5 | S7_14598897- S7_14625841 |
|  |  |  |  | qSIS8.24 | Salt injury score | 95.01 | 8 | 2.62 | 3.28 | S8_24763939 -S8_25110888 |
|  |  |  |  | qRTL8.4 | Root length | 37.12 | 8 | 2.12 | 5.34 | S8_4558562 -S8_4858127 |
|  |  |  |  | qRTL8.19 | Root length | 59.13 | 8 | 3.27 | 7.75 | S8_19884635 -S8_19898432 |
|  |  |  |  | qRTL8.27 | Root length | 108.84 | 8 | 2.1 | 5.13 | S8_27238050 -S8_27304101 |
|  |  |  |  | qSRR8.19 | Shoot root ratio | 59.13 | 8 | 2.38 | 5.7 | S8_19884635 -S8_19898432 |
|  |  |  |  | qSRR8.26 | Shoot root ratio | 107.22 | 8 | 2.53 | 3.01 | S8_26716230 -S8_26744324 |
|  |  |  |  | qSIS9.8 | Salt injury score | 12.51 | 9 | 7.09 | 9.19 | S9_8608506 -S9_9070610 |
|  |  |  |  | qRTL9.14 | Root length | 38.96 | 9 | 2.66 | 6.45 | S9_14960521 -S9_14976723 |
|  |  |  |  | qSIS11.2 | Salt injury score | 20.76 | 11 | 2.66 | 8.36 | S11_2838776 -S11_3716306 |
|  |  |  |  | qSIS11.2 | Salt injury score | 20.76 | 11 | 2.34 | 3.53 | S11_2838776 -S11_3716306 |
|  |  |  |  | qCHL11.1 | Chlorophyll content | 4.68 | 11 | 2.19 | 5.41 | S11_1086712 -S11_1293020 |
|  |  |  |  | qCHL11.2 | Chlorophyll content | 14.07 | 11 | 2.02 | 4.86 | S11_2666525 -S11_2724222 |
|  |  |  |  | qDWT11.2 | Dry weight | 9.97 | 11 | 2.44 | 6.03 | S11_2379158 -S11_2402109 |
|  |  |  |  | qNa12.18 | Na+ concentration | 60.06 | 12 | 2.25 | 5.51 | S12_18687038 -S12_18741493 |
|  |  |  |  | qNa12.18 | Na+ concentration | 60.06 | 12 | 2.25 | 5.51 | S12_18687038 -S12_18741493 |
|  |  |  |  | qSHL12.25 | Shoot length | 92.77 | 12 | 2.25 | 2.05 | S12_25709174 -S12_25887173 |
|  |  |  |  |  |  |  |  |  |  |  |
| Fayed and Farid (2017) | TCCP266/ Sakha102 | F2 | 288 | *qNa^+^LV-3* | Na+ concentration in leaf at vegetative stage | 173.3 | 3 | 2.19 | 6.3 | RM571-RM514 |
|  |  |  |  | *qNa^+^LR-3* | Na^+^ concentration in leaf at reproductive stage | 100.6 | 3 | 2.15 | 6.2 | RM282-RM156 |
|  |  |  |  | *qNa^+^SR-1* | Na+ concentration in stem at reproductive stage | 166.1 | 1 | 2.41 | 7 | RM472-RM104 |
|  |  |  |  | *qNa^+^SR-4* | Na+ concentration in stem at reproductive stage | 2.8 | 4 | 2.05 | 5.9 | RM307-RM335 |
|  |  |  |  | *qK^+^LV-3* | K+ concentration in leaf at vegetative stage | 221.1 | 3 | 2.17 | 6.3 | RM570-RM85 |
|  |  |  |  | *qK^+^SV-1* | K+ concentration in stem at vegetative stage | 77.8 | 1 | 3.52 | 5.3 | RM493-RM24 |
|  |  |  |  | *qK^+^SV-9* | K+ concentration in stem at vegetative stage | 70.7 | 9 | 3.09 | 7 | RM257-RM242 |
|  |  |  |  | *qNa^+^/K^+^ SV-9* | Na+/K+ ratio in stem at vegetative stage | 70.7 | 9 | 3.3 | 9.7 | RM257-RM242 |
|  |  |  |  | *qNa^+^/K^+^ LV-4* | Na+/K+ ratio in leaf at vegetative stage | 1.4 | 4 | 3.95 | 11.2 | RM307-RM335 |
|  |  |  |  | *qNa^+^/K^+^ LV-9* | Na+/K+ ratio in leaf at vegetative stage | 77 | 9 | 2.55 | 7.4 | RM242-RM201 |
|  |  |  |  |  |  |  |  |  |  |  |
| Wang et al. (2017) | 93-11/O. rufipogon accession | BC3ILs | 285 | qSTS1-1 | salt tolerance score | 22 | 1 | 3 | 4 | RM1 |
|  |  |  |  | qSR1-1 | Survival Rate | 22 | 1 | 3 | 5 | RM1 |
|  |  |  |  | qSR1-2 | Survival Rate | 141.7 | 1 | 2 | 2 | RM3825 |
|  |  |  |  | qSTS5-1 | salt tolerance score | 15 | 5 | 2 | 8 | RM3328 |
|  |  |  |  | qSTS5-2 | salt tolerance score | 53.6 | 5 | 2 | 8 | RM163 |
|  |  |  |  | qSR7 | Survival Rate | 38.7 | 7 | 2 | 4 | RM5436 |
|  |  |  |  | qSTS9 | salt tolerance score | 31.3 | 9 | 3 | 4 | RM296 |
|  |  |  |  | qSR9 | Survival Rate | 31.3 | 9 | 3 | 5 | RM296 |
|  |  |  |  | qSTS10 | salt tolerance score | 33.6 | 10 | 2 | 4 | RM258 |
|  |  |  |  | qSR10 | Survival Rate | 33.6 | 10 | 2 | 4 | RM258 |
|  |  |  |  | qSTS11-1 | salt tolerance score | 19.8 | 11 | 3 | 5 | RM124 |
|  |  |  |  | qSTS11-2 | salt tolerance score | 55 | 11 | 3 | 5 | RM7463 |
|  |  |  |  | qSR11-1 | Survival Rate | 19.8 | 11 | 2 | 5 | RM124 |
|  |  |  |  | qSR11-2 | Survival Rate | 55 | 11 | 2 | 4 | RM7463 |
|  |  |  |  | qSTS12 | salt tolerance score | 47.8 | 12 | 2 | 4 | RM7102 |
|  |  |  |  | qSR12 | Survival Rate | 47.8 | 12 | 2 | 4 | RM7102 |
|  |  |  |  |  |  |  |  |  |  |  |
| De Leon et al. (2017) | Bengal/ Pokkali | AB-Ils:BC4F4 | 292 | qSIS 1.39 | Salt injury score | **39.5** | 1 | 2.055 | 10.154 | RM3810 |
|  |  |  |  | qSIS2.3 | Salt injury score | **3** | 2 | 2.326 | 6.59 | RM211 |
|  |  |  |  | qSIS6.5 | Salt injury score | **5.5** | 6 | 2.498 | 7.04 | RM253 |
|  |  |  |  | qSIS 7.12 | Salt injury score | **12.8** | 7 | 2.081 | 5.942 | RM214 |
|  |  |  |  | qSIS 7.17 | Salt injury score | **17.5** | 7 | 2.935 | 16.989 | RM5793 |
|  |  |  |  | qSHL1.39 | Shoot length | **39.5** | 1 | 2.556 | 8.188 | RM3810 |
|  |  |  |  | qSHL1.41 | Shoot length | **41.1** | 1 | 2.091 | 6.795 | RM5362 |
|  |  |  |  | qSHL2.3 | Shoot length | **3** | 2 | 2.031 | 6.614 | RM211 |
|  |  |  |  | qSHL5.04 | Shoot length | **0.4** | 5 | 3.861 | 16.175 | RM17749 |
|  |  |  |  | qSHL6.5 | Shoot length | **5.4** | 6 | 5.34 | 23.491 | RM253 |
|  |  |  |  | qSHL7.12 | Shoot length | **12.8** | 7 | 2.067 | 6.722 | RM214 |
|  |  |  |  | qRTL2.20 | Root length | **20.7** | 2 | 2.203 | 12.976 | RM341 |
|  |  |  |  | qSRR2.37 | Shoot length to root length ratio | **37.6** | 2 | 3.882 | 18.174 | RM266 |
|  |  |  |  | qSRR7.12 | Shoot length to root length ratio | **12.8** | 7 | 2.857 | 12.936 | RM214 |
|  |  |  |  | qDWT2.3 | Shoot dry weight | **3** | 2 | 5.192 | 27.93 | RM211 |
|  |  |  |  | qDWT6.5 | Shoot dry weight | **5.4** | 6 | 2.921 | 8.966 | RM253 |
|  |  |  |  | qDWT7.12 | Shoot dry weight | **12.8** | 7 | 2.357 | 7.362 | RM214 |
|  |  |  |  | qDWT7.17 | Shoot dry weight | **17.5** | 7 | 2.324 | 7.266 | RM5793 |
|  |  |  |  | qNa11.5 | Shoot Na+ concentration | **5.61** | 11 | 2.04 | 9.68 | S11_5610372 |
|  |  |  |  | qK1.386 | Shoot K+ concentration | **38.63** | 1 | 2.23 | 10.66 | S1_38636497 |
|  |  |  |  | qNaK3.32 | Shoot Na+ /K+ Ratio | **32** | 3 | 2.31 | 11.27 | S3_32078967 |
|  |  |  |  | qSIS5.1 | Salt injury score | **1.47** | 5 | 2.36 | 5.53 | S5_1473882 |
|  |  |  |  | qSIS5.2 | Salt injury score | **2.83** | 5 | 2.07 | 4.97 | S5_2831482 |
|  |  |  |  | qSIS9.14 | Salt injury score | **14.6** | 9 | 2.18 | 5.25 | S9_14600108 |
|  |  |  |  | qSIS1.41 | Salt injury score | **41.81** | 1 | 2.05 | 6.17 | S1_41818521 |
|  |  |  |  | qSIS1.42 | Salt injury score | **42.31** | 1 | 2.27 | 6.8 | S1_42310908 |
|  |  |  |  | qSIS5.034 | Salt injury score | **0.34** | 5 | 2.5 | 8.14 | S5_340482 |
|  |  |  |  | qSIS9.8 | Salt injury score | **8.6** | 9 | 2.17 | 7.79 | S9_8608506 |
|  |  |  |  | qCHL3.6 | Chlorophyll content | **6.96** | 3 | 2.28 | 4.43 | S3_6962390 |
|  |  |  |  | qCHL3.25 | Chlorophyll content | **25.64** | 3 | 2.48 | 4.86 | S3_25640338 |
|  |  |  |  | qCHL3.26 | Chlorophyll content | **26.97** | 3 | 2.06 | 3.65 | S3_26978157 |
|  |  |  |  | qCHL11.2 | Chlorophyll content | **2.32** | 11 | 6.31 | 12.4 | S11_2322899 |
|  |  |  |  | qSHL1.3810 | Shoot length | **38.1** | 1 | 2.17 | 2.64 | S1_38108856 |
|  |  |  |  | qSHL1.3818 | Shoot length | **38.18** | 1 | 3.33 | 5.31 | S1_38181791 |
|  |  |  |  | qSHL1.3863 | Shoot length | **38.63** | 1 | 5.37 | 21.59 | S1_38636497 |
|  |  |  |  | qSHL1.3876 | Shoot length | **38.76** | 1 | 2.99 | 4.2 | S1_38768787 |
|  |  |  |  | qSHL1.40 | Shoot length | **40** | 1 | 2.81 | 3.89 | S1_40013502 |
|  |  |  |  | qSHL8.4 | Shoot length | **4.74** | 8 | 2.47 | 9.03 | S8_4747595 |
|  |  |  |  | qSRR1.3818 | Shoot length to root length ratio | **38.18** | 1 | 2.08 | 9 | S1_38181791 |
|  |  |  |  | qSRR1.3863 | Shoot length to root length ratio | **38.63** | 1 | 3.91 | 10.54 | S1_38636497 |
|  |  |  |  | qSRR8.5 | Shoot length to root length ratio | **5.34** | 1 | 3.16 | 8.27 | S8_5341936 |
|  |  |  |  | qSRR1.27 | Shoot length to root length ratio | **27.95** | 1 | 2.54 | 6.18 | S1_27956396 |
|  |  |  |  | qSRR1.2851 | Shoot length to root length ratio | **28.51** | 1 | 2.64 | 6.26 | S1_28513474 |
|  |  |  |  | qSRR1.2853 | Shoot length to root length ratio | **28.53** | 1 | 2.07 | 5.01 | S1_28535873 |
|  |  |  |  | qDWT1.41 | Shoot dry weight | **41.81** | 1 | 2.07 | 4.67 | S1_41818521 |
|  |  |  |  | qDWT1.42 | Shoot dry weight | **42.31** | 1 | 2.13 | 7.28 | S1_42310908 |
|  |  |  |  | qDWT7.17 | Shoot dry weight | **17.57** | 7 | 2.84 | 11.54 | S7_17569558 |
|  |  |  |  | qDWT7.18 | Shoot dry weight | **18.8** | 7 | 2.05 | 3.98 | S7_18801087 |
|  |  |  |  | qDWT7.20 | Shoot dry weight | **20.08** | 7 | 3.14 | 7.39 | S7_20085299 |
|  |  |  |  | qDWT5.034 | Shoot dry weight | **0.34** | 5 | 2.26 | 8.46 | S5_340482 |
|  |  |  |  |  |  |  |  |  |  |  |
| Rahman et al. (2017) | IR29/Hasawi | F5:6 RILs | 142 | qSES1.1 | Standard evaluation score | 110 | 1 | 3.2 | 10.7 | ud1000711-id1004348 |
|  |  |  |  | qSES1.2 | Standard evaluation score | 128 | 1 | 4.9 | 8.8 | id1004348-id1015258 |
|  |  |  |  | qSES1.3 | Standard evaluation score | 170 | 1 | 17.5 | 39.9 | id1024972-id1023892 |
|  |  |  |  | qSES1.4 | Standard evaluation score | 175 | 1 | 20.6 | 42.3 | id1023892-id1017885 |
|  |  |  |  | qSES1.5 | Standard evaluation score | 194 | 1 | 3.8 | 5.4 | id1003559-id1002308 |
|  |  |  |  | qSL1.2 | Shoot length | 169 | 1 | 6.5 | 17.9 | id1024972-id1023892 |
|  |  |  |  | qSL1.3 | Shoot length | 177 | 1 | 5.7 | 19.5 | id1023892-id1017885 |
|  |  |  |  | qSL1.4 | Shoot length | 221 | 1 | 5.6 | 15.3 | id1024836-id1016633 |
|  |  |  |  | qRL1.1 | Root length | 168 | 1 | 4.8 | 14.3 | id1024972- id1023892 |
|  |  |  |  | qRL1.2 | Root length | 175 | 1 | 4.2 | 13.5 | id1023892-id1017885 |
|  |  |  |  | qFWsht1.1 | Shoot fresh weight | 0 | 1 | 5.5 | 10.2 | id1002899-id1016436 |
|  |  |  |  | qFWsht1.2 | Shoot fresh weight | 175 | 1 | 6.3 | 21.1 | id1023892-id1017885 |
|  |  |  |  | qFWsht1.3 | Shoot fresh weight | 194 | 1 | 4.5 | 13.8 | id1003559-id1002308 |
|  |  |  |  | qDWsht1.1 | Shoot dry weight | 0 | 1 | 3.4 | 5.5 | id1002899-id1016436 |
|  |  |  |  | qDWsht1.2 | Shoot dry weight | 175 | 1 | 3.4 | 19.6 | id1023892-id1017885 |
|  |  |  |  | qDWsht1.3 | Shoot dry weight | 194 | 1 | 3.9 | 12 | id1003559-id1002308 |
|  |  |  |  | qRL3.1 | Root length | 104 | 3 | 3.2 | 21.9 | id3200001-id3010345 |
|  |  |  |  | qSES4.1 | Standard evaluation score | 32 | 4 | 3.8 | 5.8 | id4008522-id4008092 |
|  |  |  |  | qFWsht4.1 | Shoot fresh weight | 12 | 4 | 6.1 | 28.9 | id4003259-id4007105 |
|  |  |  |  | qFWsht4.2 | Shoot fresh weight | 32 | 4 | 4.3 | 8.8 | id4008522-id4008092 |
|  |  |  |  | qDWsht4.1 | Shoot dry weight | 13 | 4 | 8.3 | 30.1 | id4003259-id4007105 |
|  |  |  |  | qDWsht4.2 | Shoot dry weight | 32 | 4 | 3.2 | 5.9 | id4008522-id4008092 |
|  |  |  |  | qDWsht7.1 | Shoot dry weight | 107 | 7 | 3.3 | 5.7 | ud7000066-id7000461 |
|  |  |  |  | qRL11.1 | Root length | 22 | 11 | 5 | 14.7 | id11007488-id11008862 |
|  |  |  |  | qSL12.1 | Shoot length | 58 | 12 | 3.3 | 7.2 | id12007988-id12005823 |
|  |  |  |  | qFWsht12.1 | Shoot fresh weight | 78 | 12 | 4.4 | 8 | id12003019-id12005205 |
|  |  |  |  | qSL1.1 | Shoot length | 59 | 1 | 4.4 | 18.5 | id1024836-id1025983 |
|  |  |  |  | qFWsht2.1 | Shoot fresh weight | 55 | 2 | 3.1 | 27.2 | id2007526-fd12 |
|  |  |  |  | qDWsht5.1 | Shoot dry weight | 38 | 5 | 3.2 | 46.9 | id5007714-id5014589 |
|  |  |  |  | qFWsht6.1 | Shoot fresh weight | 115 | 6 | 3.2 | 37.8 | id6016941-id6001397 |
|  |  |  |  | qFWsht6.1 | Shoot fresh weight | 115 | 6 | 3.3 | 47.1 | id6016941-id6001397 |
|  |  |  |  | qDWsht6.1 | Shoot dry weight | 115 | 6 | 3.7 | 48.4 | id6016941-id6001397 |
|  |  |  |  | qFWsht8.1 | Shoot fresh weight | 105 | 8 | 3.5 | 47.1 | id8007301-id8000240 |
|  |  |  |  | qDWsht8.1 | Shoot dry weight | 105 | 8 | 3.8 | 47.2 | id8007301-id8000240 |
|  |  |  |  |  |  |  |  |  |  |  |
| Bizimana et al. (2017) | IR29/Hasawi | F5 RILs | 142 | qSL1.1 | Shoot length | 162.6 | 1 | 7.21 | 20.6 | id1023892–id1024836 |
|  |  |  |  | qSL1.2 | Shoot length | 168.6 | 1 | 3.93 | 11.8 | id1024972–id1025983 |
|  |  |  |  | qSESI2.1 | Initial SES | 60.8 | 2 | 3.5 | 10.6 | id2004774–id2007526 |
|  |  |  |  | qSESF2.1 | Final SES | 64.8 | 2 | 3.67 | 11.1 | id2004774–id2007526 |
|  |  |  |  | qSDW2.1 | Shoot dry weight | 62.8 | 2 | 3.84 | 11.6 | id2004774–id2007526 |
|  |  |  |  | qSFW4.1 | Shoot fresh weight | 18.1 | 4 | 4.28 | 12.8 | id4001113–id4001932 |
|  |  |  |  | qRFW4.1 | Root fresh weight | 98.1 | 4 | 3.38 | 10.3 | id4007444–id4008092 |
|  |  |  |  | qRDW4.1 | Root dry weight | 18.1 | 4 | 3.29 | 10 | id4001113–id4001932 |
|  |  |  |  | qSESI6.1 | Initial SES | 52.5 | 6 | 3.98 | 12 | ud6000572–id6009055 |
|  |  |  |  | qRL6.1 | Root length | 18.5 | 6 | 4.04 | 12.1 | fd13–id6004343 |
|  |  |  |  | qSL6.1 | Shoot length | 18.5 | 6 | 4.04 | 12.1 | fd13–id6004343 |
|  |  |  |  | qSDW8.1 | Shoot dry weight | 98.9 | 8 | 3.07 | 9.4 | id8006485–wd8004122 |
|  |  |  |  | qSFW9.1 | Shoot fresh weight | 21 | 9 | 4.2 | 12.6 | id9001614–id9002014 |
|  |  |  |  | qRFW9.1 | Root fresh weight | 27 | 9 | 3.34 | 10.1 | id9001614–id9002014 |
|  |  |  |  | qSESI12.1 | Initial SES | 84.9 | 12 | 3.54 | 10.7 | id12005823–d12007988 |
|  |  |  |  | qSESF12.1 | Final SES | 6.9 | 12 | 3.52 | 10.6 | id12000252–id12001321 |
|  |  |  |  | qRL12.1 | Root length | 6.9 | 12 | 3.81 | 11.5 | id12000252–id12001321 |
|  |  |  |  | qSL12.1 | Shoot length | 6.9 | 12 | 3.24 | 9.8 | id12000252–id12001321 |
|  |  |  |  | qSFW12.1 | Shoot fresh weight | 6.9 | 12 | 3.11 | 9.5 | id12000252–id12001321 |
|  |  |  |  | qSDW12.1 | Shoot dry weight | 6.9 | 12 | 3.06 | 9.3 | id12000252–id12001321 |
|  |  |  |  |  |  |  |  |  |  |  |
| Puram et al. (2017) | Jupiter/Nona Bokra | BC3F4 ILs | 138 | qSIS1.1 | salt injury score | 96.9 | 1 | 2.26 | 4.1 | RM9–RM5 |
|  |  |  |  | qK1.2 | Shoot K+ concentration | 47.8 | 1 | 3.51 | 5.7 | RM3412–RM10748 |
|  |  |  |  | qNaK1.1 | shoot Na-K concentration Ratio | 96.9 | 1 | 2.08 | 8.3 | RM9–RM5 |
|  |  |  |  | qCHL1.1 | chlorophyll content | 44.1 | 1 | 2.08 | 2.3 | RM23–RM1287 |
|  |  |  |  | qCHL1.2 | chlorophyll content | 47.8 | 1 | 2.93 | 0.7 | RM3412–RM10748 |
|  |  |  |  | qCHL1.3 | chlorophyll content | 51.4 | 1 | 3.28 | 3.6 | RM10748–RM10825 |
|  |  |  |  | qSIS2.1 | salt injury score | 32.3 | 2 | 2.13 | 7.5 | RM5780–RM29 |
|  |  |  |  | qSIS2.2 | salt injury score | 68.9 | 2 | 2.43 | 4.5 | RM6374–RM3284 |
|  |  |  |  | qNa2.1 | Shoot Na+ concentration | 80.4 | 2 | 2.07 | 6.8 | RM3284–RM262 |
|  |  |  |  | qNa2.2 | Shoot Na+ concentration | 121.1 | 2 | 2.37 | 2.5 | RM221–RM240 |
|  |  |  |  | qK2.1 | Shoot K+ concentration | 32.3 | 2 | 2.16 | 10.1 | RM5780–RM29 |
|  |  |  |  | qK2.2 | Shoot K+ concentration | 52.2 | 2 | 2.21 | 5.2 | RM29–RM6374 |
|  |  |  |  | qNa3.1 | Shoot Na+ concentration | 136.3 | 3 | 3.18 | 5.9 | RM3525–RM7389 |
|  |  |  |  | qCHL3.1 | chlorophyll content | 136.3 | 3 | 3.72 | 3.6 | RM3525–RM7389 |
|  |  |  |  | qRTL3.1 | Root length | 18.7 | 3 | 2.24 | 7 | RM5474–RM5480 |
|  |  |  |  | qK4.1 | Shoot K+ concentration | 105 | 4 | 2.75 | 8.9 | RM3839–RM3288 |
|  |  |  |  | qNaK4.1 | shoot Na-K concentration Ratio | 105 | 4 | 3.12 | 10.1 | RM3839–RM3288 |
|  |  |  |  | qSHL4.1 | Shoot length | 135.1 | 4 | 2.77 | 6.5 | RM348–RM5506 |
|  |  |  |  | qDWT4.1 | Shoot dry weight | 87.9 | 4 | 2.01 | 4.7 | RM3742–RM3866 |
|  |  |  |  | qSIS5.1 | salt injury score | 89.5 | 5 | 2.52 | 7.3 | RM161–RM6054 |
|  |  |  |  | qCHL6.1 | chlorophyll content | 28.4 | 6 | 3.87 | 3.7 | RM111–RM136 |
|  |  |  |  | qRTL6.1 | Root length | 17.4 | 6 | 2.85 | 9 | RM225–RM111 |
|  |  |  |  | qDWT7.1 | Shoot dry weight | 10.4 | 7 | 3.03 | 6 | RM295–RM6574 |
|  |  |  |  | qNa8.1 | Shoot Na+ concentration | 10 | 8 | 2.89 | 6.3 | RM408–RM1111 |
|  |  |  |  | qCHL8.1 | chlorophyll content | 10 | 8 | 4.79 | 3.7 | RM408–RM1111 |
|  |  |  |  | qCHL8.2 | chlorophyll content | 46.7 | 8 | 4.14 | 3.8 | RM72–RM331 |
|  |  |  |  | qCHL10.1 | chlorophyll content | 77.4 | 10 | 2.22 | 3.3 | RM5274–RM7300 |
|  |  |  |  | qSHL11.1 | Shoot length | 82.8 | 11 | 3.77 | 12.5 | RM229–RM206 |
|  |  |  |  | qRTL11.1 | Root length | 82.8 | 11 | 3.98 | 9.7 | RM229–RM206 |
|  |  |  |  | qCHL12.1 | chlorophyll content | 13.2 | 12 | 7.53 | 5.7 | RM3483–RM7619 |
|  |  |  |  | qCHL12.2 | chlorophyll content | 34.3 | 12 | 13.66 | 6.2 | RM7619–RM1337 |
|  |  |  |  | qCHL12.3 | chlorophyll content | 54.1 | 12 | 2.82 | 3.4 | RM1337–RM179 |
|  |  |  |  | qCHL12.4 | chlorophyll content | 73.6 | 12 | 3.98 | 3.7 | RM179–RM309 |
|  |  |  |  |  |  |  |  |  |  |  |
| Puram et al. (2018) | Cheniere/ Nona Bokra | BC3F4 ILs | 112 | qK1.1 | Shoot K+ concentration | 80.1 | 1 | 2.2 | 12.4 | RM7075-RM5 |
|  |  |  |  | qNaK1.1 | shoot Na/K Ratio | 80.1 | 1 | 2.4 | 11.5 | RM7075-RM5 |
|  |  |  |  | qSHL1.1 | Shoot length | 14.8 | 1 | 3.6 | 4.6 | RM3740-RM220 |
|  |  |  |  | qSRI-Na1.1 | relative shoot sodium concentration compared to control | 53.32 | 1 | 2.4 | 8.8 | RM10748-RM10864 |
|  |  |  |  | qSRI-RTL1.1 | relative root length compared to control | 14.8 | 1 | 3.1 | 3.4 | RM3740-RM220 |
|  |  |  |  | qSIS2.1 | salt injury score | 112.38 | 2 | 2.2 | 4.4 | RM106-RM6 |
|  |  |  |  | qSRI-K2.1 | relative shoot potassium concentration compared to control | 46.66 | 2 | 2.2 | 2.6 | RM29-RM550 |
|  |  |  |  | qSRI-NaK2.1 | relative shoot sodium concentration compared to control | 46.66 | 2 | 2.4 | 2.4 | RM29-RM550 |
|  |  |  |  | qSRI-SHL2.1 | relative shoot length compared to control | 133.98 | 2 | 2.5 | 1.2 | RM6-RM138 |
|  |  |  |  | qSIS3.1 | salt injury score | 18.7 | 3 | 5.8 | 7.3 | RM5474-RM5480 |
|  |  |  |  | qK3.1 | Shoot K+ concentration | 61.7 | 3 | 5.5 | 14.8 | RM282-RM156 |
|  |  |  |  | qNaK3.1 | shoot Na/K Ratio | 61.7 | 3 | 4.8 | 14.6 | RM282-RM156 |
|  |  |  |  | qRTL3.1 | root length | 18.7 | 3 | 2.3 | 2.5 | RM5474-RM5480 |
|  |  |  |  | qRTL3.2 | root length | 116.9 | 3 | 5.4 | 2.9 | RM3350-RM3525 |
|  |  |  |  | qSRI-RTL3.1 | relative root length compared to control | 116.9 | 3 | 2.4 | 8.3 | RM3350-RM3525 |
|  |  |  |  | qSHL4.1 | Shoot length | 10.5 | 4 | 5.2 | 10.9 | RM335-RM 8213 |
|  |  |  |  | qSHL4.2 | Shoot length | 103.5 | 4 | 6.2 | 13.6 | RM3866-RM3288 |
|  |  |  |  | qSIS5.1 | salt injury score | 28.1 | 5 | 2.2 | 3.4 | RM1366-RM249 |
|  |  |  |  | qSIS5.2 | salt injury score | 73.6 | 5 | 2.2 | 3.8 | RM6645-RM161 |
|  |  |  |  | qRTL5.1 | root length | 7 | 5 | 3.3 | 2.7 | RM159-RM1366 |
|  |  |  |  | qRTL5.2 | root length | 73.6 | 5 | 3.5 | 2.7 | RM6645-RM161 |
|  |  |  |  | qSRI-RTL5.1 | relative root length compared to control | 7 | 5 | 2.3 | 7.8 | RM159-RM1366 |
|  |  |  |  | qCHL6.1 | chlorophyll content | 58.5 | 6 | 2.2 | 8.9 | RM8250-RM2229 |
|  |  |  |  | qRTL6.1 | root length | 88.6 | 6 | 3.2 | 2.9 | RM3-RM6298 |
|  |  |  |  | qNa7.1 | Shoot Na+ concentration | 84.5 | 7 | 2.1 | 6 | RM11-RM6810 |
|  |  |  |  | qRTL7.1 | root length | 84.5 | 7 | 2.8 | 2.5 | RM11-RM6810 |
|  |  |  |  | qRTL7.2 | root length | 104.6 | 7 | 2.5 | 2.7 | RM6432-RM3555 |
|  |  |  |  | qSRI-K7.1 | relative shoot potassium concentration compared to control | 72.3 | 7 | 2.3 | 2.6 | RM2-RM11 |
|  |  |  |  | qSRI-K7.2 | relative shoot potassium concentration compared to control | 104.6 | 7 | 2.1 | 2.5 | RM6432-RM3555 |
|  |  |  |  | qSRI-NaK7.1 | relative shoot sodium to potassium ratio compared to control | 72.3 | 7 | 2.6 | 2.9 | RM2-RM11 |
|  |  |  |  | qSIS8.1 | salt injury score | 91.3 | 8 | 7.1 | 9.2 | RM515-RM256 |
|  |  |  |  | qK8.1 | Shoot K+ concentration | 75.5 | 8 | 4 | 10 | RM44-RM515 |
|  |  |  |  | qNaK8.1 | shoot Na/K Ratio | 75.5 | 8 | 3.5 | 10.5 | RM282-RM156 |
|  |  |  |  | qSHL8.1 | Shoot length | 62.6 | 8 | 9.5 | 14.2 | RM137-RM44 |
|  |  |  |  | qRTL8.1 | root length | 62.6 | 8 | 9.9 | 4.6 | RM137-RM44 |
|  |  |  |  | qDWT8.1 | shoot dry weight | 75.5 | 8 | 4.9 | 17.6 | RM44-RM515 |
|  |  |  |  | qSRI-RTL8.1 | relative root length compared to control | 62.6 | 8 | 2.1 | 2.3 | RM137-RM44 |
|  |  |  |  | qSRI-DWT8.1 | relative dry weight compared to control | 106.8 | 8 | 3.2 | 2.5 | RM258-RM147 |
|  |  |  |  | qRTL9.1 | root length | 47.8 | 9 | 3.5 | 2.8 | RM296-RM105 |
|  |  |  |  | qDWT9.1 | shoot dry weight | 67.9 | 9 | 2.2 | 7.8 | RM3700-RM257 |
|  |  |  |  | qSRI-K9.1 | relative shoot potassium concentration compared to control | 47.8 | 9 | 4.6 | 12.5 | RM296-RM105 |
|  |  |  |  | qSRI-NaK9.1 | relative shoot sodium to potassium ratio compared to control | 47.8 | 9 | 4.8 | 13.3 | RM296-RM105 |
|  |  |  |  | qRTL10.1 | root length | 43.6 | 10 | 3 | 2.9 | RM294A-RM6124 |
|  |  |  |  | qRTL10.2 | root length | 88.9 | 10 | 2.1 | 2.2 | RM147-RM228 |
|  |  |  |  | qSRI-RTL10.1 | relative root length compared to control | 26.8 | 10 | 2.1 | 2.2 | RM216-RM294A |
|  |  |  |  | qSRI-DWT10.1 | relative dry weight compared to control | 79.8 | 10 | 5.1 | 2.1 | RM258-RM147 |
|  |  |  |  | qRTL11.1 | root length | 88.8 | 11 | 2.5 | 2.9 | RM21-RM187 |
|  |  |  |  | qDWT11.1 | shoot dry weight | 30.2 | 11 | 2 | 6.9 | RM116-RM202 |
|  |  |  |  | qSRI-SHL12.1 | relative shoot length compared to control | 51.3 | 12 | 2.1 | 2.2 | RM270-RM179 |
|  |  |  |  | qSRI-DWT12.1 | relative dry weight compared to control | 93.8 | 12 | 2.9 | 2.3 | RM309-RM6052 |
|  |  |  |  |  |  |  |  |  |  |  |
| Dahanayaka et al. (2017) | At354 x Bg352 | F5 RILs | 100 | qSSI1 | Salinity survival index | 50 | 1 | 3.3 | 10.8 | RM140-RM10852 |
|  |  |  |  | qSL1 | Shoot length | 50 | 1 | 2.7 | 10 | RM140-RM10852 |
|  |  |  |  | qSNK1 | shoot Na+/K+ ratio | 48 | 1 | 2.1 | 8.9 | RM10745-RM140 |
|  |  |  |  | qSSI4 | Salinity survival index | 134 | 4 | 3.6 | 15 | RM3843-RM280 |
|  |  |  |  | qSL4 | Shoot length | 134 | 4 | 2.7 | 11 | RM3843-RM280 |
|  |  |  |  | qSNK4 | shoot Na+/K+ ratio | 30 | 4 | 4.5 | 16 | RM518-RM5749 |

* Bold face numbers indicate the QTL position in Mb while non-bold numerals in cM
