# Supplementary material for: Salt tolerance in rice: seedling and reproductive stage QTL mapping come of age
Source: Theor Appl Genet. 2021 Jul 21;134(11):3495–533. doi: 10.1007/s00122-021-03890-3 (PMC8519845; doi:10.1007/s00122-021-03890-3)
Supplement: Supplementary file 2 — Supplementary file2 (DOCX 24 kb) [file 122_2021_3890_MOESM2_ESM.docx]

**Online Resource 2: Initial QTLs mapped with Meta-QTLs and their associated traits**

| **Meta QTL** | **Chrom-osome** | **Initial QTLs mapped to Meta-QTLs** | **Traits assocaited with Meta-QTLs** |
| --- | --- | --- | --- |
| mQTL 1.1 | 1 | qSRR1.29, qDWT1.21, qNaK1.11, qSRR1.36, qCHL1.1, qDWT1.40, qK1.2, qRTL1.22, qCHL1.2, qK1.11, qSHL1.7a, qSIS-1.1, qSTS1-1, qSHL1.7b, qSRR1.7, qSRI-RTL1.1, qSR1-1, qSHL1.1, qClSV-1.1, qNa/KSV-1.1, qKSV-1.1, qNaSV-1.1, qKC1.1, qDWsht1.1, qFWsht1.1 | SRR, DWT, NaK, CHL, DWT, K, RTL, SHL, SIS, STS, SHL, SRI-RTL, SR, ClSV, Na/KSV, KSV, NaSV, KC, DWsht, FWsht |
| mQTL 1.2 | 1 | qRKC1, qKr1.1, qKC1.2, qNas/Ks1, qNDS-1.0, qSNC-1, qSNC1, qFWsht1.2, qRNK1, qSL1.4, qSL1.3, qSKC-1, SalTol1-1, qRL1.2, qSRI-Na1.1, qSKC1, qSNK1, qDWsht1.2, qSIS-1.2, qKC-1, qSES1.4, qK1.8, qRL-1 | RKC, Kr, KC, Nas/Ks, NDS, SNC, FWsht, RNK, SL, SKC, SalTol, RL, SRI-Na, SNK, DWsht, SIS, SES, |
| mQTL 1.3 | 1 | qK1.1, qNaK1.1, qNU-1, qCHL1.3, qNKR-1, qSSI1, qSNK_1, qSL1, qCFW-1, qKC1.3 | K, NaK, NU, CHL, NKR, SSI, SNK, SL, CFW, KC |
| mQTL 1.4 | 1 | qSTR-1, qCDW-1, qSDW1.1, qKC1.4, qKr1.2, qNar/Kr1, qSH1.1 | STR, CDW, SDW, KC, Kr, Nar/Kr, SH |
| mQTL 1.5 | 1 | qPF1.4, qSIS1.1, qSST1, qRTL1.26, qGR-1, qNas1, qSKC1.1, qNa1.7, qNaKR1.8 | PF, SIS, SST, RTL, GR, Nas, SKC, Na, NaKR |
| mQTL 1.6 | 1 | qPL1.1s, qPH1.1s, qSNaKR-1, qSUR1, qCHL_1, qPL1.2, qPF1.5, qPH1.1, qSDS-1, qRNTQ-1, qSL-1.1, qSR1-2, qSL-1.3, qSL-1.2, qSL-1.4, qNa1.6, qSKC1.2 | PL, PH, SNaKR, SUR, CHL, PL, PF, PH, SDS, RNTQ, SL, SR,Na6, SKC |
| mQTL 1.7 | 1 | qSRR1.386, qSRR1.382, qRDW-1, saltol-qNU-KU-NKR-1, qK1.38 | SRR, SRR, RDW, saltol-qNU-KU-NKR, K |
| mQTL 2.1 | 2 | qRTL2.33, qPDS2 , qSKC2, qRTL2.24, qSHL2.18, qRDW2, qSNK_2, qCHL2.30, qSDW2, qRTL2.26, qSSI2, qSRR2.28, qSIS2.19, qSRR2.34, qSRR2.31, qSRR2.33, qCHL2.20, qSNC2, qSL2, qSIS2.28, qSFW2, qRFW2, qNaSV-2.1, qTN-2.1, qTN-2.2, qK2.1, qNa/kSV-2.2, qK2.2, qNDS-2.1, qSES-2, qClLR-2.1, qSIS2.8, FRSP2.1s, qClSV-2.1, qSNC-2, qSPFR2.1s, qSST2, qSDW2.1, qSRI-NaK2.1, qSKC-2, qDSS2.1, qSDW-2, qTN-2.3, qSRI-K2.1, qGY2.1s, qNaLR-2.1, qNa/kSV-2.1, qSIS2.2, qPL2.1s, qKC2.1, qGY2.1, qSUR2, qNa2.7, qTN2.1, qNa/kSV-2.3, qTN-2.4, qGP-2, qCHL_2, qPH2, qRKC2, qNDS-2.2, qSNaKR-2, qCHL2, | CHL, ClLR, ClSV, DSS, FRSP, GP, GY, K, KC, Na, Na/kSV, NaLR, NaSV, NDS, PDS, PH, PL, RDW, RFW, RKC, RTL, SDW, SES, SFW, SHL, SIS,SKC, SL, SNaKR, SNC, SNK, SPFR, SRI-NaK, SRR, SSI, SST,SUR, TN,SRI-K, |
| mQTL 2.2 | 2 | qNaC2.1, qKC2.2, qPF2.1, qNa2.1, qNa2.2, qNAK-2, qDW2.2, qSRI-SHL2.1, qSIS2.1 | DW, KC, Na, NaC NAK,PF, SIS,SRI-SHL |
| mQTL 3.1 | 3 | qSHL3.34, qSRR3.11, qRTL3.6, qSFW-3, qBM-3, qSIS3.1, qRTL3.1, qKC3.1, qSDW-3, qDSS3, qKC3.2, qSES3.1, qGY3.1 | BM, DS, GY, KC, RTL, SDW, SES, SFW, SHL, SIS, SRR |
| mQTL 3.2 | 3 | qRTL3.7, qLB-3 | RTL, LB |
| mQTL 3.3 | 3 | qSRR3.8 | SRR |
| mQTL 3.4 | 3 | qKr3, qSTSP3.1s qRTL3.10, qSRR3.10, qSL-3.2, qSL-3.1, qSRR3.9, qRTL3.9 | Kr, RTL, SL, SRR, STSP |
| mQTL 3.5 | 3 | qSKC3, qK3.1, qNaK3.1, qSNC3, qNaLV-3.1, qClLV-3.1, qPFW-3, qSL-3.3, qSL-3.4, qSIS-3, qRW-3.3, qNa/kLR-3.1, qGR-3-a, qNaLR-3.1, qSFW3.1, qKLV_3.1, qNa/kSV-3.1 | ClLV, GR, K, KLV, Na/kLR, Na/kSVNaK, NaLR, NaLV, PFW, RW, SFW, SIS, SKC, SL, SNC, |
| mQTL 3.6 | 3 | qSES3.2, qPH3.1s, qCHL3.26, qCHL3.1, qRDW-3, qSDW3, qSES3, qSL3, qRTL3.2, qCHL3, qRW-3.2, qCHL_3, qGR-3-b, qSFW3.2, qSRI-RTL3.1, qSNK_3, qPL3.1s, qNa3.1 | CHL, GR, Na, PH, PL, RDW, RTL, RW, SDW, SES, SFW, SL, SNK, SRI-RTL, |
| mQTL 4.1 | 4 | qDWT4.32, qSFW4, qRFW4, qRDW4.1, qSNC4, qKC4.1, qGY4.1s, qFRSP4.1s, qSTW4.1s, qTSP4.1s, qSHL4.1, qNaC4.1 | DWT, FRSP, GY, KC, NaC,RDW, RFW, SFW, SHL, SNC, STW, TSP |
| mQTL 4.10 | 4 | qSL4, qSSI4, qCHL4, qSES4, qSIS-4.2 | CHL, SES, SIS, SL, SSI, |
| mQTL 4.2 | 4 | qGP-4, qRFW-4a | GP, RFW |
| mQTL 4.3 | 4 | qRFW-4b, qPN4.1s, qRTL4.10, qSRR4.10, qPF4.1 | PF, PN, RFW, RTL, SRR |
| mQTL 4.4 | 4 | qRFW-4b, qPN4.1s, qRTL4.10, qSRR4.10, qPF4.1 | PF, PN, RFW, RTL, SRR |
| mQTL 4.5 | 4 | qSTR-4, qSFW4.1 | ST, SFW |
| mQTL 4.6 | 4 | qKs4, qFWsht4.1, qKC4.2, qRFW4.1, qSES4.1, qDWsht4.1, qDWsht4.2, qDWT4.1, qFWsht4.2, qDTF4.1s | DTF, DWsht, FWsht, KC, Ks, RFW, SES |
| mQTL 4.7 | 4 | qSHL4.2, qRDW4.2, qK4.1,qNaK4.1, qSSI4.1, qSFW-4a | K, NaK, RDW, SFW,SHL, SSI, |
| mQTL 4.8 | 4 | qPH4.2 | PH |
| mQTL 4.9 | 4 | qKr4, qNar/Kr4, qSIS-4.1, qSSI4.2, qKC4.3,qRDW-4, qSDW4, qSNK_4.2, qSFW-4b, qPH4, qPH4.1 | KC, Kr, Nar/Kr, PH, RDW, SDW, SFW, SIS, SNK, SSI, |
| mQTL 5.1 | 5 | qSIS5.24, qSHL5.3, qSIS5.03, qSIS5.1a, qSIS5.1b | qSHL, qSIS, |
| mQTL 5.2 | 5 | qSNC5, qSRI-RTL5.1, qRTL5.1,qPL5.1, qDWT5.2, qTGW5.1s, qNar/Kr5 | DWT, Nar/Kr, PL, RTL, SNC, SRI-RTL, TGW, |
| mQTL 5.3 | 5 | qSNK-5, qK5.4, qSHL5.4, qDWT5.4, qKs5, qSIS5.1, qSTS5-1 | DWT, K, SHL, SIS, SNK, STS |
| mQTL 5.4 | 5 | qSTR-5, qSHL-5, qSHL5.6, qDWT5.5 | qDWT, qSHL, qSTR, |
| mQTL 5.5 | 5 | qPDS5, qSST5, qBM-5b, qDWsht5.1, qBM-5a, qSDW-5b, qSDW-5a,qSDW5, qSES5, qSIS5.2, qRDW5, qSFW-5a, qSFW-5b, qSSI5, qSTS5-2,qSFW5, qSPFR5.1s, qRTL5.2,qPH5.1s, qCL-5, qSKC-5 | BM, CL, DWsht, PDS, PH, RDW, RTL, SDW, SES, SFW, SIS, SKC,SPFR, SSI, SST |
| mQTL 6.1 | 6 | qNaK6.5, qSIS6.5, qDWT6.24, qKC6.1, qSIS6.20, qNaK6.2, qNa6.5, qSIS6.2b, qRDW-6, qSIS6.7, qDWT6.06, qSIS6.21, qRL6, qSIS6.2, qDWT6.20, qDWT6.23, qSHL-6, qDTF6.1s, qK6.4, qCHL6.1, qSSI6, qDRW6, qIR-6, qRFW-6, qPDS6, qPF6.1, | CHL, DRW, DTF, DWT, IR, K, KC, Na, NaK, PDS, PF,RDW, RFW, RL, SHL, SIS, SSI, |
| mQTL 6.2 | 6 | qSES6, qNas6, qTGW6.1s, qFRSP6.1s, qPN6.1s, qGY6.1s, qRTL6.1, qBM-6, qDWT6.13, qNAK-6, qDSW6.1, qDSW6.2, qRNC-6,qRKC-6, qSFW-6, qKC6.2 | BM, DSW, DWT, FRSP, GY, KC, NAK, Nas, PN, RKC, RTL, SES, SFW, TGW, |
| mQTL 7.1 | 7 | qDWsht7.1, qPH7.2, qRFW7, qSDW7,qDWT7.1, qRDW7,qTN7.2, qSES-7,qSFW7, qPDW-7, qPH7.1 | DWsht, DWT, PDW, PH, RDW, SDW, SES,SFW, TN, |
| mQTL 7.2 | 7 | qSR7, qTN7.3, qPL7.4, qSIS7.14, qKC7.1, qNaC7.1, qSRI-K7.1, qSRI-NaK7.1 | KC, NaC, PL, SIS, SR, SRI-K, SRI-NaK, TN, |
| mQTL 7.3 | 7 | qSTW7.1s, qPH7.1s, qTSP7.1s, qNa7.1, qRNC-7, qSRI-K7.2, qSTSP7.1s, qRTL7.2, qRTL7.1, qSST7 | Na, PH, RNC, RTL, SRI-K, SST,STSP, STW, TSP, |
| mQTL 8.1 | 8 | qRTL8.4, qCHL8.1, qNa8.1, qCHL-8, qTN8.1, qSTW8.1s | CHL, Na, RTL, STW, TN, |
| mQTL 8.2 | 8 | SalTol8-1, qSNK-8, qNaLV-8.1, qDWS-8, qSDW8.1 | DWS, NaLV, SDW, SNK, SalTol, |
| mQTL 8.3 | 8 | qCFW-8, qCHL8.2, qSRR8.19, qRTL8.19, qBM8.2, qSRI-RTL8.1, qSHL8.1, qRTL8.1, qNa/kSV-8.1, qNaLV-8.2, qNa/kLR-8.1, qNaSV-8.1, qKLR-8.1, qNaLR-8.1 | BM, CFW, CHL, KLR, Na/kLR, Na/kSV, NaLR, NaLV, NaSV, RTL, SHL, SRI-RTL, SRR, |
| mQTL 8.4 | 8 | qSTR-8, qNaK8.1, qDWT8.1, qK8.1, qPL-8 | DWT, K, NaK, PL, STR |
| mQTL 8.5 | 8 | qSIS8.1, qSIS8.24, qSFW8 | SIS, SFW |
| mQTL 8.6 | 8 | qSL8 | SL |
| mQTL 8.7 | 8 | qTGW8.1s, qGY8.1s, qNar/Kr8, qKr8, qSRI-DWT8.1, qRTL8.27, qSRR8.26, qPFW-8, qRFW8 | GY, Kr, Nar/Kr, PFW, RFW, RTL, SRI-DWT, SRR, TGW, |
| mQTL 9.1 | 9 | qSIS9.8, | SIS |
| mQTL 9.2 | 9 | qNDS-9.1, qSFW9.1, qRFW9.1 | NDS, SFW, RFW |
| mQTL 9.3 | 9 | qRTL9.14 | RTL |
| mQTL 9.4 | 9 | qNDS-9.2, qSNK9, qRNK9, qSES9, qRL-9 | NDS, qSNK, RNK, SES, RL |
| mQTL 9.5 | 9 | qSTR-9 | STR |
| mQTL 9.6 | 9 | qTSP9.1s, qSR9, qSTS9, qPN9.1s, qSTW9.1s, qSHL-9, qSNK-9, qIR-9, qRFW9, qNDS-9.3, qDWT9.1, qSNC-9, qSRI-NaK9.1, qRTL9.1, qSRI-K9.1 | DWT, IR, NDS, PN, RFW, RTL, SHL, SNC, SNK, SR, SRI-K,SRI-NaK, STS, STW, TSP, |
| mQTL 9.7 | 9 | qKC9.1, qKU-9 | KC, KU |
| mQTL 9.8 | 9 | qSFW9, qRDW9, qRNC-9 | SFW, RDW, RNC |
| mQTL 9.9 | 9 | qDSS9 | DSS |
| mQTL 10.1 | 10 | qRFW10, qRDW10, qPF10.2, qSSI10, qPDS10, qSNC10, qSNK_10, qSFW10, qSDW10, qSES10.2, qSES10.1 | PDS, PF, RDW, RFW, SDW, SES,SFW, SNC, SNK, SSI, |
| mQTL 10.2 | 10 | qTGW10.1s, qDTF10.1s, qSPFR10.1s, qSKC10, qPF10.1, qSKC-10a, qSR10, qSTS10, qSKC-10b | DTF, PF, SPFR, SR, STS, TGW, |
| mQTL 10.3 | 10 | qFRSP10.1s, qSHL-10 | FRSP, SHL |
| mQTL 11.1 | 11 | qCHL11.2, qRL11.1, qSST11, qDWT11.2, qSIS11.2, qCHL11.1, qRDW11, qDSS11, qSTS11-1, qSR11-1 | CHL, DSS, DWT, RDW, RL, SIS, SR,SST, STS |
| mQTL 11.2 | 11 | qDWT11.1, qSR11-2, qSTS11-2, qSKC11, qDRW11 | SK, DRW, SR, STS, DWT |
| mQTL 11.3 | 11 | qSDW11.2, qRFW11, qSHL11.1, qSDW11.1, qSFW11.2, qSFW11.1, qRDW11.1, qLB-11, qSSI11, qRTL11.1, qPDS11 | LB, PDS, RDW, RFW, RTL, SDW, SFW, SHL, SSI, |
| mQTL 12.1 | 12 | qSFW12.1, qRL12.1, qSESF12.1, qCHL12.1, qSL12.1, qSDW12.1 | CHL, RL, SDW, SESF, SFW, SL, |
| mQTL 12.2 | 12 | qSTR-12, qCHL12.2 | STR, CHL |
| mQTL 12.3 | 12 | qCHL_12, qSUR12, qSES12, qSRI-SHL12.1, qCHL12.3, qNa12.18, qSTS12, qSR12, qSNC-12, qCHL12.4 | CHL, Na, SES, SNC, SR, RI-SHL, STS, SUR, |
| mQTL 12.4 | 12 | qSH12.1, qDSW12.1, qGY12.1, qSNK-12, qSRI-DWT12.1 | DSW, GY, SH, SNK, SRI-DWT |
| mQTL 12.5 | 12 | qSHL12.25 | SHL |
| mQTL 12.6 | 12 | qSH12.2, qSGW12.1 | SH, SGW |
| qBM: Biomass; qCDW: Coleoptile dry weight; qCFW: coleoptile fresh weight; qCHL: Chlorophyll content; qCL: Coleoptile length; qClLR: Cl^-^ in leaf at reproductive stage; qClLV: Cl- in leaf at vegetative stage; qDRW: Dry root weight; qDSS or qNDS: Days of seedling survival; qDSW or qDWT or qDWS or qDWsht or qSDW: Dry shoot weight or shoot dry weight; qDTF: Days to flowering; qFRSP: Number of fertile spikelets; qFWsht or qSFW or qDWT: shoot fresh weight; qGP: Germination percentage; qGR: Germination rate; qIR: Imbibition rate; qK or qKC: K+ concentration; qKLR: K+ in leaves at reproductive stage; qKLV: K+ in leaves at vegetative stage; qKr: Potassium in root; qKs: Potassium in Shoot; qKSV: K+ stem at vegetative stage; qKU: K+ uptake; qLBL: leaf bronzing; qNa/kLR: Na+/k+ Ratio in leaf at reproductive stage; qNa/KSV: Na+/k+ Ratio in stem at vegetative stage; qNa: Na+ concentration; qNaC: % Na content; qNaLR: Na+ in leaves at reproductive stage; qNaLV: Na+ in leaves at vegetative stage; qNar/Kr: Na+/K+ Ratio in root; qNas/Ks or qNaK or qNKR: Na^+^/K^+^ Ratio in shoot; qNas: Sodium in root; qNaSV: Na+ in stem at vegetative stage; qNU: Na+ Uptake; qPDS: Percentage damage of shoot; qPDW: Plumule dry weight; qPF1: pollen fertility; qPFW: Plumule fresh weight; qPH: Plant height; qPL: Panicle length; qPL-8: Plumule length; qPN: Number of panicles; qRDW: Root dry weight; qRDW-3: Radicle dry weight; qRDW-6, 7: Reduction of dry weight; qRFW: Root fresh weight; qRFW-6: Radicle fresh weight; qRFW-7: Reduction of fresh weight; qRKC: Root K+ concentration; qRL-1: Radicle length; qRLA: Reduction of leaf area; qRNC: Root Na concentration; qRNK: Root NaK ratio; qRNTQ: Root Na+ total quantity; qRSH: Reduction of seedling height; qRTL or qRL: Root length; qRW: Root weight; qSSI: Salinity survival index; qSES or qSIS or qSESF: Standard evaluation system score or Visual tolerance score or Salt injury score or Seedling salt injury scoreor Final SES or SES tolerance score; qSGW: Single-grain weight; qSH: sedling height; qSHL or qSL: shoot length; qSKC or qK: shoot K concentration; qSNC or qNa: shoot Na concentration; qSNK or qNaK or qSNaKR: shoot Na/K ratio; qSPFR: Spikelet fertility; qSR: Survial rate; qSRI-DWT: relative dry weight compared to control; qSRI-K: relative shoot potassium concentration compared to control; qSRI-Na: relative shoot sodium concentration compared to control; qSRI-RTL: relative root length compared to control; qSRR: Shoot root ratio; qSST: Score of salt toxicity; qSTR: standard tolerance ranking; qSTS: salt tolerance score; qSTSP: Number of sterile spikelets; qSTW: Straw dry weight; qSUR: Seedling survival; qTGW: 1000 grain weight; qTN: Tiller number; qTSP: Total spikelets number; Saltol: salinity tolerance at seedling stage; saltol-qNU-KU-NKR: Saltol ion uptake and Na/K ratio | | | |
