# Supplementary material for: Salt tolerance in rice: seedling and reproductive stage QTL mapping come of age
Source: Theor Appl Genet. 2021 Jul 21;134(11):3495–533. doi: 10.1007/s00122-021-03890-3 (PMC8519845; doi:10.1007/s00122-021-03890-3)
Supplement: Supplementary file 3 — Supplementary file3 (DOCX 52 kb) [file 122_2021_3890_MOESM3_ESM.docx]

**Online Resource 3: List of Putative Candidate genes associated with Meta-QTLs of rice**

| **S.No** | **Gene ID** | **Gene function** | **Meta QTL** |
| --- | --- | --- | --- |
| 1 | LOC_Os01g18660 | aminotransferase, putative, expressed | mQTL 1.1 |
| 2 | LOC_Os01g18640 | aminotransferase, putative, expressed | mQTL 1.1 |
| 3 | LOC_Os01g18170 | Cupin domain containing protein, expressed | mQTL 1.1 |
| 4 | LOC_Os01g15120 | hydrolase, alpha/beta fold family domain containing protein, expressed | mQTL 1.1 |
| 5 | LOC_Os01g15130 | hydrolase, alpha/beta fold family domain containing protein, expressed | mQTL 1.1 |
| 6 | LOC_Os01g20160 | OsHKT1;5 - Na+ transporter, expressed | mQTL 1.1 |
| 7 | LOC_Os01g18850 | OsSPL1 - SBP-box gene family member, expressed | mQTL 1.1 |
| 8 | LOC_Os01g17160 | OsSubt1 - Putative Subtilisin homologue, expressed | mQTL 1.1 |
| 9 | LOC_Os01g20880 | OsWAK3 - OsWAK receptor-like cytoplasmic kinase OsWAK-RLCK, expressed | mQTL 1.1 |
| 10 | LOC_Os01g20900 | OsWAK4 - OsWAK receptor-like cytoplasmic kinase OsWAK-RLCK, expressed | mQTL 1.1 |
| 11 | LOC_Os01g19440 | pectinesterase, putative, expressed | mQTL 1.1 |
| 12 | LOC_Os01g20980 | pectinesterase, putative, expressed | mQTL 1.1 |
| 13 | LOC_Os01g21034 | pectinesterase, putative, expressed | mQTL 1.1 |
| 14 | LOC_Os01g15039 | pectinesterase, putative, expressed | mQTL 1.1 |
| 15 | LOC_Os01g21034 | pectinesterase, putative, expressed | mQTL 1.1 |
| 16 | LOC_Os01g15810 | peroxidase precursor, putative, expressed | mQTL 1.1 |
| 17 | LOC_Os01g15830 | peroxidase precursor, putative, expressed | mQTL 1.1 |
| 18 | LOC_Os01g18910 | peroxidase precursor, putative, expressed | mQTL 1.1 |
| 19 | LOC_Os01g18930 | peroxidase precursor, putative, expressed | mQTL 1.1 |
| 20 | LOC_Os01g18950 | peroxidase precursor, putative, expressed | mQTL 1.1 |
| 21 | LOC_Os01g18970 | peroxidase precursor, putative, expressed | mQTL 1.1 |
| 22 | LOC_Os01g16450 | peroxidase precursor, putative, expressed | mQTL 1.1 |
| 23 | LOC_Os01g18890 | peroxidase precursor, putative, expressed | mQTL 1.1 |
| 24 | LOC_Os01g19020 | peroxidase precursor, putative, expressed | mQTL 1.1 |
| 25 | LOC_Os01g18584 | WRKY9, expresse | mQTL 1.1 |
| 26 | LOC_Os01g27720 | E3 ubiquitin-protein ligase MGRN1, putative, expressed | mQTL 1.2 |
| 27 | LOC_Os01g22640 | GDSL-like lipase/acylhydrolase, putative, expressed | mQTL 1.2 |
| 28 | LOC_Os01g22660 | GDSL-like lipase/acylhydrolase, putative, expressed | mQTL 1.2 |
| 29 | LOC_Os01g22780 | GDSL-like lipase/acylhydrolase, putative, expressed | mQTL 1.2 |
| 30 | LOC_Os01g24590 | LTPL46 - Protease inhibitor/seed storage/LTP family protein precursor, expressed | mQTL 1.2 |
| 31 | LOC_Os01g26174 | OsWAK5 - OsWAK receptor-like protein kinase, expressed | mQTL 1.2 |
| 32 | LOC_Os01g26210 | OsWAK6 - OsWAK receptor-like protein kinase, expressed | mQTL 1.2 |
| 33 | LOC_Os01g26270 | OsWAK7 - OsWAK receptor-like protein kinase, expressed | mQTL 1.2 |
| 34 | LOC_Os01g26280 | OsWAK8 - OsWAK receptor-like protein kinase, expressed | mQTL 1.2 |
| 35 | LOC_Os01g26300 | OsWAK9 - OsWAK receptor-like cytoplasmic kinase OsWAK-RLCK, expressed | mQTL 1.2 |
| 36 | LOC_Os01g20980 | pectinesterase, putative, expressed | mQTL 1.2 |
| 37 | LOC_Os01g21034 | pectinesterase, putative, expressed | mQTL 1.2 |
| 38 | LOC_Os01g22336 | peroxidase precursor, putative, expressed | mQTL 1.2 |
| 39 | LOC_Os01g28030 | peroxidase precursor, putative, expressed | mQTL 1.2 |
| 40 | LOC_Os01g22249 | peroxidase precursor, putative, expressed | mQTL 1.2 |
| 41 | LOC_Os01g22352 | peroxidase precursor, putative, expressed | mQTL 1.2 |
| 42 | LOC_Os01g22370 | peroxidase precursor, putative, expressed | mQTL 1.2 |
| 43 | LOC_Os01g22230 | peroxidase precursor, putative, expressed | mQTL 1.2 |
| 44 | LOC_Os01g27170 | potassium transporter, putative, expressed | mQTL 1.2 |
| 45 | LOC_Os01g24750 | Ser/Thr protein phosphatase family protein, putative, expressed | mQTL 1.2 |
| 46 | LOC_Os01g22954 | serine carboxypeptidase, putative, expressed | mQTL 1.2 |
| 47 | LOC_Os01g21960 | serine/threonine-protein kinase, putative, expressed | mQTL 1.2 |
| 48 | LOC_Os01g26310 | wall-associated kinase-like 2, putative, expressed | mQTL 1.2 |
| 49 | LOC_Os01g40420 | CBS domain containing membrane protein, putative, expressed | mQTL 1.3 |
| 50 | LOC_Os01g40840 | serine/threonine-protein kinase AFC3, putative, expressed | mQTL 1.3 |
| 51 | LOC_Os01g40430 | WRKY27, expressed | mQTL 1.3 |
| 52 | LOC_Os01g40260 | WRKY77, expressed | mQTL 1.3 |
| 53 | LOC_Os01g55040 | 1,3-beta-glucan synthase component, putative, expressed | mQTL 1.4 |
| 54 | LOC_Os01g50100 | ABC transporter, ATP-binding protein, putative, expressed | mQTL 1.4 |
| 55 | LOC_Os01g52550 | ABC transporter, ATP-binding protein, putative, expressed | mQTL 1.4 |
| 56 | LOC_Os01g56400 | ABC transporter, ATP-binding protein, putative, expressed | mQTL 1.4 |
| 57 | LOC_Os01g53450 | aminotransferase, classes I and II, domain containing protein, expressed | mQTL 1.4 |
| 58 | LOC_Os01g55540 | aminotransferase, classes I and II, domain containing protein, expressed | mQTL 1.4 |
| 59 | LOC_Os01g51780 | auxin efflux carrier component, putative, expressed | mQTL 1.4 |
| 60 | LOC_Os01g58860 | auxin efflux carrier component, putative, expressed | mQTL 1.4 |
| 61 | LOC_Os01g60230 | auxin efflux carrier component, putative, expressed | mQTL 1.4 |
| 62 | LOC_Os01g54990 | auxin response factor, putative, expressed | mQTL 1.4 |
| 63 | LOC_Os01g58910 | auxin-induced protein 5NG4, putative, expressed | mQTL 1.4 |
| 64 | LOC_Os01g50860 | chloride transporter, chloride channel family, putative, expressed | mQTL 1.4 |
| 65 | LOC_Os01g50900 | Cupin domain containing protein, expressed | mQTL 1.4 |
| 66 | LOC_Os01g60110 | E2F-related protein, putative, expressed | mQTL 1.4 |
| 67 | LOC_Os01g49470 | E3 ubiquitin ligase, putative, expressed | mQTL 1.4 |
| 68 | LOC_Os01g54890 | ethylene-responsive transcription factor 2, putative, expressed | mQTL 1.4 |
| 69 | LOC_Os01g52770 | GDSL-like lipase/acylhydrolase, putative, expressed | mQTL 1.4 |
| 70 | LOC_Os01g54470 | GDSL-like lipase/acylhydrolase, putative, expressed | mQTL 1.4 |
| 71 | LOC_Os01g51060 | hydrolase, putative, expressed | mQTL 1.4 |
| 72 | LOC_Os01g49640 | LTPL149 - Protease inhibitor/seed storage/LTP family protein precursor, expressed | mQTL 1.4 |
| 73 | LOC_Os01g49650 | LTPL150 - Protease inhibitor/seed storage/LTP family protein precursor, expressed | mQTL 1.4 |
| 74 | LOC_Os01g58660 | LTPL29 - Protease inhibitor/seed storage/LTP family protein precursor, expressed | mQTL 1.4 |
| 75 | LOC_Os01g58650 | LTPL35 - Protease inhibitor/seed storage/LTP family protein precursor, putative | mQTL 1.4 |
| 76 | LOC_Os01g59870 | LTPL65 - Protease inhibitor/seed storage/LTP family protein precursor, expressed | mQTL 1.4 |
| 77 | LOC_Os01g49120 | MATE efflux family protein, putative, expressed | mQTL 1.4 |
| 78 | LOC_Os01g56050 | MATE efflux family protein, putative, expressed | mQTL 1.4 |
| 79 | LOC_Os01g58080 | membrane-associated salt-inducible protein, putative, expressed | mQTL 1.4 |
| 80 | LOC_Os01g54490 | osFTL9 FT-Like9 homologous to Flowering Locus T gene; contains Pfam profile PF01161: Phosphatidylethanolamine-binding protein | mQTL 1.4 |
| 81 | LOC_Os01g56240 | OsSAUR2 - Auxin-responsive SAUR gene family member, expressed | mQTL 1.4 |
| 82 | LOC_Os01g50680 | OsSub2 - Putative Subtilisin homologue, expresse | mQTL 1.4 |
| 83 | LOC_Os01g52750 | OsSub3 - Putative Subtilisin homologue, expressed | mQTL 1.4 |
| 84 | LOC_Os01g56320 | OsSub4 - Putative Subtilisin homologue, expressed | mQTL 1.4 |
| 85 | LOC_Os01g58240 | OsSub6 - Putative Subtilisin homologue, expressed | mQTL 1.4 |
| 86 | LOC_Os01g58270 | OsSub7 - Putative Subtilisin homologue, expressed | mQTL 1.4 |
| 87 | LOC_Os01g58280 | OsSub8 - Putative Subtilisin homologue, expressed | mQTL 1.4 |
| 88 | LOC_Os01g58290 | OsSub9 - Putative Subtilisin homologue, expressed | mQTL 1.4 |
| 89 | LOC_Os01g49529 | OsWAK10d - OsWAK receptor-like cytoplasmic kinase OsWAK-RLCK, expressed | mQTL 1.4 |
| 90 | LOC_Os01g53990 | pectinesterase, putative, expressed | mQTL 1.4 |
| 91 | LOC_Os01g57854 | pectinesterase, putative, expressed | mQTL 1.4 |
| 92 | LOC_Os01g57730 | peroxidase precursor, putative, expressed | mQTL 1.4 |
| 93 | LOC_Os01g52070 | potassium channel AKT1, putative, expressed | mQTL 1.4 |
| 94 | LOC_Os01g55200 | potassium channel KAT1, putative, expressed | mQTL 1.4 |
| 95 | LOC_Os01g49690 | Ser/Thr protein phosphatase family protein, putative, expressed | mQTL 1.4 |
| 96 | LOC_Os01g52260 | serine acetyltransferase protein, putative, expressed | mQTL 1.4 |
| 97 | LOC_Os01g54480 | serine/threonine protein kinase, putative, expressed | mQTL 1.4 |
| 98 | LOC_Os01g57480 | serine/threonine-protein kinase receptor precursor, putative, expressed | mQTL 1.4 |
| 99 | LOC_Os01g57560 | serine/threonine-protein kinase receptor precursor, putative, expressed | mQTL 1.4 |
| 100 | LOC_Os01g48874 | wax synthase, putative, expressed | mQTL 1.4 |
| 101 | LOC_Os01g56360 | wax synthase, putative, expressed | mQTL 1.4 |
| 102 | LOC_Os01g56370 | wax synthase, putative, expressed | mQTL 1.4 |
| 103 | LOC_Os01g54600 | WRKY13, expressed | mQTL 1.4 |
| 104 | LOC_Os01g53040 | WRKY14, expressed | mQTL 1.4 |
| 105 | LOC_Os01g53260 | WRKY23, expressed | mQTL 1.4 |
| 106 | LOC_Os01g51690 | WRKY26, expressed | mQTL 1.4 |
| 107 | LOC_Os01g50100 | ABC transporter, ATP-binding protein, putative, expressed | mQTL 1.5 |
| 108 | LOC_Os01g52550 | ABC transporter, ATP-binding protein, putative, expressed | mQTL 1.5 |
| 109 | LOC_Os01g51780 | auxin efflux carrier component, putative, expressed | mQTL 1.5 |
| 110 | LOC_Os01g50860 | chloride transporter, chloride channel family, putative, expressed | mQTL 1.5 |
| 111 | LOC_Os01g50900 | Cupin domain containing protein, expressed | mQTL 1.5 |
| 112 | LOC_Os01g51060 | hydrolase, putative, expressed | mQTL 1.5 |
| 113 | LOC_Os01g50680 | OsSub2 - Putative Subtilisin homologue, expressed | mQTL 1.5 |
| 114 | LOC_Os01g52070 | potassium channel AKT1, putative, expressed | mQTL 1.5 |
| 115 | LOC_Os01g49690 | Ser/Thr protein phosphatase family protein, putative, expressed | mQTL 1.5 |
| 116 | LOC_Os01g52260 | serine acetyltransferase protein, putative, expressed | mQTL 1.5 |
| 117 | LOC_Os01g51690 | WRKY26, expressed | mQTL 1.5 |
| 118 | LOC_Os01g65090 | aminotransferase, classes I and II, domain containing protein, expressed | mQTL 1.6 |
| 119 | LOC_Os01g62070 | cation efflux family protein, putative, expressed | mQTL 1.6 |
| 120 | LOC_Os01g65500 | chloride channel protein, putative, expressed | mQTL 1.6 |
| 121 | LOC_Os01g62010 | hydrolase, alpha/beta fold family domain containing protein, expressed | mQTL 1.6 |
| 122 | LOC_Os01g65070 | hydrolase, alpha/beta fold family domain containing protein, expressed | mQTL 1.6 |
| 123 | LOC_Os01g63990 | hydrolase, alpha/beta fold family protein, putative, expressed | mQTL 1.6 |
| 124 | LOC_Os01g64262 | hydrolase, alpha/beta fold family, putative, expressed | mQTL 1.6 |
| 125 | LOC_Os01g62980 | LTPL101 - Protease inhibitor/seed storage/LTP family protein precursor, expressed | mQTL 1.6 |
| 126 | LOC_Os01g64850 | OsSub10 - Putative Subtilisin homologue, expressed | mQTL 1.6 |
| 127 | LOC_Os01g64860 | OsSub11 - Putative Subtilisin homologue, expressed | mQTL 1.6 |
| 128 | LOC_Os01g62860 | oxidoreductase, aldo/keto reductase family protein, putative, expresse | mQTL 1.6 |
| 129 | LOC_Os01g62870 | oxidoreductase, aldo/keto reductase family protein, putative, expressed | mQTL 1.6 |
| 130 | LOC_Os01g62880 | oxidoreductase, aldo/keto reductase family protein, putative, expressed | mQTL 1.6 |
| 131 | LOC_Os01g65410 | serine hydroxymethyltransferase, mitochondrial precursor, putative, expressed | mQTL 1.6 |
| 132 | LOC_Os01g62080 | serine/threonine-protein kinase AFC1, putative, expressed | mQTL 1.6 |
| 133 | LOC_Os01g65010 | serine/threonine-protein kinase receptor precursor, putative, expressed | mQTL 1.6 |
| 134 | LOC_Os01g62510 | WRKY119, expressed | mQTL 1.6 |
| 135 | LOC_Os01g62514 | WRKY56, expressed | mQTL 1.6 |
| 136 | LOC_Os01g73530 | ABC transporter, ATP-binding protein, putative, expressed | mQTL 1.7 |
| 137 | LOC_Os01g74470 | ABC transporter, ATP-binding protein, putative, expressed | mQTL 1.7 |
| 138 | LOC_Os01g73480 | acetyltransferase, GNAT family protein, expressed | mQTL 1.7 |
| 139 | LOC_Os01g73040 | CBS domain-containing protein, putative, expressed | mQTL 1.7 |
| 140 | LOC_Os01g74480 | cupin domain containing protein, expressed | mQTL 1.7 |
| 141 | LOC_Os01g72850 | GDSL-motif lipase/hydrolase family protein, putative, expressed | mQTL 1.7 |
| 142 | LOC_Os01g73190 | peroxidase precursor, putative, expressed | mQTL 1.7 |
| 143 | LOC_Os01g73200 | peroxidase precursor, putative, expressed | mQTL 1.7 |
| 144 | LOC_Os01g73220 | peroxidase precursor, putative, expressed | mQTL 1.7 |
| 145 | LOC_Os01g73170 | peroxidase precursor, putative, expressed | mQTL 1.7 |
| 146 | LOC_Os01g74140 | WRKY17, expressed | mQTL 1.7 |
| 233 | LOC_Os02g19924 | aminotransferase, classes I and II, domain containing protein, expressed | mQTL 2.1 |
| 234 | LOC_Os02g19970 | aminotransferase, classes I and II, domain containing protein, expressed | mQTL 2.1 |
| 235 | LOC_Os02g20320 | OsSAUR7 - Auxin-responsive SAUR gene family member, expressed | mQTL 2.1 |
| 236 | LOC_Os02g42640 | CBS domain-containing protein, putative, expressed | mQTL 2.2 |
| 237 | LOC_Os02g42374 | e2f-associated phosphoprotein, putative, expressed | mQTL 2.2 |
| 238 | LOC_Os02g43340 | hydrolase, alpha/beta fold family domain containing protein, expressed | mQTL 2.2 |
| 239 | LOC_Os02g42210 | membrane-associated salt-inducible protein like, putative, expressed | mQTL 2.2 |
| 240 | LOC_Os02g42990 | OsSAUR11 - Auxin-responsive SAUR gene family member, expressed | mQTL 2.2 |
| 241 | LOC_Os02g42150 | OsWAK14 - OsWAK receptor-like protein kinase, expressed | mQTL 2.2 |
| 242 | LOC_Os02g42160 | OsWAK15 - OsWAK receptor-like protein kinase, expressed | mQTL 2.2 |
| 243 | LOC_Os02g42190 | OsWAK16 - OsWAK receptor-like protein kinase, expressed | mQTL 2.2 |
| 244 | LOC_Os02g43110 | sodium/calcium exchanger 1 precursor, putative, expressed | mQTL 2.2 |
| 245 | LOC_Os02g42110 | wall-associated receptor kinase-like 22 precursor, putative, expressed | mQTL 2.2 |
| 246 | LOC_Os03g16110 | Ser/Thr protein phosphatase family protein, putative, expressed | mQTL 3.1 |
| 247 | LOC_Os03g19670 | GDSL-like lipase/acylhydrolase, putative, expressed | mQTL 3.2 |
| 248 | LOC_Os03g19610 | pectinesterase, putative, expressed | mQTL 3.2 |
| 249 | LOC_Os03g20170 | ABC transporter, ATP-binding protein, putative, expressed | mQTL 3.3 |
| 250 | LOC_Os03g17180 | ABC transporter, ATP-binding protein, putative, expressed | mQTL 3.3 |
| 251 | LOC_Os03g21490 | ABC transporter, ATP-binding protein, putative, expressed | mQTL 3.3 |
| 252 | LOC_Os03g18810 | aminotransferase, classes I and II, domain containing protein, expressed | mQTL 3.3 |
| 253 | LOC_Os03g21090 | auxin-independent growth promoter protein, putative, expressed | mQTL 3.3 |
| 254 | LOC_Os03g17700 | CGMC_MAPKCGMC_2_ERK.2 - CGMC includes CDA, MAPK, GSK3, and CLKC kinases, expressed | mQTL 3.3 |
| 255 | LOC_Os03g19670 | GDSL-like lipase/acylhydrolase, putative, expressed | mQTL 3.3 |
| 256 | LOC_Os03g17900 | hydrolase, alpha/beta fold family protein, putative, expressed | mQTL 3.3 |
| 257 | LOC_Os03g20760 | LTPL66 - Protease inhibitor/seed storage/LTP family protein precursor, expressed | mQTL 3.3 |
| 258 | LOC_Os03g17790 | OsRCI2-5 - Putative low temperature and salt responsive protein, expressed | mQTL 3.3 |
| 259 | LOC_Os03g18050 | OsSAUR13 - Auxin-responsive SAUR gene family member, expressed | mQTL 3.3 |
| 260 | LOC_Os03g17610 | OsTOP6A3 - Topoisomerase 6 subunit A homolog 3, expressed | mQTL 3.3 |
| 261 | LOC_Os03g18860 | pectinesterase, putative, expressed | mQTL 3.3 |
| 262 | LOC_Os03g19610 | pectinesterase, putative, expressed | mQTL 3.3 |
| 263 | LOC_Os03g17550 | serine/threonine-protein kinase, putative, expressed | mQTL 3.3 |
| 264 | LOC_Os03g20550 | WRKY55, expressed | mQTL 3.3 |
| 265 | LOC_Os03g21960 | aminotransferase, putative, expressed | mQTL 3.4 |
| 266 | LOC_Os03g22450 | auxin response factor 75, putative, expressed | mQTL 3.4 |
| 267 | LOC_Os03g22270 | auxin-repressed protein, putative, expressed | mQTL 3.4 |
| 268 | LOC_Os03g22550 | cation efflux family protein, putative, expressed | mQTL 3.4 |
| 269 | LOC_Os03g21790 | cupin domain containing protein, expressed | mQTL 3.4 |
| 270 | LOC_Os03g24300 | LTPL1 - Protease inhibitor/seed storage/LTP family protein precursor, expressed | mQTL 3.4 |
| 271 | LOC_Os03g22010 | peroxidase precursor, putative, expressed | mQTL 3.4 |
| 272 | LOC_Os03g22020 | peroxidase precursor, putative, expressed | mQTL 3.4 |
| 273 | LOC_Os03g21890 | potassium transporter, putative, expressed | mQTL 3.4 |
| 274 | LOC_Os03g37411 | MATE efflux family protein, putative, expressed | mQTL 3.5 |
| 275 | LOC_Os03g36730 | OST3/OST6 family protein, putative, expressed | mQTL 3.5 |
| 276 | LOC_Os03g36560 | peroxidase precursor, putative, expressed | mQTL 3.5 |
| 277 | LOC_Os03g54790 | ABC transporter, ATP-binding protein, putative, expressed | mQTL 3.6 |
| 278 | LOC_Os03g58030 | acetyltransferase, GNAT family, putative, expressed | mQTL 3.6 |
| 279 | LOC_Os03g58010 | acetyltransferase, GNAT family, putative, expressed | mQTL 3.6 |
| 280 | LOC_Os03g58020 | acetyltransferase, GNAT family, putative, expressed | mQTL 3.6 |
| 281 | LOC_Os03g56800 | CBS domain containing membrane protein, putative, expressed | mQTL 3.6 |
| 282 | LOC_Os03g57960 | cupin domain containing protein, expressed | mQTL 3.6 |
| 283 | LOC_Os03g58980 | Cupin domain containing protein, expressed | mQTL 3.6 |
| 284 | LOC_Os03g58990 | cupin domain containing protein, expressed | mQTL 3.6 |
| 285 | LOC_Os03g59010 | Cupin domain containing protein, expressed | mQTL 3.6 |
| 286 | LOC_Os03g59580 | hydrolase, NUDIX family, domain containing protein, expressed | mQTL 3.6 |
| 287 | LOC_Os03g58670 | LTPL119 - Protease inhibitor/seed storage/LTP family protein precursor, expressed | mQTL 3.6 |
| 288 | LOC_Os03g59380 | LTPL28 - Protease inhibitor/seed storage/LTP family protein precursor, expressed | mQTL 3.6 |
| 289 | LOC_Os03g57970 | LTPL73 - Protease inhibitor/seed storage/LTP family protein precursor, expressed | mQTL 3.6 |
| 290 | LOC_Os03g57990 | LTPL74 - Protease inhibitor/seed storage/LTP family protein precursor, expressed | mQTL 3.6 |
| 291 | LOC_Os03g58940 | LTPL83 - Protease inhibitor/seed storage/LTP family protein precursor, putative, expressed | mQTL 3.6 |
| 292 | LOC_Os03g57980 | LTPL99 - Protease inhibitor/seed storage/LTP family protein precursor, expressed | mQTL 3.6 |
| 293 | LOC_Os03g55640 | OsSigP2 - Putative Type I Signal Peptidase homologue; employs a putative Ser/Lys catalytic dyad, expressed | mQTL 3.6 |
| 294 | LOC_Os03g55350 | OsSub31 - Putative Subtilisin homologue, expressed | mQTL 3.6 |
| 295 | LOC_Os03g54091 | OsTOP6A1 - Topoisomerase 6 subunit A homolog 1, expressed | mQTL 3.6 |
| 296 | LOC_Os03g55410 | peroxidase precursor, putative, expressed | mQTL 3.6 |
| 297 | LOC_Os03g55420 | peroxidase precursor, putative, expressed | mQTL 3.6 |
| 298 | LOC_Os03g54100 | potassium channel protein, putative, expressed | mQTL 3.6 |
| 299 | LOC_Os03g52840 | serine hydroxymethyltransferase, mitochondrial precursor, putative, expressed | mQTL 3.6 |
| 300 | LOC_Os03g53880 | Serine/threonine-protein kinase TOUSLED, putative, expressed | mQTL 3.6 |
| 301 | LOC_Os03g59450 | transporter-related, putative, expressed | mQTL 3.6 |
| 302 | LOC_Os03g53050 | WRKY121, expressed | mQTL 3.6 |
| 303 | LOC_Os03g55080 | WRKY3, expressed | mQTL 3.6 |
| 304 | LOC_Os03g55164 | WRKY4, expressed | mQTL 3.6 |
| 305 | LOC_Os03g58420 | WRKY6, expressed | mQTL 3.6 |
| 306 | LOC_Os04g03710 | OsSub36 - Putative Subtilisin homologue, expressed | mQTL 4.1 |
| 307 | LOC_Os04g03796 | OsSub37 - Putative Subtilisin homologue, expressed | mQTL 4.1 |
| 308 | LOC_Os04g03810 | OsSub38 - Putative Subtilisin homologue, expressed | mQTL 4.1 |
| 309 | LOC_Os04g03850 | OsSub39 - Putative Subtilisin homologue, expressed | mQTL 4.1 |
| 310 | LOC_Os04g03830 | OsWAK29 - OsWAK receptor-like protein kinase, expressed | mQTL 4.1 |
| 311 | LOC_Os04g51140 | E2F-related protein, putative, expressed | mQTL 4.10 |
| 312 | LOC_Os04g51040 | OsWAK50 - OsWAK receptor-like protein kinase, expressed | mQTL 4.10 |
| 313 | LOC_Os04g51009 | OsWAK52 - OsWAK short gene, expressed | mQTL 4.10 |
| 314 | LOC_Os04g51050 | OsWAK53b - OsWAK receptor-like protein kinase, expressed | mQTL 4.10 |
| 315 | LOC_Os04g51300 | peroxidase precursor, putative, expressed | mQTL 4.10 |
| 316 | LOC_Os04g51030 | wall-associated kinase 1, putative, expressed | mQTL 4.10 |
| 317 | LOC_Os04g50920 | WRKY37, expressed | mQTL 4.10 |
| 318 | LOC_Os04g51560 | WRKY68, expressed | mQTL 4.10 |
| 319 | LOC_Os04g20680 | wall-associated receptor kinase 3 precursor, putative, expressed | mQTL 4.5 |
| 320 | LOC_Os04g20880 | wax synthase isoform 1, putative, expressed | mQTL 4.5 |
| 321 | LOC_Os04g18790 | OsFBX126 - F-box domain containing protein, expressed | mQTL 4.5 |
| 322 | LOC_Os04g27060 | oxidoreductase, aldo/keto reductase family protein, putative, expressed | mQTL 4.6 |
| 323 | LOC_Os04g33700 | ABC transmembrane transporter domain containing protein, expressed | mQTL 4.7 |
| 324 | LOC_Os04g34600 | abscisic stress-ripening, putative, expressed | mQTL 4.7 |
| 325 | LOC_Os04g33950 | E2F family transcription factor protein, putative, expressed | mQTL 4.7 |
| 326 | LOC_Os04g33590 | hydrolase, alpha/beta fold family protein, putative, expressed | mQTL 4.7 |
| 327 | LOC_Os04g33600 | hydrolase, alpha/beta fold family protein, putative, expressed | mQTL 4.7 |
| 328 | LOC_Os04g33920 | LTPL102 - Protease inhibitor/seed storage/LTP family protein precursor, expressed | mQTL 4.7 |
| 329 | LOC_Os04g33930 | LTPL103 - Protease inhibitor/seed storage/LTP family protein precursor, expressed | mQTL 4.7 |
| 330 | LOC_Os04g34630 | peroxidase precursor, putative, expressed | mQTL 4.7 |
| 331 | LOC_Os04g33570 | RCN4 Centroradialis-like1 homologous to TFL1 gene; contains Pfam profile PF01161: Phosphatidylethanolamine-binding protein, expressed | mQTL 4.7 |
| 332 | LOC_Os04g33470 | Ser/Thr protein phosphatase family protein, putative, expressed | mQTL 4.7 |
| 333 | LOC_Os04g33530 | Ser/Thr protein phosphatase family protein, putative, expressed | mQTL 4.7 |
| 334 | LOC_Os04g34250 | serine/threonine-protein kinase receptor precursor, putative, expressed | mQTL 4.7 |
| 335 | LOC_Os04g34330 | serine/threonine-protein kinase receptor precursor, putative, expressed | mQTL 4.7 |
| 336 | LOC_Os04g34360 | serine/threonine-protein kinase receptor precursor, putative, expressed | mQTL 4.7 |
| 337 | LOC_Os04g34390 | serine/threonine-protein kinase receptor precursor, putative, expressed | mQTL 4.7 |
| 338 | LOC_Os04g34410 | serine/threonine-protein kinase receptor precursor, putative, expressed | mQTL 4.7 |
| 339 | LOC_Os04g34420 | serine/threonine-protein kinase receptor precursor, putative, expressed | mQTL 4.7 |
| 340 | LOC_Os04g34270 | serine/threonine-protein kinase receptor precursor, putative, expressed | mQTL 4.7 |
| 341 | LOC_Os04g34300 | serine/threonine-protein kinase receptor precursor, putative, expressed | mQTL 4.7 |
| 342 | LOC_Os04g34370 | serine/threonine-protein kinase receptor precursor, putative, expressed | mQTL 4.7 |
| 343 | LOC_Os04g40570 | ABC transporter, ATP-binding protein, putative, expressed | mQTL 4.8 |
| 344 | LOC_Os04g39140 | acetyltransferase, GNAT family, putative, expressed | mQTL 4.8 |
| 345 | LOC_Os04g37580 | cation transport regulator-like protein 1, putative, expressed | mQTL 4.8 |
| 346 | LOC_Os04g38840 | LTPL81 - Protease inhibitor/seed storage/LTP family protein precursor, expressed | mQTL 4.8 |
| 347 | LOC_Os04g37470 | oxidoreductase, aldo/keto reductase family protein, putative, expressed | mQTL 4.8 |
| 348 | LOC_Os04g37480 | oxidoreductase, aldo/keto reductase family protein, putative, expressed | mQTL 4.8 |
| 349 | LOC_Os04g37490 | oxidoreductase, aldo/keto reductase family protein, putative, expressed | mQTL 4.8 |
| 350 | LOC_Os04g38560 | pectinesterase, putative, expressed | mQTL 4.8 |
| 351 | LOC_Os04g39100 | peroxidase precursor, putative, expressed | mQTL 4.8 |
| 352 | LOC_Os04g37710 | serine hydrolase domain containing protein, expressed | mQTL 4.8 |
| 353 | LOC_Os04g40590 | wax synthase isoform 1, putative, expressed | mQTL 4.8 |
| 354 | LOC_Os04g39570 | WRKY35, expressed | mQTL 4.8 |
| 355 | LOC_Os04g45370 | OsSAUR19 - Auxin-responsive SAUR gene family member, expressed | mQTL 4.9 |
| 356 | LOC_Os05g07210 | metal cation transporter, putative, expressed | mQTL 5.2 |
| 357 | LOC_Os05g07300 | Serine/threonine-protein kinase receptor precursor, putative, expressed | mQTL 5.2 |
| 358 | LOC_Os05g36010 | OsSub47 - Putative Subtilisin homologue, expressed | mQTL 5.5 |
| 359 | LOC_Os05g36050 | serine/threonine-protein kinase, putative, expressed | mQTL 5.5 |
| 360 | LOC_Os06g24730 | hydrolase, alpha/beta fold family domain containing protein, expressed | mQTL 6.1 |
| 361 | LOC_Os07g12530 | ABC1 family domain containing protein, putative, expressed | mQTL 7.1 |
| 362 | LOC_Os07g11630 | LTPL163 - Protease inhibitor/seed storage/LTP family protein precursor, expressed | mQTL 7.1 |
| 363 | LOC_Os07g11650 | LTPL164 - Protease inhibitor/seed storage/LTP family protein precursor, expressed | mQTL 7.1 |
| 364 | LOC_Os07g12090 | LTPL165 - Protease inhibitor/seed storage/LTP family protein precursor, expressed | mQTL 7.1 |
| 365 | LOC_Os07g12080 | LTPL169 - Protease inhibitor/seed storage/LTP family protein precursor, expressed | mQTL 7.1 |
| 366 | LOC_Os07g28090 | ABC transporter, ATP-binding protein, putative, expressed | mQTL 7.2 |
| 367 | LOC_Os07g27780 | aminotransferase, putative, expressed | mQTL 7.2 |
| 368 | LOC_Os07g27940 | LTPL27 - Protease inhibitor/seed storage/LTP family protein precursor, putative, expressed | mQTL 7.2 |
| 369 | LOC_Os07g27460 | serine/threonine-protein kinase 19, putative, expressed | mQTL 7.2 |
| 370 | LOC_Os07g27670 | WRKY115, expressed | mQTL 7.2 |
| 371 | LOC_Os08g02094 | GDSL-like lipase/acylhydrolase, putative, expressed | mQTL 8.1 |
| 372 | LOC_Os08g02520 | OsSAUR31 - Auxin-responsive SAUR gene family member, expressed | mQTL 8.1 |
| 373 | LOC_Os08g02530 | OsSAUR32 - Auxin-responsive SAUR gene family member, expressed | mQTL 8.1 |
| 374 | LOC_Os08g02110 | peroxidase precursor, putative, expressed | mQTL 8.1 |
| 375 | LOC_Os08g20570 | chloride channel protein, putative, expressed | mQTL 8.2 |
| 376 | LOC_Os08g17400 | WRKY89, expressed | mQTL 8.2 |
| 377 | LOC_Os08g27674 | LTPL130 - Protease inhibitor/seed storage/LTP family protein precursor, putative, expressed | mQTL 8.4 |
| 378 | LOC_Os08g27210 | LTPL3 - Protease inhibitor/seed storage/LTP family protein precursor, putative, expressed | mQTL 8.4 |
| 379 | LOC_Os08g27810 | OsWAK115 - OsWAK receptor-like protein OsWAK-RLP, expressed | mQTL 8.4 |
| 380 | LOC_Os08g27780 | OsWAK77 - OsWAK receptor-like cytoplasmic kinase OsWAK-RLCK, expressed | mQTL 8.4 |
| 381 | LOC_Os08g35190 | auxin-repressed protein, putative, expressed | mQTL 8.5 |
| 382 | LOC_Os08g35750 | Cupin domain containing protein, expressed | mQTL 8.5 |
| 383 | LOC_Os08g35760 | Cupin domain containing protein, expressed | mQTL 8.5 |
| 384 | LOC_Os08g35480 | cupin superfamily protein, putative, expressed | mQTL 8.5 |
| 385 | LOC_Os08g35665 | LTPL48 - Protease inhibitor/seed storage/LTP family protein precursor, expressed | mQTL 8.5 |
| 386 | LOC_Os08g37432 | MATE efflux family protein, putative, expressed | mQTL 8.5 |
| 387 | LOC_Os08g36420 | metal cation transporter, putative, expressed | mQTL 8.5 |
| 388 | LOC_Os08g36340 | potassium transporter, putative, expressed | mQTL 8.5 |
| 389 | LOC_Os08g36550 | salt tolerant protein, putative, expressed | mQTL 8.5 |
| 390 | LOC_Os08g35440 | Ser/Thr protein phosphatase family protein, putative, expressed | mQTL 8.5 |
| 391 | LOC_Os08g40900 | auxin response factor, putative, expressed | mQTL 8.6 |
| 392 | LOC_Os08g39890 | OsSPL14 - SBP-box gene family member, expressed | mQTL 8.6 |
| 393 | LOC_Os08g40260 | OsSPL15 - SBP-box gene family member, expressed | mQTL 8.6 |
| 394 | LOC_Os08g39950 | potassium transporter, putative, expressed | mQTL 8.6 |
| 395 | LOC_Os08g40200 | Ser/Thr protein phosphatase family protein, putative, expressed | mQTL 8.6 |
| 396 | LOC_Os09g06230 | serine/threonine-protein kinase 16, putative, expressed | mQTL 9.1 |
| 397 | LOC_Os09g11230 | Ser/Thr protein phosphatase family protein, putative, expressed | mQTL 9.2 |
| 398 | LOC_Os09g12240 | serine/threonine-protein kinase BRI1-like 1 precursor, putative, expressed | mQTL 9.2 |
| 399 | LOC_Os09g12300 | serine/threonine-protein kinase NAK, putative, expressed | mQTL 9.2 |
| 400 | LOC_Os09g11450 | transporter, monovalent cation:proton antiporter-2 family, putative, expressed | mQTL 9.2 |
| 401 | LOC_Os09g23640 | ABC-2 type transporter domain containing protein, expressed | mQTL 9.5 |
| 402 | LOC_Os09g25784 | auxin-induced protein 5NG4, putative, expressed | mQTL 9.5 |
| 403 | LOC_Os09g25800 | auxin-induced protein 5NG4, putative, expressed | mQTL 9.5 |
| 404 | LOC_Os09g25770 | auxin-induced protein 5NG4, putative, expressed | mQTL 9.5 |
| 405 | LOC_Os09g22000 | hydrolase, HAD superfamily, Cof family, putative, expressed | mQTL 9.5 |
| 406 | LOC_Os09g25850 | WAX2, putative, expressed | mQTL 9.5 |
| 407 | LOC_Os09g25070 | WRKY62, expressed | mQTL 9.5 |
| 408 | LOC_Os09g25060 | WRKY76, expressed | mQTL 9.5 |
| 409 | LOC_Os09g32984 | auxin responsive protein, putative, expressed | mQTL 9.6 |
| 410 | LOC_Os09g34860 | hydrolase, alpha/beta fold family domain containing protein, expressed | mQTL 9.6 |
| 411 | LOC_Os09g35700 | LTPL45 - Protease inhibitor/seed storage/LTP family protein precursor, expressed | mQTL 9.6 |
| 412 | LOC_Os09g32964 | peroxidase precursor, putative, expressed | mQTL 9.6 |
| 413 | LOC_Os09g33850 | sFTL4 FT-Like4 homologous to Flowering Locus T gene; contains Pfam profile PF01161: Phosphatidylethanolamine-binding protein, expressed | mQTL 9.6 |
| 414 | LOC_Os09g39910 | ABC transporter, ATP-binding protein, putative, expressed | mQTL 9.7 |
| 415 | LOC_Os09g38130 | auxin efflux carrier component, putative, expressed | mQTL 9.7 |
| 416 | LOC_Os09g32770 | auxin efflux carrier component, putative, expressed | mQTL 9.7 |
| 417 | LOC_Os09g38210 | auxin efflux carrier component, putative, expressed | mQTL 9.7 |
| 418 | LOC_Os09g32984 | auxin responsive protein, putative, expressed | mQTL 9.7 |
| 419 | LOC_Os09g37495 | auxin responsive protein, putative, expressed | mQTL 9.7 |
| 420 | LOC_Os09g37958 | cupin domain containing protein, expressed | mQTL 9.7 |
| 421 | LOC_Os09g37967 | cupin domain containing protein, expressed | mQTL 9.7 |
| 422 | LOC_Os09g37976 | cupin domain containing protein, expressed | mQTL 9.7 |
| 423 | LOC_Os09g39510 | Cupin domain containing protein, expressed | mQTL 9.7 |
| 424 | LOC_Os09g39520 | Cupin domain containing protein, expressed | mQTL 9.7 |
| 425 | LOC_Os09g39530 | Cupin domain containing protein, expressed | mQTL 9.7 |
| 426 | LOC_Os09g38755 | E3 ubiquitin-protein ligase HERC2, putative, expressed | mQTL 9.7 |
| 427 | LOC_Os09g36880 | GDSL-like lipase/acylhydrolase, putative, expressed | mQTL 9.7 |
| 428 | LOC_Os09g39430 | GDSL-like lipase/acylhydrolase, putative, expressed | mQTL 9.7 |
| 429 | LOC_Os09g34860 | hydrolase, alpha/beta fold family domain containing protein, expressed | mQTL 9.7 |
| 430 | LOC_Os09g38040 | hydrolase, NUDIX family protein, expressed | mQTL 9.7 |
| 431 | LOC_Os09g35700 | LTPL45 - Protease inhibitor/seed storage/LTP family protein precursor, expressed | mQTL 9.7 |
| 432 | LOC_Os09g37610 | MATE efflux protein, putative, expressed | mQTL 9.7 |
| 433 | LOC_Os09g33850 | osFTL4 FT-Like4 homologous to Flowering Locus T gene; contains Pfam profile PF01161: Phosphatidylethanolamine-binding protein, expressed | mQTL 9.7 |
| 434 | LOC_Os09g37330 | OsSAUR39 - Auxin-responsive SAUR gene family member, expressed | mQTL 9.7 |
| 435 | LOC_Os09g37350 | OsSAUR40 - Auxin-responsive SAUR gene family member, expressed | mQTL 9.7 |
| 436 | LOC_Os09g37369 | OsSAUR41 - Auxin-responsive SAUR gene family member, expressed | mQTL 9.7 |
| 437 | LOC_Os09g37380 | OsSAUR42 - Auxin-responsive SAUR gene family member, expressed | mQTL 9.7 |
| 438 | LOC_Os09g37390 | OsSAUR43 - Auxin-responsive SAUR gene family member, expressed | mQTL 9.7 |
| 439 | LOC_Os09g37394 | OsSAUR44 - Auxin-responsive SAUR gene family member, expressed | mQTL 9.7 |
| 440 | LOC_Os09g37400 | OsSAUR45 - Auxin-responsive SAUR gene family member, expressed | mQTL 9.7 |
| 441 | LOC_Os09g37410 | OsSAUR46 - Auxin-responsive SAUR gene family member, expressed | mQTL 9.7 |
| 442 | LOC_Os09g37420 | OsSAUR47 - Auxin-responsive SAUR gene family member, expressed | mQTL 9.7 |
| 443 | LOC_Os09g37430 | OsSAUR48 - Auxin-responsive SAUR gene family member, expressed | mQTL 9.7 |
| 444 | LOC_Os09g37440 | OsSAUR49 - Auxin-responsive SAUR gene family member, expressed | mQTL 9.7 |
| 445 | LOC_Os09g37452 | OsSAUR50 - Auxin-responsive SAUR gene family member | mQTL 9.7 |
| 446 | LOC_Os09g37460 | OsSAUR51 - Auxin-responsive SAUR gene family member, expressed | mQTL 9.7 |
| 447 | LOC_Os09g37470 | OsSAUR52 - Auxin-responsive SAUR gene family member, expressed | mQTL 9.7 |
| 448 | LOC_Os09g37480 | OsSAUR53 - Auxin-responsive SAUR gene family member, expressed | mQTL 9.7 |
| 449 | LOC_Os09g37490 | OsSAUR54 - Auxin-responsive SAUR gene family member, expressed | mQTL 9.7 |
| 450 | LOC_Os09g37500 | OsSAUR55 - Auxin-responsive SAUR gene family member, expressed | mQTL 9.7 |
| 451 | LOC_Os09g32944 | OsSPL18 - SBP-box gene family member, expressed | mQTL 9.7 |
| 452 | LOC_Os09g36110 | OsSub59 - Putative Subtilisin homologue, expressed | mQTL 9.7 |
| 453 | LOC_Os09g38800 | OsWAK88 - OsWAK pseudogene, expressed | mQTL 9.7 |
| 454 | LOC_Os09g38830 | OsWAK89a - OsWAK receptor-like protein kinase, expressed | mQTL 9.7 |
| 455 | LOC_Os09g38834 | OsWAK89b - OsWAK receptor-like protein kinase, expressed | mQTL 9.7 |
| 456 | LOC_Os09g38840 | OsWAK90 - OsWAK receptor-like protein kinase, expressed | mQTL 9.7 |
| 457 | LOC_Os09g38850 | OsWAK91 - OsWAK receptor-like protein kinase, expressed | mQTL 9.7 |
| 458 | LOC_Os09g38910 | OsWAK92 - OsWAK receptor-like protein kinase, expressed | mQTL 9.7 |
| 459 | LOC_Os09g39390 | oxidoreductase, aldo/keto reductase family protein, putative, expressed | mQTL 9.7 |
| 460 | LOC_Os09g37360 | pectinesterase, putative, expressed | mQTL 9.7 |
| 461 | LOC_Os09g39760 | pectinesterase, putative, expressed | mQTL 9.7 |
| 462 | LOC_Os09g32964 | peroxidase precursor, putative, expressed | mQTL 9.7 |
| 463 | LOC_Os09g36290 | Ser/Thr protein phosphatase family protein, putative, expressed | mQTL 9.7 |
| 464 | LOC_Os09g37800 | serine/threonine kinase, putative, expressed | mQTL 9.7 |
| 465 | LOC_Os09g37780 | serine/threonine-protein kinase receptor precursor, putative, expressed | mQTL 9.7 |
| 466 | LOC_Os09g37880 | serine/threonine-protein kinase receptor precursor, putative, expressed | mQTL 9.7 |
| 467 | LOC_Os09g37890 | serine/threonine-protein kinase receptor precursor, putative, expressed | mQTL 9.7 |
| 468 | LOC_Os09g37834 | serine/threonine-protein kinase receptor precursor, putative, expressed | mQTL 9.7 |
| 469 | LOC_Os09g37949 | serine/threonine-protein kinase SRPK1, putative, expressed | mQTL 9.7 |
| 470 | LOC_Os09g37300 | Transporter, monovalent cation:proton antiporter-2 family, putative, expressed | mQTL 9.7 |
| 471 | LOC_Os09g39910 | ABC transporter, ATP-binding protein, putative, expressed | mQTL 9.8 |
| 472 | LOC_Os09g38130 | auxin efflux carrier component, putative, expressed | mQTL 9.8 |
| 473 | LOC_Os09g38210 | auxin efflux carrier component, putative, expressed | mQTL 9.8 |
| 474 | LOC_Os09g37495 | auxin responsive protein, putative, expressed | mQTL 9.8 |
| 475 | LOC_Os09g37958 | cupin domain containing protein, expressed | mQTL 9.8 |
| 476 | LOC_Os09g37967 | cupin domain containing protein, expressed | mQTL 9.8 |
| 477 | LOC_Os09g37976 | cupin domain containing protein, expressed | mQTL 9.8 |
| 478 | LOC_Os09g39510 | Cupin domain containing protein, expressed | mQTL 9.8 |
| 479 | LOC_Os09g39520 | Cupin domain containing protein, expressed | mQTL 9.8 |
| 480 | LOC_Os09g39530 | Cupin domain containing protein, expressed | mQTL 9.8 |
| 481 | LOC_Os09g38755 | E3 ubiquitin-protein ligase HERC2, putative, expressed | mQTL 9.8 |
| 482 | LOC_Os09g39430 | GDSL-like lipase/acylhydrolase, putative, expressed | mQTL 9.8 |
| 483 | LOC_Os09g38040 | hydrolase, NUDIX family protein, expressed | mQTL 9.8 |
| 484 | LOC_Os09g37610 | MATE efflux protein, putative, expressed | mQTL 9.8 |
| 485 | LOC_Os09g37330 | OsSAUR39 - Auxin-responsive SAUR gene family member, expressed | mQTL 9.8 |
| 486 | LOC_Os09g37350 | OsSAUR40 - Auxin-responsive SAUR gene family member, expressed | mQTL 9.8 |
| 487 | LOC_Os09g37380 | OsSAUR42 - Auxin-responsive SAUR gene family member, expressed | mQTL 9.8 |
| 488 | LOC_Os09g37390 | OsSAUR43 - Auxin-responsive SAUR gene family member, expressed | mQTL 9.8 |
| 489 | LOC_Os09g37394 | OsSAUR44 - Auxin-responsive SAUR gene family member, expressed | mQTL 9.8 |
| 490 | LOC_Os09g37400 | OsSAUR45 - Auxin-responsive SAUR gene family member, expressed | mQTL 9.8 |
| 491 | LOC_Os09g37410 | OsSAUR46 - Auxin-responsive SAUR gene family member, expressed | mQTL 9.8 |
| 492 | LOC_Os09g37420 | OsSAUR47 - Auxin-responsive SAUR gene family member, expressed | mQTL 9.8 |
| 493 | LOC_Os09g37430 | OsSAUR48 - Auxin-responsive SAUR gene family member, expressed | mQTL 9.8 |
| 494 | LOC_Os09g37440 | OsSAUR49 - Auxin-responsive SAUR gene family member, expressed | mQTL 9.8 |
| 495 | LOC_Os09g37452 | OsSAUR50 - Auxin-responsive SAUR gene family member | mQTL 9.8 |
| 496 | LOC_Os09g37460 | OsSAUR51 - Auxin-responsive SAUR gene family member, expressed | mQTL 9.8 |
| 497 | LOC_Os09g37470 | OsSAUR52 - Auxin-responsive SAUR gene family member, expressed | mQTL 9.8 |
| 498 | LOC_Os09g37480 | OsSAUR53 - Auxin-responsive SAUR gene family member, expressed | mQTL 9.8 |
| 499 | LOC_Os09g37490 | OsSAUR54 - Auxin-responsive SAUR gene family member, expressed | mQTL 9.8 |
| 500 | LOC_Os09g37500 | OsSAUR55 - Auxin-responsive SAUR gene family member, expressed | mQTL 9.8 |
| 501 | LOC_Os09g38800 | OsWAK88 - OsWAK pseudogene, expressed | mQTL 9.8 |
| 502 | LOC_Os09g38830 | OsWAK89a - OsWAK receptor-like protein kinase, expressed | mQTL 9.8 |
| 503 | LOC_Os09g38834 | OsWAK89b - OsWAK receptor-like protein kinase, expressed | mQTL 9.8 |
| 504 | LOC_Os09g38840 | OsWAK90 - OsWAK receptor-like protein kinase, expressed | mQTL 9.8 |
| 505 | LOC_Os09g38850 | OsWAK91 - OsWAK receptor-like protein kinase, expressed | mQTL 9.8 |
| 506 | LOC_Os09g38910 | OsWAK92 - OsWAK receptor-like protein kinase, expressed | mQTL 9.8 |
| 507 | LOC_Os09g39390 | oxidoreductase, aldo/keto reductase family protein, putative, expressed | mQTL 9.8 |
| 508 | LOC_Os09g37360 | pectinesterase, putative, expressed | mQTL 9.8 |
| 509 | LOC_Os09g39760 | pectinesterase, putative, expressed | mQTL 9.8 |
| 510 | LOC_Os09g37800 | serine/threonine kinase, putative, expressed | mQTL 9.8 |
| 511 | LOC_Os09g37780 | serine/threonine-protein kinase receptor precursor, putative, expressed | mQTL 9.8 |
| 512 | LOC_Os09g37880 | serine/threonine-protein kinase receptor precursor, putative, expressed | mQTL 9.8 |
| 513 | LOC_Os09g37890 | serine/threonine-protein kinase receptor precursor, putative, expressed | mQTL 9.8 |
| 514 | LOC_Os09g37834 | serine/threonine-protein kinase receptor precursor, putative, expressed | mQTL 9.8 |
| 515 | LOC_Os09g37949 | serine/threonine-protein kinase SRPK1, putative, expressed | mQTL 9.8 |
| 516 | LOC_Os09g37300 | transporter, monovalent cation:proton antiporter-2 family, putative, expressed | mQTL 9.8 |
| 517 | LOC_Os09g39910 | ABC transporter, ATP-binding protein, putative, expressed | mQTL 9.9 |
| 147 | LOC_Os10g02250 | OsWAK95 - OsWAK receptor-like protein kinase, expressed | mQTL 10.1 |
| 148 | LOC_Os10g02276 | OsWAK96 - OsWAK receptor-like protein kinase, expressed | mQTL 10.1 |
| 149 | LOC_Os10g02284 | OsWAK97 - OsWAK receptor-like protein kinase, expressed | mQTL 10.1 |
| 150 | LOC_Os10g02360 | OsWAK98 - OsWAK receptor-like cytoplasmic kinase OsWAK-RLCK, expressed | mQTL 10.1 |
| 151 | LOC_Os10g02480 | oxidoreductase, aldo/keto reductase family protein, putative, expressed | mQTL 10.1 |
| 152 | LOC_Os10g02490 | oxidoreductase, aldo/keto reductase family protein, putative, expressed | mQTL 10.1 |
| 153 | LOC_Os10g02380 | oxidoreductase, aldo/keto reductase family protein, putative, expressed | mQTL 10.1 |
| 154 | LOC_Os10g01760 | peroxidase precursor, putative, expressed | mQTL 10.1 |
| 155 | LOC_Os10g02040 | peroxidase precursor, putative, expressed | mQTL 10.1 |
| 156 | LOC_Os10g02070 | peroxidase precursor, putative, expressed | mQTL 10.1 |
| 157 | LOC_Os10g02500 | serine/threonine-protein kinase BRI1-like 2 precursor, putative, expressed | mQTL 10.1 |
| 158 | LOC_Os10g01560 | serine/threonine-protein kinase, putative, expressed | mQTL 10.1 |
| 159 | LOC_Os10g22600 | ethylene-responsive transcription factor, putative, expressed | mQTL 10.2 |
| 160 | LOC_Os10g40200 | aminotransferase domain containing protein, putative, expressed | mQTL 10.3 |
| 161 | LOC_Os10g41150 | aminotransferase, classes I and II, domain containing protein, expressed | mQTL 10.3 |
| 162 | LOC_Os10g40420 | LTPL138 - Protease inhibitor/seed storage/LTP family protein precursor, expressed | mQTL 10.3 |
| 163 | LOC_Os10g40430 | LTPL139 - Protease inhibitor/seed storage/LTP family protein precursor, expressed | mQTL 10.3 |
| 164 | LOC_Os10g40440 | LTPL140 - Protease inhibitor/seed storage/LTP family protein precursor, expressed | mQTL 10.3 |
| 165 | LOC_Os10g40460 | LTPL141 - Protease inhibitor/seed storage/LTP family protein precursor, expressed | mQTL 10.3 |
| 166 | LOC_Os10g40470 | LTPL142 - Protease inhibitor/seed storage/LTP family protein precursor, putative, expressed | mQTL 10.3 |
| 167 | LOC_Os10g40480 | LTPL143 - Protease inhibitor/seed storage/LTP family protein precursor, expressed | mQTL 10.3 |
| 168 | LOC_Os10g40510 | LTPL144 - Protease inhibitor/seed storage/LTP family protein precursor, expressed | mQTL 10.3 |
| 169 | LOC_Os10g40520 | LTPL145 - Protease inhibitor/seed storage/LTP family protein precursor, expressed | mQTL 10.3 |
| 170 | LOC_Os10g40530 | LTPL146 - Protease inhibitor/seed storage/LTP family protein precursor, expressed | mQTL 10.3 |
| 171 | LOC_Os10g40614 | LTPL147 - Protease inhibitor/seed storage/LTP family protein precursor, expressed | mQTL 10.3 |
| 172 | LOC_Os10g41720 | peroxidase precursor, putative, expressed | mQTL 10.3 |
| 173 | LOC_Os11g05070 | sodium/calcium exchanger protein, putative, expressed | mQTL 11.1 |
| 174 | LOC_Os11g18870 | osFTL11 FT-Like11 homologous to Flowering Locus T gene; contains Pfam profile PF01161: Phosphatidylethanolamine-binding protein, expressed | mQTL 11.2 |
| 175 | LOC_Os12g04000 | auxin efflux carrier component, putative, expressed | mQTL 12.1 |
| 176 | LOC_Os12g07190 | CBS domain-containing protein, putative, expressed | mQTL 12.1 |
| 177 | LOC_Os12g05840 | Cupin domain containing protein, expressed | mQTL 12.1 |
| 178 | LOC_Os12g05860 | Cupin domain containing protein, expressed | mQTL 12.1 |
| 179 | LOC_Os12g05870 | Cupin domain containing protein, expressed | mQTL 12.1 |
| 180 | LOC_Os12g05880 | Cupin domain containing protein, expressed | mQTL 12.1 |
| 181 | LOC_Os12g06200 | E2F family transcription factor protein, putative, expressed | mQTL 12.1 |
| 182 | LOC_Os12g05600 | hydrolase, alpha/beta fold family protein, putative, expressed | mQTL 12.1 |
| 183 | LOC_Os12g05020 | jasmonate-induced protein, putative, expressed | mQTL 12.1 |
| 184 | LOC_Os12g03899 | major facilitator superfamily antiporter, putative, expressed | mQTL 12.1 |
| 185 | LOC_Os12g03950 | major facilitator superfamily antiporter, putative, expressed | mQTL 12.1 |
| 186 | LOC_Os12g06050 | MATE, putative, expressed | mQTL 12.1 |
| 187 | LOC_Os12g05590 | RCN3 Centroradialis-like1 homogous to TFL1 gene; contains Pfam profile PF01161: Phosphatidylethanolamine-binding protein, expressed | mQTL 12.1 |
| 188 | LOC_Os12g05540 | Ser/Thr protein phosphatase family protein, putative, expressed | mQTL 12.1 |
| 189 | LOC_Os12g06800 | serine esterase family protein, putative, expressed | mQTL 12.1 |
| 190 | LOC_Os12g04200 | GRAS family transcription factor containing protein, expressed | mQTL 12.1 |
| 191 | LOC_Os12g04370 | GRAS family transcription factor containing protein, expressed | mQTL 12.1 |
| 192 | LOC_Os12g06540 | GRAS family transcription factor containing protein, expressed | mQTL 12.1 |
| 193 | LOC_Os12g04380 | GRAS family transcription factor containing protein, expressed | mQTL 12.1 |
| 194 | LOC_Os12g22110 | ABC-2 type transporter, putative, expressed | mQTL 12.2 |
| 195 | LOC_Os12g18900 | aminotransferase, putative, expressed | mQTL 12.2 |
| 196 | LOC_Os12g17570 | GDSL-like lipase/acylhydrolase, putative, expressed | mQTL 12.2 |
| 197 | LOC_Os12g22030 | serine hydroxymethyltransferase, mitochondrial precursor, putative, expressed | mQTL 12.2 |
| 198 | LOC_Os12g34450 | auxin-binding protein 4 precursor, putative, expressed | mQTL 12.4 |
| 199 | LOC_Os12g32960 | LTPL148 - Protease inhibitor/seed storage/LTP family protein precursor, putative, expressed | mQTL 12.4 |
| 200 | LOC_Os12g34524 | peroxidase precursor, putative, expressed | mQTL 12.4 |
| 201 | LOC_Os12g32250 | WRKY96, expressed | mQTL 12.4 |
| 202 | LOC_Os12g37580 | ABC transporter, ATP-binding protein, putative, expressed | mQTL 12.5 |
| 203 | LOC_Os12g37490 | acetyltransferase, GNAT family, putative, expressed | mQTL 12.5 |
| 204 | LOC_Os12g37910 | GDSL-like lipase/acylhydrolase, putative, expressed | mQTL 12.5 |
| 205 | LOC_Os12g37660 | pectinesterase, putative, expressed | mQTL 12.5 |
| 206 | LOC_Os12g42280 | 9-cis-epoxycarotenoid dioxygenase 1, chloroplast precursor, putative, expressed | mQTL 12.6 |
| 207 | LOC_Os12g37580 | ABC transporter, ATP-binding protein, putative, expressed | mQTL 12.6 |
| 208 | LOC_Os12g37490 | acetyltransferase, GNAT family, putative, expressed | mQTL 12.6 |
| 209 | LOC_Os12g41950 | auxin response factor, putative, expressed | mQTL 12.6 |
| 210 | LOC_Os12g34450 | auxin-binding protein 4 precursor, putative, expressed | mQTL 12.6 |
| 211 | LOC_Os12g37910 | GDSL-like lipase/acylhydrolase, putative, expressed | mQTL 12.6 |
| 212 | LOC_Os12g43970 | hydrolase, alpha/beta fold family domain containing protein, expressed | mQTL 12.6 |
| 213 | LOC_Os12g34990 | hydrolase, putative, expressed | mQTL 12.6 |
| 214 | LOC_Os12g32960 | LTPL148 - Protease inhibitor/seed storage/LTP family protein precursor, putative, expressed | mQTL 12.6 |
| 215 | LOC_Os12g42130 | MATE efflux family protein, putative, expressed | mQTL 12.6 |
| 216 | LOC_Os12g41600 | OsSAUR57 - Auxin-responsive SAUR gene family member, expressed | mQTL 12.6 |
| 217 | LOC_Os12g43110 | OsSAUR58 - Auxin-responsive SAUR gene family member, expressed | mQTL 12.6 |
| 218 | LOC_Os12g42040 | OsWAK126 - OsWAK receptor-like protein kinase, expressed | mQTL 12.6 |
| 219 | LOC_Os12g42044 | OsWAK127b - OsWAK short gene, expressed | mQTL 12.6 |
| 220 | LOC_Os12g42060 | OsWAK128b - OsWAK receptor-like protein kinase, expressed | mQTL 12.6 |
| 221 | LOC_Os12g42070 | OsWAK129b - OsWAK receptor-like protein kinase, expressed | mQTL 12.6 |
| 222 | LOC_Os12g42064 | OsWAK129c - OsWAK receptor-like protein kinase, expressed | mQTL 12.6 |
| 223 | LOC_Os12g37660 | pectinesterase, putative, expressed | mQTL 12.6 |
| 224 | LOC_Os12g34524 | peroxidase precursor, putative, expressed | mQTL 12.6 |
| 225 | LOC_Os12g42300 | potasium efflux antiporter protein, putative, expressed | mQTL 12.6 |
| 226 | LOC_Os12g44020 | Ser/Thr protein phosphatase family protein, putative, expressed | mQTL 12.6 |
| 227 | LOC_Os12g44330 | serine/threonine-protein kinase PRP4, putative, expressed | mQTL 12.6 |
| 228 | LOC_Os12g42310 | serine/threonine-protein phosphatase BSL2, putative, expressed | mQTL 12.6 |
| 229 | LOC_Os12g42910 | sodium/calcium exchanger protein, putative, expressed | mQTL 12.6 |
| 230 | LOC_Os12g44360 | sodium/hydrogen exchanger 7, putative, expressed | mQTL 12.6 |
| 231 | LOC_Os12g40570 | WRKY94, expressed | mQTL 12.6 |
| 232 | LOC_Os12g32250 | WRKY96, expressed | mQTL 12.6 |
